# Modulation of ERCC1-XPF Heterodimerization Inhibition *via* Structural Modification of Small Molecule Inhibitor Side-Chains

**DOI:** 10.3389/fonc.2022.819172

**Published:** 2022-03-17

**Authors:** Claudia Weilbeer, David Jay, James C. Donnelly, Francesco Gentile, Feridoun Karimi-Busheri, Xiaoyan Yang, Rajam S. Mani, Yaping Yu, Ahmed H. Elmenoufy, Khaled H. Barakat, Jack A. Tuszynski, Michael Weinfeld, Frederick G. West

**Affiliations:** ^1^ Department of Chemistry, University of Alberta, Edmonton, AB, Canada; ^2^ Department of Oncology, Cross Cancer Institute, University of Alberta, Edmonton, AB, Canada; ^3^ Department of Physics, University of Alberta, Edmonton, AB, Canada; ^4^ Centre for Genome Engineering, University of Calgary, Calgary, AB, Canada; ^5^ Department of Pharmaceutical Chemistry, College of Pharmacy, Misr University for Science and Technology, 6th of October City, Egypt; ^6^ Faculty of Pharmacy and Pharmaceutical Sciences, University of Alberta, Edmonton, AB, Canada; ^7^ Cancer Research Institute of Northern Alberta, University of Alberta, Edmonton, AB, Canada

**Keywords:** DNA repair, ERCC1-XPF small molecule inhibitors, computer aided drug design (CADD), proximity ligation assay, ionizing and UV irradiation

## Abstract

Inhibition of DNA repair enzymes is an attractive target for increasing the efficacy of DNA damaging chemotherapies. The ERCC1-XPF heterodimer is a key endonuclease in numerous single and double strand break repair processes, and inhibition of the heterodimerization has previously been shown to sensitize cancer cells to DNA damage. In this work, the previously reported ERCC1-XPF inhibitor 4 was used as the starting point for an *in silico* study of further modifications of the piperazine side-chain. A selection of the best scoring hits from the *in silico* screen were synthesized using a late stage functionalization strategy which should allow for further iterations of this class of inhibitors to be readily synthesized. Of the synthesized compounds, compound 6 performed the best in the *in vitro* fluorescence based endonuclease assay. The success of compound 6 in inhibiting ERCC1-XPF endonuclease activity *in vitro* translated well to cell-based assays investigating the inhibition of nucleotide excision repair and disruption of heterodimerization. Subsequently compound 6 was shown to sensitize HCT-116 cancer cells to treatment with UVC, cyclophosphamide, and ionizing radiation. This work serves as an important step towards the synergistic use of DNA repair inhibitors with chemotherapeutic drugs.

## Introduction

DNA damage has been implicated in causing cancer and other diseases related to aging (1). Processes that repair DNA damage and the proteins responsible for repair play an important role in preserving human genetic material and preventing these diseases (2–7). The heterodimer ERCC1-XPF is central to both global genome and transcription coupled nucleotide excision repair (NER), which removes bulky adducts and lesions in DNA (8–12), and in replication dependent and independent interstrand crosslink (ICL) repair (13–19). It has also been suggested that ERCC1-XPF plays minor roles in various other single (20, 21) and double (22–25) strand break repair processes.

Inhibition of DNA repair enzymes in the treatment of cancer has had some success; the approval of poly (ADP-ribose) polymerase (PARP) inhibitors to treat BRCA deficient cancers was a revolutionary step in exploiting synthetic lethality to afford enhanced selectivity in homologous recombination (HR) defective cancers. The development of PARP inhibition and the exploration of targeting other DNA repair enzymes in the treatment of cancers has been comprehensively reviewed (26–30). Inhibition of ERCC1-XPF is likely to increase the efficacy of DNA damaging therapies. Since cyclobutane pyrimidine dimers (CPD) and pyrimidine-pyrimidone (6–4) adducts are formed from UV radiation (31–34) and are repaired by NER, it follows that NER deficient cells will be more susceptible to treatment with UV (35, 36). The same can be said for damage caused by DNA crosslinking chemotherapeutic drugs, such as mitomycin C and cisplatin, which is removed by ICL repair (35, 37). Further evidence for the requirement for therapies that target ERCC1-XPF comes from recent (2015-present) systematic reviews and meta-analyses that conclude that high expression of ERCC1 and/or XPF in tumors leads to poor prognoses, and low expression of ERCC1 and/or XPF in tumors leads to improved prognoses in a wide variety of different cancer types (38–48).

ERCC1-XPF is an obligate heterodimer (49–51), in which the XPF protein is responsible for the endonuclease activity and the ERCC1 protein is involved in protein-protein and DNA-protein interactions (52, 53). The endonuclease activity of ERCC1-XPF and the stability of both proteins being reliant on their heterodimerization (53–55), and their interactions with other proteins while repairing DNA, presents multiple opportunities to inhibit the repair of damaged DNA. So far efforts have been made to inhibit the XPF active site, the interaction of ERCC1 with XPA, and the heterodimerization of ERCC1 and XPF.

Initially, Tsodikov and co-workers (56) were able to inhibit ERCC1-XPA dimerization with a XPA peptide fragment. This loss of ERCC1-XPA binding led to loss of NER, which was the first proof of principle that an inhibitor targeting an NER protein-protein interaction could lead to loss of NER. Following the success of the XPA peptide, Barakat et al. (57, 58) carried out virtual screening to identify potential ERCC1-XPA inhibitors; of the hit compounds 14 were tested for their ability to bind to ERCC1 and to sensitize HCT-116 and A549 cells to UVC radiation. One inhibitor was found to sensitize HCT-116 cells to UVC radiation and synergize with cisplatin in HCT-116 cells. Gentile et al. have since carried out larger *in silico* screening to identify ERCC1-XPA inhibitors (59). XPF active site inhibitors have also attracted interest (60–65). Several of these compounds were shown to be capable of sensitizing cancer cells to cisplatin (60, 61, 63, 64).

**Figure 1:**
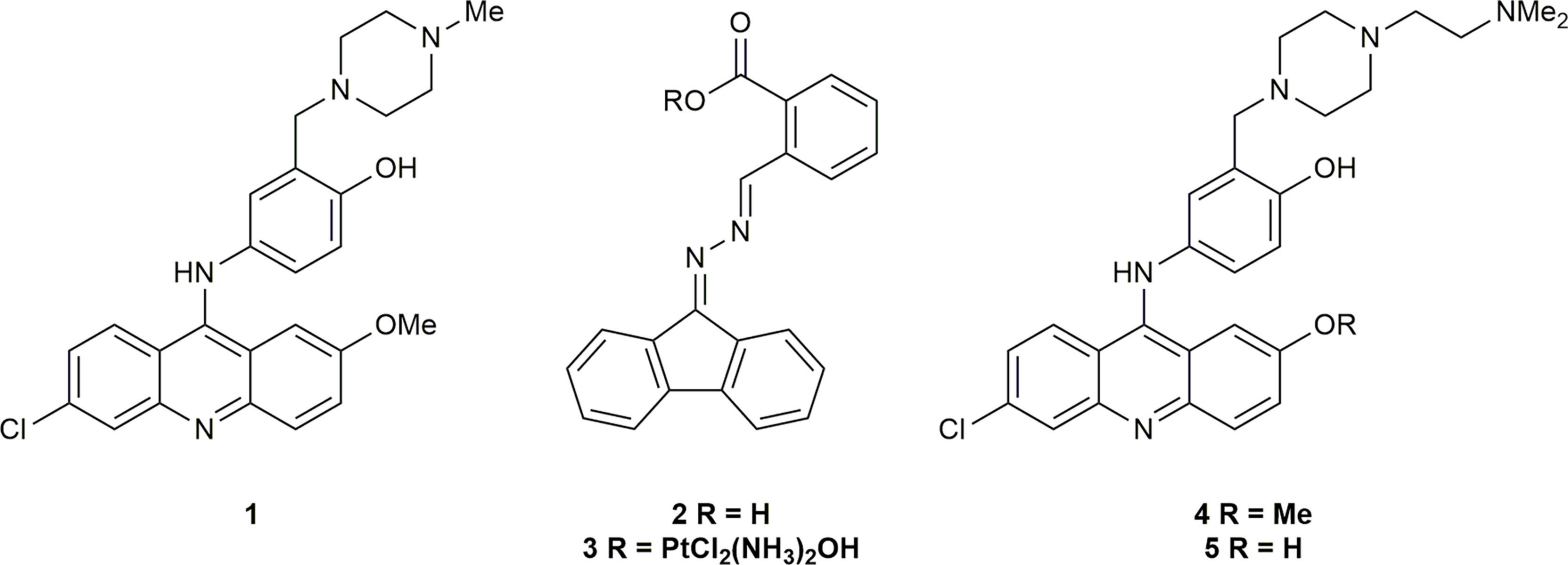
Previously reported inhibitors of ERCC1-XPF heterodimerization.

The inhibition of the heterodimerization of ERCC1 and XPF has been described as a “formidable target” (66); however, the instability of both proteins in the absence of heterodimerization (53–55) and the inability of incorrectly folded ERCC1-XPF to localize to the nucleus of damaged cells (67) makes it an attractive target to increase the susceptibility of tumor cells to DNA damaging chemotherapies. McNeil et al. (60) were able to identify an ERCC1-XPF inhibitor *via in silico*, surface plasmon resonance (SPR), and *in vivo* NER screening. This inhibitor was able to enhance the sensitivity of A375 human melanoma cells to cisplatin. Jordheim and co-workers (68) used binding energy decomposition analysis to identify three potential binding sites on XPF that could be inhibited. Of the residues studied the interaction of Phe293 of ERCC1 [previously identified as an important residue in ERCC1-XPF heterodimerization (52, 55)] with XPF had the largest contribution to the binding energy. As such, an *in silico* screen of inhibitors in that binding pocket in the C-terminal hairpin-helix-hairpin (HhH2) domain of XPF, and subsequent toxicity assay of the most promising hits identified inhibitors 1 and 2 (**Figure 1**). Compound 2 was later derivatized to 3, a prodrug which released cisplatin and 2 *in vivo*, that also inhibited DNA repair with some success (69). Compound 1 was shown to synergize with cisplatin and mitomycin C by interacting with ERCC1-XPF (68), and as such 1 was chosen as the basis for future inhibitors of ERCC1-XPF.

Following the successful inhibition of ERCC1-XPF and the synergy of compound 1 with chemotherapeutic drugs, Elmenoufy et al. (70) carried out *in silico* screening of inhibitors with differing piperazine substitutions, with the six best scoring entries being tested in an endonuclease assay. The most active compound (4, **Figure 1**) was then tested in HCT-116 cells and sensitized those cells to UVC radiation and cyclophosphamide treatment. Elmenoufy and co-workers (71) then investigated modifications of different sites on inhibitors 1 and 4. They found that inhibitor 5 (**Figure 1**) with the methyl group masking the phenolic OH removed led to an increase in activity, and sensitized HCT-116 cells to UVC radiation and cyclophosphamide treatment. Comprehensive molecular dynamics simulations were carried out on inhibitors 1 and 5 with a view to investigating further possible modifications to increase the efficacy of this class of inhibitors (72). Importantly, Ciniero et al. (73) were able to show that 5 sensitized HCT-116 and A549 cells to cisplatin and mitomycin C, they were also able to show, *via* a proximity ligation assay, that sensitization of A549 cells to cisplatin was due to inhibition of the heterodimerization of ERCC1-XPF.

**Figure 2:**
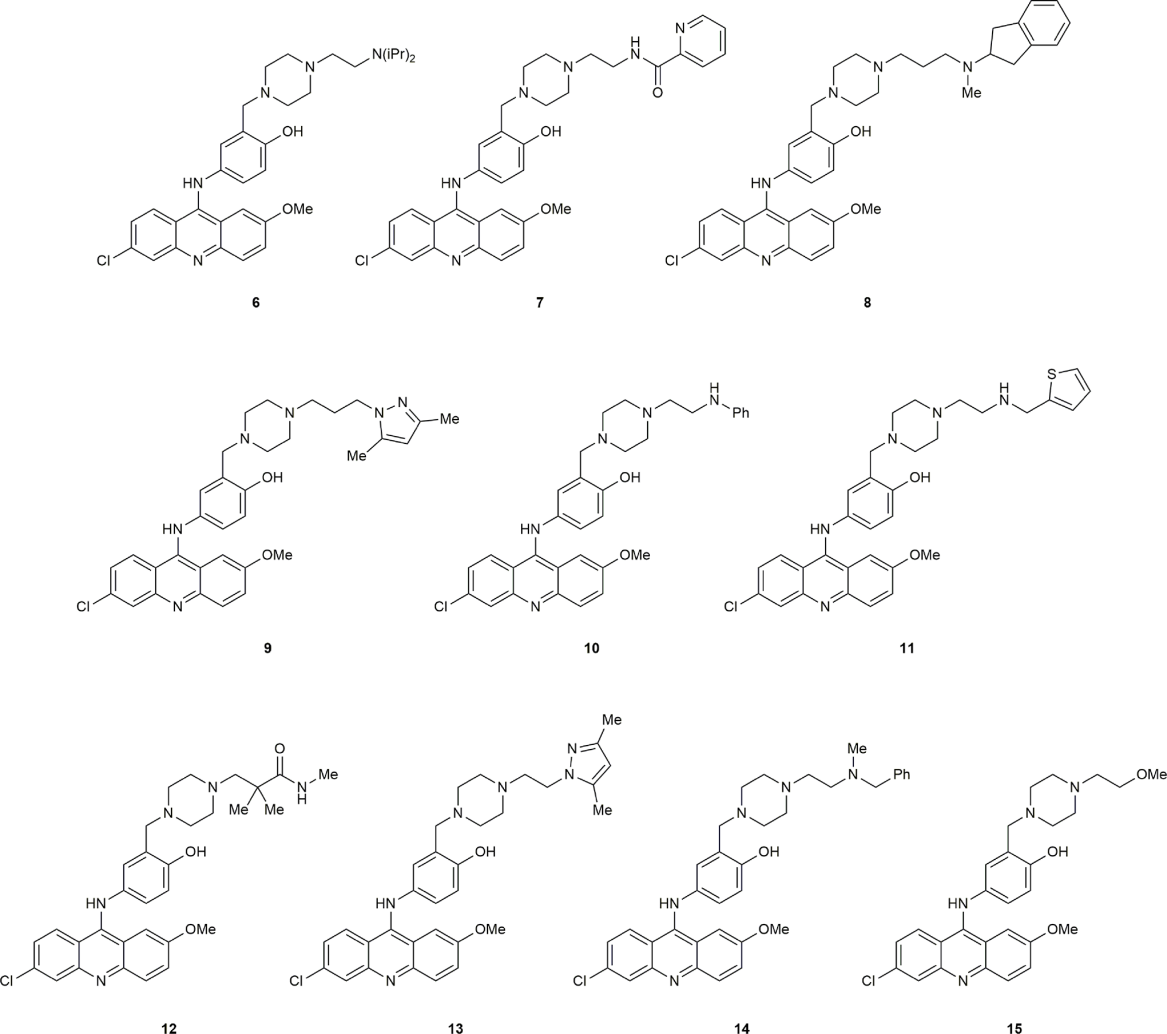
Structures of compounds from *in silico* screening results.

The substantial reduction in IC_50_ in comparing compound 4 with the initial hit 1 showed that modification of the piperazine side-chain could have significant effects on activity. The importance of a tertiary amine moiety at the terminus of the side-chain was clear, but systematic structural variation would be necessary to determine the optimal linker and steric demand of the nitrogen substituents. We therefore conducted another round of *in silico* screening, with focus on this side chain. These studies revealed a strong positive effect of diisopropyl substitution on the amino group, as shown in compound 6. Of the ten highest ranked hits (**Figure 2**) from the *in silico* screen, six were synthesized, including 6. Herein we report the design, synthesis, and biological evaluation of these novel inhibitors, among which 6 stands out as a promising hit.

## Materials and Methods

### Overview of *In Silico* Design Strategy for ERCC1–XPF Inhibitors

Molecular Operating Environment (MOE) Dock’s pharmacophore-assisted docking (74) was used in the initial step of the virtual screening procedure, using the same procedure previously described (72). 32 top-scoring compounds were selected, based on their Born Volume Integral/Weighted Surface Area (GBVI/WSA) docking score (75) and visual inspection of the docking poses, and average Mechanics/Generalized Born Surface Area (MM/GBSA) scores (76) were computed from 2-ns molecular dynamics simulations run as previously described (72). LogP values were calculated using the SlogP function in MOE (77).

### Synthesis of ERCC1-XPF Inhibitors

Full experimental procedures and characterization data can be found in the supporting information.

#### Synthesis of 6, 10 and 11

**Scheme 1:**
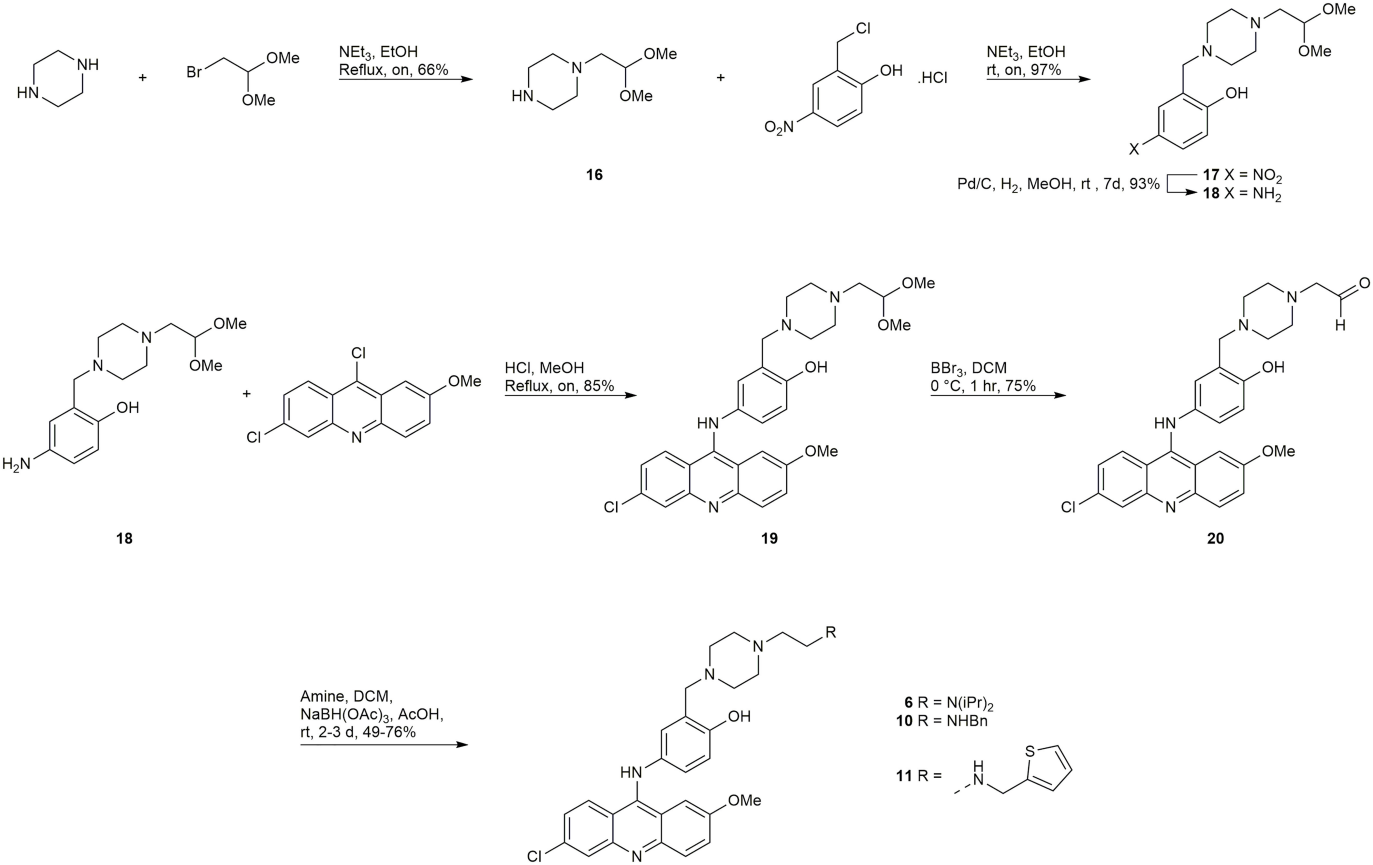
Synthesis of 6, 10, and 11.

A late-stage functionalization by reductive amination of intermediate 20 (**Scheme 1**) was employed in efforts to synthesize the inhibitors 6, 10, 11, 13, and 14. Synthesis began with a mono-alkylation of piperazine with the dimethyl acetal of bromoacetaldehyde to afford **16** in acceptable yield, which could be alkylated in excellent yield with 2-chloromethyl-4-nitrophenol to give 17 (**Scheme 1**). Compound 17 underwent reduction with Pd/C to generate 18, which could undergo a regioselective S_N_Ar reaction with 6,9-dichloro-2-methoxyacridine to provide 19, the dimethyl acetal of the required aldehyde 20. Treatment of 19 with BBr_3_ afforded the desired intermediate 20. Reductive aminations with diisopropylamine, benzylamine, and 2-thiophenemethylamine were successful in affording the inhibitors 6, 10, and 11, respectively (**Scheme 1**). Unfortunately, reductive aminations with the amines required to synthesize 13, and 14 were unable to afford material of sufficient quantity and/or purity for biological testing.

#### Synthesis of 8

**Scheme 2:**
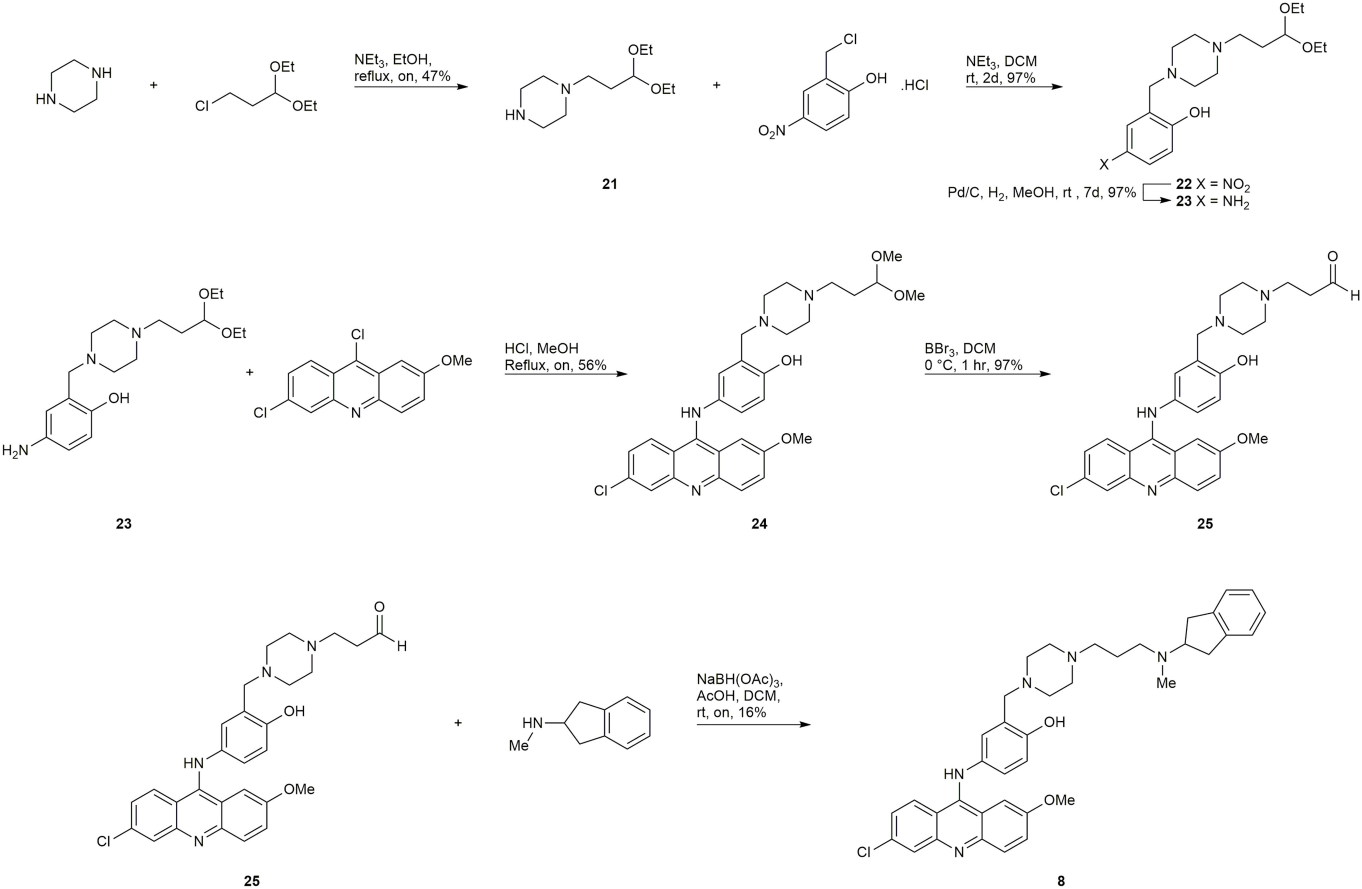
Synthesis of 8.

Starting from 3-chloropropionaldehyde diethyl acetal (later converted to the dimethyl acetal *in situ* during the S_N_Ar reaction), an analogous late-stage functionalization by reductive amination of intermediate 25 (**Scheme 2**) was employed to attempt to synthesize 8 and 9. The synthesis of compound 8 was successful, however, the synthesis of 9 was unsuccessful.

#### Synthesis of 12

**Scheme 3:**
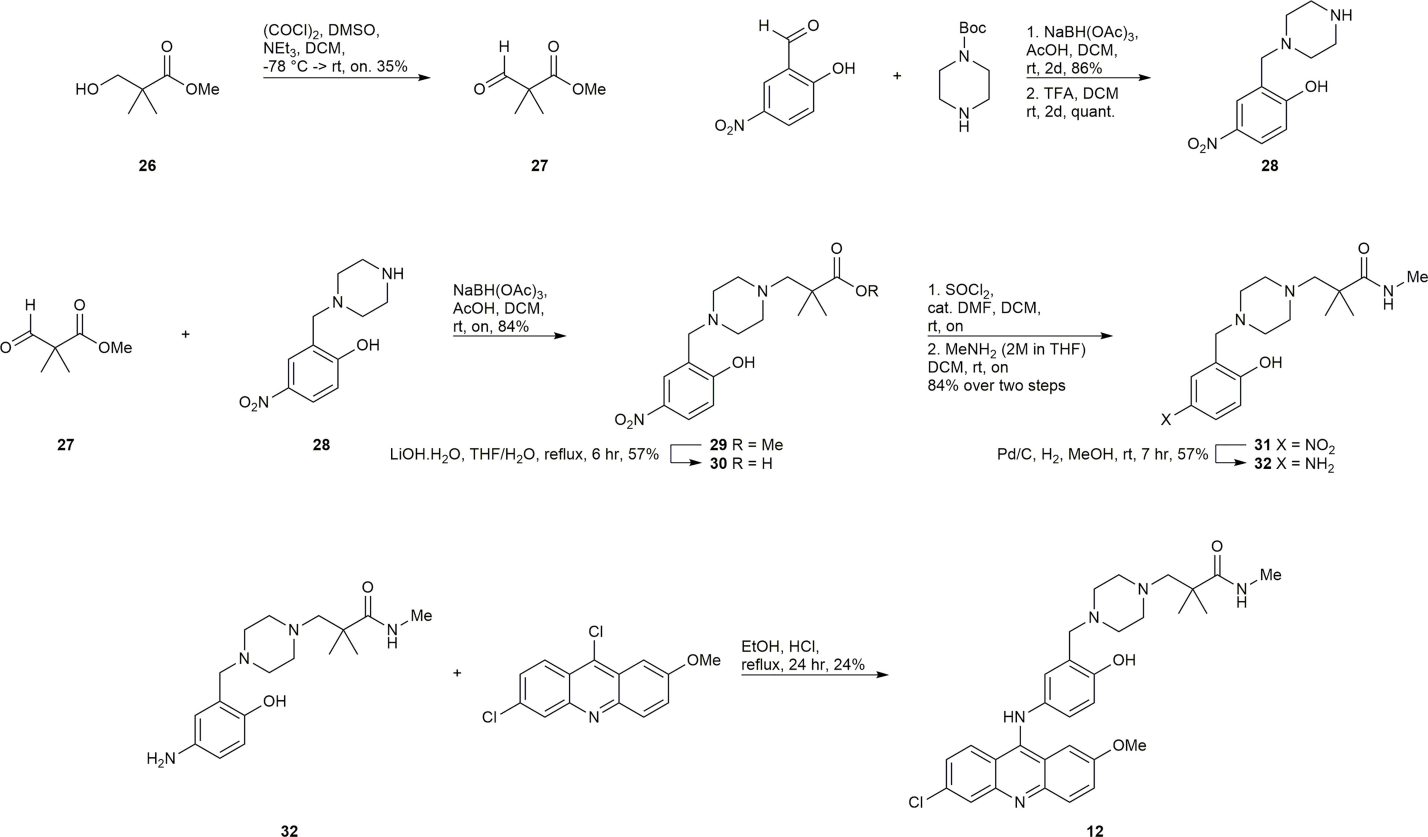
Synthesis of 12.

The synthesis of 12 presented the unique challenge of the geminal dimethyl group in the linker (**Scheme 3**). Commercially available 26 was subjected to the Swern modified Moffatt oxidation conditions to afford the aldehyde 27, which could undergo a reductive amination with the easily prepared amine 28 to afford intermediate 29. Compound 29 was a convenient intermediate to convert the methyl ester to the methyl amide *via* saponification, activation with SOCl_2_, and amidation with methylamine to afford compound 31. Reduction of 31 to 32 and subsequent S_N_Ar reaction with 6,9-dichloro-2-methoxyacridine gave the desired inhibitor 12.

#### Synthesis of 15

**Scheme 4:**
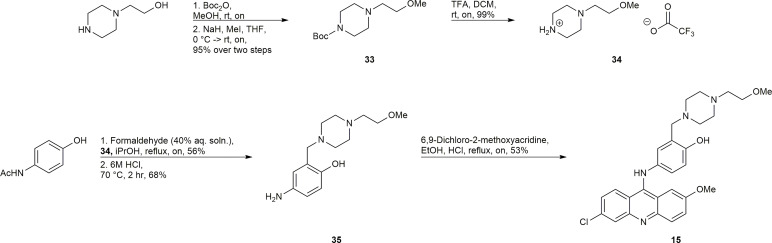
Synthesis of 15.

The synthesis of 15 is depicted in **Scheme 4**. Boc protection and methylation of 1-(2-hydroxyethyl)piperazine afforded compound 33. The deprotection of 33 with TFA afforded the TFA salt 34, which could undergo a multicomponent reaction with acetaminophen and formaldehyde, and subsequent acid hydrolysis to afford the required amine 35. Compound 35 underwent a S_N_Ar reaction with 6,9-dichloro-2-methoxyacridine to give 15.

### ERCC1-XPF Protein Preparation

Recombinant human ERCC1–XPF wild-type protein (containing polyhistidine (His-6) tags) was expressed from a bicistronic plasmid (kindly provided by Dr. Richard Wood, University of Texas MD Anderson Cancer Center, Smithville, TX) in the *E. coli* BL21(DE3) strain, Following previously described procedures (70, 78) the proteins extracted from *E. coli* were eluted from a ProBond Nickel-Chelating Resin (Thermo Fisher Scientific) and then a Hi-trap heparin column (GE Healthcare). Fractions containing ERCC1–XPF were dialyzed, concentrated, and stored at −80°C in 10 mM HEPES, pH 7.4, 2.5 mM β-mercaptoethanol, 0.01% CHAPS, 0.25 mM EDTA, 50% glycerol, and 25 mM NaCl. Based on polyacrylamide gel separation and Coomassie Blue staining, the final purity of the full-length ERCC1-XPF heterodimer was determined to be ~35%.

### Microplate Fluorescence Incision Assay

We employed a previously described protocol (60, 70, 78) in which the incision of the stem–loop substrate [6-FAM-5′-CAGCGCTCGG(20T)CCGAGCGCTG-3′-dabcyl] (100 nM in 50 mM Tris-HCl, pH 8, 20 mM NaCl, 0.5 mM DDT, and 0.75 mM MnCl_2_) mediated by ERCC1-XPF (25 ng) in a total volume of 20 μL at 25°C was monitored by fluorescence using a FLUOstar Optima fluorimeter (BMG Labtech) with Optima software and excitation and emission wavelengths of 485 and 520 nm, respectively, for 5.5 min. The final concentration of inhibitor in the reaction was 10 μM prepared from a 200 μM stock solution in DMSO. With a molecular weight of 151 KDa for the XPF-ERCC1 heterodimer, a concentration of 25 ng of protein in 20 µl reaction buffer and a purity of 35%, the molar concentration of the enzyme was estimated to be 2.9 nM. Data were plotted using Microsoft Excel 2016.

### Steady State Fluorescence Assays

Steady-state fluorescence spectra were measured at room temperature on a PerkinElmer LS-55 spectrofluorometer (Freemont, CA) as previously described (70). In studying the effects of inhibitors on protein fluorescence intensities, additions to protein samples were made from inhibitor stock solutions in DMSO, keeping the protein dilution below 3%. Data were plotted using GraphPad Prism version 5.04 software (San Diego, California)

### Cell Culture

Human HCT-116 colorectal cancer cells and A549 human lung cancer cells were purchased from the American Type Culture Collection (ATCC, Manassas, VA). Following expansion of the cell population immediately after arrival, aliquots were stored frozen in liquid nitrogen. Freshly thawed cells were used for each experiment. Cells were cultured in 1:1 DMEM/F12 media supplemented with 10% FBS, 50 units/mL penicillin, 50 μg/mL streptomycin, 2.5 mM l-glutamine, 0.1 mM nonessential amino acids, and 1 mM sodium pyruvate and maintained under 5% CO_2_ in a humidifier incubator at 37°C. All cell culture supplies were purchased from Gibco/BRL through ThermoFisher Scientific (Mississauga, ON). Information on CRISPR deletion of XPF from HCT-116 cells can be found in the **Supplementary Information**.

### Cellular Repair of Cyclobutane Pyrimidine Dimers

We followed the protocol of Mirzayans et al. (79) with minor modifications as previously described (70). Inhibitor compounds were dissolved in DMSO and applied to a final DMSO concentration of 0.2% in media and 0.2% DMSO was used as the vehicle control. Mouse anti-thymine dimer monoclonal antibody (cat. no. MC-062, Kamiya Biomedical Company, Seattle, WA) was used for the immunofluorescence following UVC irradiation of the cells and fluorescent microscopic evaluation and measurement of fluorescence intensity were performed using MetaXpress, version 6.2.1.704, software (Molecular Devices, Sunnyvale, California). Data were plotted using GraphPad Prism version 5.04 software.

### Proximity Ligation Assay

A previously published protocol (73) was followed with minor modifications. Briefly, 3 x 10^4^ A549 cells were seeded in each well of an 8-well Chamber Slide system (Ibidi. Fitchburg, WI) and allowed to adhere overnight. The cells were then treated with the inhibitor (2 μM prepared from a 1 mM stock in DMSO) or vehicle control (0.2% DMSO) and incubated for 24 h. Samples were fixed and processed for protein proximity ligation analysis (PLA) by the Duolink PLA assay kit (Sigma-Aldrich, Oakville, ON) using an ERCC1 antibody (A73368-100, 1/100; EpiGentek, Farmingdale, NY) and an XPF antibody (LS-C173159, 1/100; LifeSpan BioSciences, Seattle, WA). Nuclei were then stained with DAPI and the samples visualized using a laser scanning confocal microscope (ZEISS LSM710, Germany). Images of the interaction of ERCC1 and XPF represented as red dots were analyzed using IMARIS 9.7 software (Oxford Instruments). Results are expressed as mean values from at least three experiments conducted independently in duplicate. We confirmed that the addition of only one antibody, either ERCC1 or XPF did not elicit any signal nor did the use of a HCT 116 cell line deficient in XPF expression (data not shown).

### Clonogenic Survival Assays

#### UV Treatment

HCT-116 cells (100–800 cells depending on the UV dose) were plated in triplicate in 60-mm Petri dishes in DMEM/F12 medium. Following overnight attachment of the cells in a humidified atmosphere containing 5% CO_2_ at 37°C, the cells were treated with 0.5 μM compound 6 or 10 in DMSO (as described in section 2.7) for 4 h before the medium was removed, and the cells exposed to increasing doses (0–10 J/m^2^) of UV-C radiation. The cells were then cultured for a further 24 h in the presence of inhibitor and then for an additional 9 days in the absence of inhibitor to allow for colony formation. Colonies were stained with crystal violet and counted using a Colcount instrument (Oxford Optronix, Abingdon UK) to facilitate determination of plating efficiency and surviving fraction. Data were plotted using GraphPad Prism version 5.04 software.

#### Cyclophosphamide Treatment

A similar protocol was followed to that described above for the UV treatment. Cells were treated with 0.5 μM compound **6** or compound 10 in DMSO for 4 h followed by addition of increasing doses of cyclophosphamide (0–300 μM) and further incubation for 24 h. The medium was then replaced with drug and inhibitor-free fresh medium. After incubation for another 9 days to allow for colony formation, the plates were stained with crystal violet, colonies were counted, and plating efficiency and surviving fraction were calculated. Data were plotted using GraphPad Prism version 5.04. software.

#### Treatment With Ionizing Radiation

The effectiveness of compound 6 for sensitization of cells to ionizing radiation was also assessed using the clonogenic survival assay. Briefly, 200-3000 of HCT-116 cells (depending on the radiation dose) were seeded and after 24 hours the cells were pretreated with 0.5 or 1 µM of compound 6 in DMSO for 4 hours followed by exposure to increasing doses of γ-radiation (^60^Co Gammacell; Atomic Energy of Canada Limited, Ottawa) from 0 to 8 Gy (at a dose rate of 0.8 Gy/min) and kept for an additional 24 hours in inhibitor-containing medium. The medium was then replaced with fresh medium without the compound, and the plates were incubated at 37°C for 9 more days before staining and determining the number of colonies. Data were plotted using GraphPad Prism version 5.04 software.

### Pharmacokinetic Assessment

The ADME (absorption, distribution, metabolism, and excretion) profile of our lead compound 6 was determined by standard protocols carried out by WuXi AppTec (Shanghai) Co (https://www.wuxiapptec.com/). The following tests were conducted: distribution coefficient (log *D* at pH 7.4), aqueous solubility (Kinetic), metabolic stability in human liver microsomes and cryopreserved human hepatocytes, bidirectional permeability in Caco-2 cells, serum protein binding, and cytochrome P450 (CYP) inhibition (CYP1A2, CYP2C9, CYP2C19, CYP2D6, and CYP3A4-M).

## Results

### Identification of Piperazine Side-Chain Modifications *via In Silico* Screening

Our initial computational screening of modifications to the piperazine side chain revealed 32 hits. These 32 hits were subjected to molecular dynamics simulations; the compounds with the lowest computed binding affinities can be found in **Table 1**. It is clear from the relative scores for 1 and 4 (-17.78 and -13.12 kcal/mol, respectively) that the calculated binding affinity does not necessarily correlate to *in vivo* efficacy, however, the standout calculated binding affinity of -32.47 kcal/mol strongly suggested that 6 would be an effective inhibitor of ERCC1-XPF. Compounds 7-15 (**Figure 2**) all had similar calculated binding affinities to compounds 1 and 4, which suggested that those compounds would inhibit ERCC1-XPF to a degree similar to that of the parent compounds.

**Table 1:** *In silico* screening results: Average binding energies were calculated over a molecular dynamics trajectory using MM/GBSA method; cLog P values were determined in MOE using an empirical method based on single atom contributions.

Compound	MM/GBSA [kcal/mol]	cLog P
**6**	-32.47	5.58
**7**	-18.75	5.71
**8**	-17.63	4.54
**9**	-16.80	5.64
**10**	-16.19	7.0
**11**	-15.42	5.97
**12**	-15.15	4.24
**13**	-14.52	5.25
**14**	-13.32	4.45
**15**	-13.27	4.11
**1**	-17.78	4.10
**4**	-13.12	2.61

### Inhibition of ERCC1-XPF Endonuclease Activity

**Figure 3:**
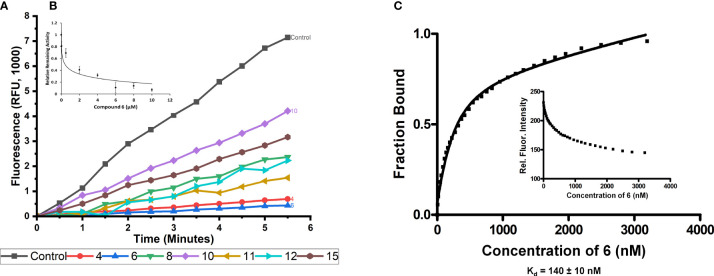
*In vitro* inhibition of ERCC1-XPF endonuclease activity and binding of compound 6 to ERCC1-XPF. **(A)** ERCC1-XPF mediated cleavage of the stem-loop DNA substrate, in which the FAM signal is quenched, releases the fluorescently tagged octanucleotide. A representative tracing of the effect of the different compounds (10 μM each) on the incision activity is shown. The inset **(B)** shows the initial velocities (slopes) obtained as indicated in **(A)** normalized by its value in the absence of compound, *vs.* its value in the presence of increasing micro molar concentrations of compound 6. The bars represent the S.D. of three different measurements for each point (R^2^ = 0.96). **(C)** Binding affinity (Kd) measurement between ERCC1-XPF complex and compound 6. Representative plot of ERCC1-XPF fluorescence quenching *vs* concentration of compound 6 to determine unimodal binding pattern (R^2^ = 0.98). Protein fluorescence was excited at 295 nm, and changes in fluorescence intensity were monitored at the emission maximum (330 nm). The Kd value of 140 ± 10 nM was determined from three independent plots.

An *in vitro* fluorescence-based assay was used to assess the ability of the synthesized compounds to inhibit the endonuclease activity of ERCC1-XPF. This assay has been previously described (60, 70, 78) and makes use of a stem-loop DNA substrate with a 5’-FAM fluorescent dye, and a 3’-dabcyl quencher. When ERCC1-XPF can cleave the DNA substrate, a 5’-FAM containing molecule is liberated from the stem-loop (and the quencher) and the fluorescence of the solution increases. The increase of the fluorescence of the solution can be observed in **Figure 3A**, in the absence of any inhibitor. To determine the influence of the inhibitory compounds, the protein was preincubated for 10 minutes with 10 μM of each inhibitor prior to addition of the substrate. It can be observed that while the synthesized inhibitors 8, 10, 11, 12, and 15 showed a decrease in fluorescence intensity when compared to the control, indicating some inhibition of ERCC1-XPF endonuclease activity, they were unable to improve on the inhibition shown by 4 (**Figure 3A**). However, in keeping with our computational predictions, compound 6 showed increased inhibition of ERCC1-XPF endonuclease activity relative to 4 and decreased the activity of ERCC1-XPF by 94% at 5.5 minutes (**Figure 3A**, **Supplementary Figure 1**).

Different concentrations of compound 6 were plotted against the relative remaining activity of the enzyme. From this plot (**Figure 3B**) an IC_50_ of 0.167 +/- 0.028 µM was calculated for compound 6.

### Compound 6 Binding to ERCC1-XPF

Investigation of the binding of compound 6 to ERCC1-XPF was carried out *via* intrinsic fluorescence spectroscopy. The tryptophan residues of ERCC1-XPF were irradiated at 295 nm and the emission at 330 nm was monitored. Upon addition of compound 6 quenching of the intrinsic fluorescence of the protein was observed, consistent with ligand binding to a protein. The binding affinity was determined by plotting emission at 330 nm against increasing concentration of compound 6 (**Figure 3C**). Nonlinear regression of the results was carried out, as previously described (70, 80), to calculate a K_d_ of 140 +/- 10 nM.

### Inhibition of Cellular NER

**Figure 4:**
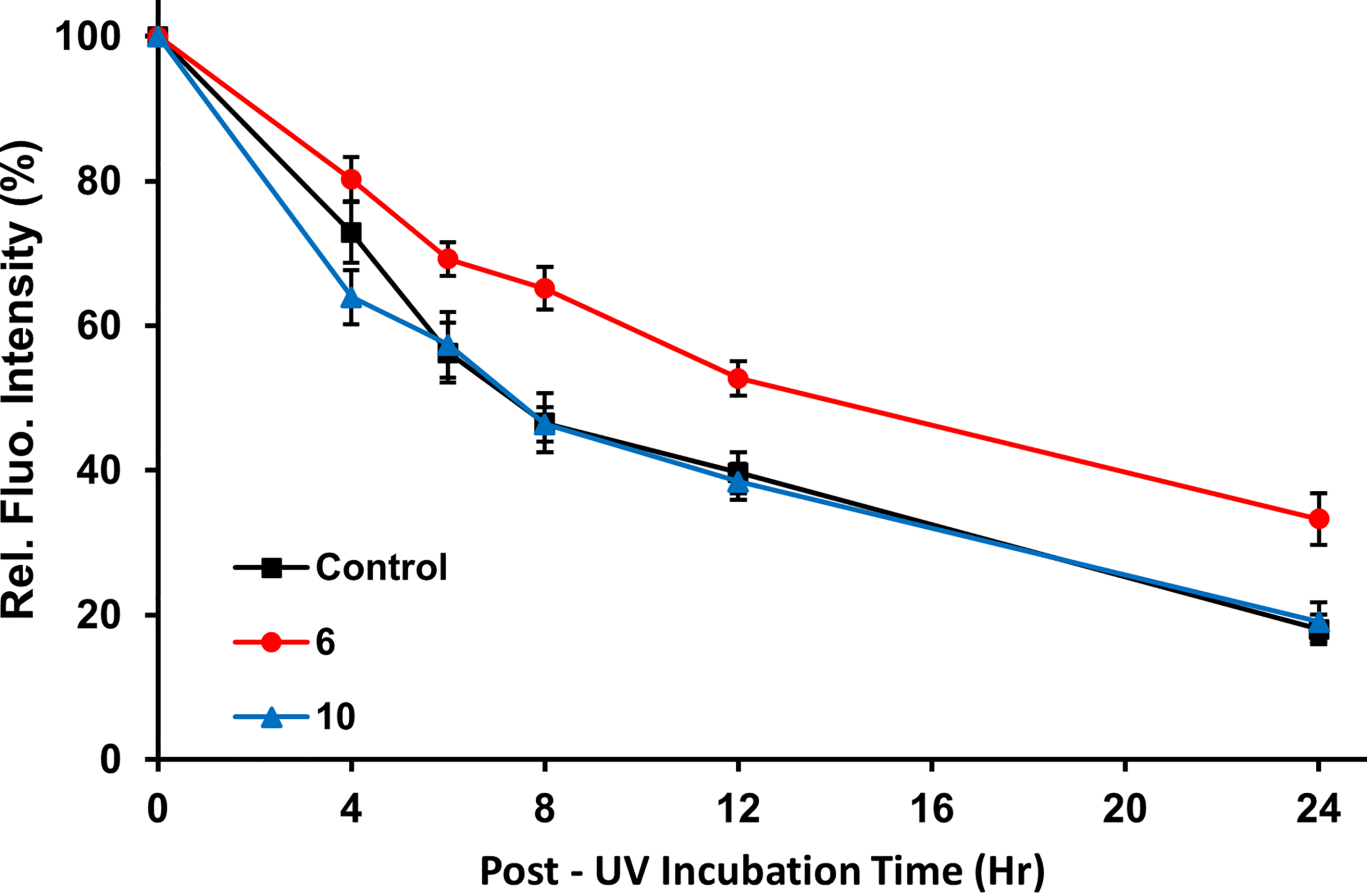
Inhibition of cellular NER. Immunofluorescence images were obtained at various time points to monitor the removal of CPDs from UVC-irradiated HCT-116 cells treated with vehicle only (control), compound 10 (2 μM; negative control), or compound 6 (2 μM). Plot shows the normalized fluorescence intensity of the cells based on quantitation of fluorescence from 100 cells randomly selected per time point. Error bars indicate the S.E.M. The measured intensities of cells treated with the active inhibitor, compound 6 were significantly different from the non-inhibitory negative control, compound 10, at time points from 4 to 24 h post-irradiation (p < 0.005, Student’s t-test) indicating that compound 6 slows the removal of CPD.

Immunofluorescent detection of cyclobutane pyrimidine dimers (CPD) generated in the DNA of HCT-116 cells after exposure to UVC radiation was used to measure NER. Our observations that approximately 80% of CPD were removed 24 hours post irradiation in the absence of any inhibitors mirrors previous observations (70, 79). Addition of 2 μM 10 as a negative control showed no significant effect on CPD removal (**Figure 4**). Addition of 2 μM 6 inhibited the removal of CPD to approximately 67% (**Figure 4**), showing that 6 caused significant inhibition of NER in a cell-based setting.

### Inhibition of the Heterodimerization of ERCC1 and XPF

**Figure 5:**
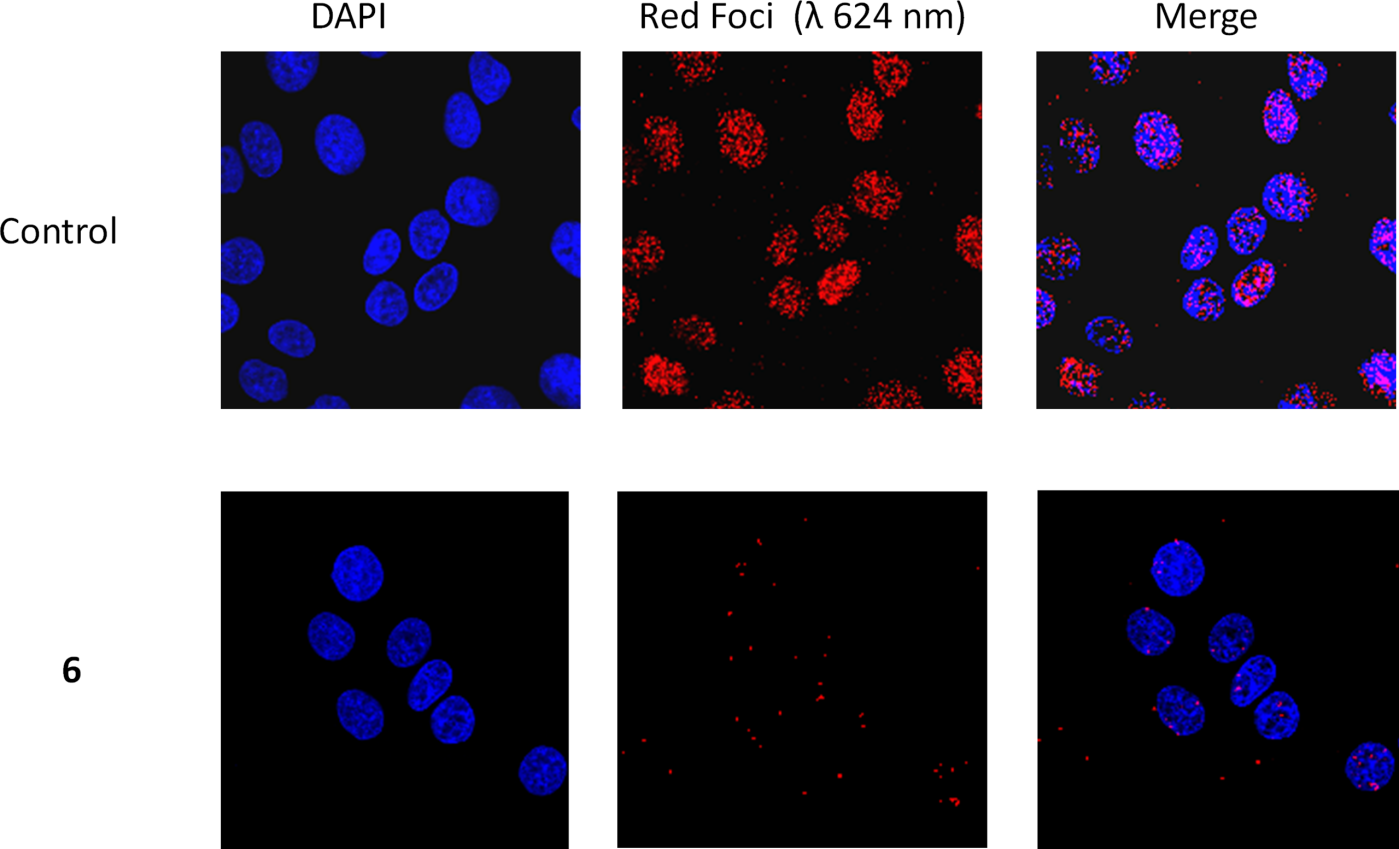
Representative PLA images of A549 cells exposed to 2 µM compound 6 or the equivalent amount of DMSO vehicle (1 µl/ml, Control). Images were obtained at 40X magnification. ERCC1-XPF complexes appear as red dots, and cellular nuclei are shown in blue after DAPI staining.

To confirm that compound **6** was able to disrupt the interaction between subunits in the ERCC1-XPF heterodimer in cells, we performed PLA using A549 lung cancer cells exposed to 2 µM compound 6 or DMSO vehicle for 24 h. (**Figure 5**). In the presence of vehicle (0.2% DMSO) alone we observed an average of 75.7 ± 15.4 foci per cell whereas in the presence of compound 6 only an average of 3.1 ± 2.6 foci per cell were detected. The few red dots observed outside of the nuclei may be derived from disrupted nuclear membrane integrity or compromised membrane permeability which may occur during the experiment at multiple points (i.e. cell culture, washing of cells, trypsinization, cell manipulation, etc.). They may also arise from heterodimers that have not been translocated to the nucleus after protein synthesis. To rule out the possibility that compound 6 caused a reduction in the levels of ERCC1 or XPF, we compared the levels of the proteins in untreated cells and cells treated with compounds 4 and 6 and observed no significant differences (**Supplementary Figure 2**). These data extend the inhibitory observations of compound 6 on ERCC1-XPF from a cell free model to intact cells.

### Sensitization of HCT-116 Cells to DNA Damaging Agents

HCT-116 cells were incubated with media containing a non-toxic concentration of 6 or 10 (**Supplementary Figure 3**) prior to exposure to the DNA damaging agents.

#### Sensitization to UVC Radiation

**Figure 6:**
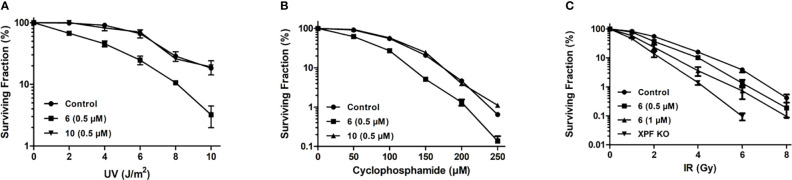
Sensitization of HCT-116 cells to DNA damaging agents as determined by clonogenic survival assay. **(A)** Survival of HCT-116 cells exposed to increasing doses of 254 nm UV radiation and treated with 0.5 μM compound 6 or 10 **(B)** Survival of HCT-116 cells exposed to increasing doses of cyclophosphamide and treated with 0.5 μM compound 6 or 10. **(C)** Survival of HCT-116 cells exposed to increasing doses of ionizing radiation and treated with 0.5 and 1 μM compound 6. The radiosensitivity of HCT-116 XPF knockout cells (70) is provided for comparison.

Compound 6 was tested for its ability to sensitize HCT-116 cells to UVC radiation. At the non-toxic concentration of 0.5 μM, 6 sensitized cells to UVC radiation (**Figure 6A**). Compound 10 was used as a negative control at the same concentrations and showed no sensitization of HCT-116 to UVC radiation, as expected (**Figure 6A**).

#### Sensitization to Cyclophosphamide

Compounds 6 and 10 (negative control) were tested for their ability to sensitize HCT-116 cells to the DNA crosslinking agent cyclophosphamide (**Figure 6B**). HCT-116 cells were treated with 0.5 μM 6 or 1.0 μM 10 before being exposed to increasing concentrations of cyclophosphamide. Compound 6 sensitized the cells to cyclophosphamide, with almost no cells surviving at 250 μM cyclophosphamide. Compound 10 showed no significant sensitization of HCT-116 cells to cyclophosphamide.

#### Sensitization to Ionizing Radiation

HCT-116 cells were exposed to increasing intensities of ionizing radiation to establish the baseline sensitivity of HCT-116 cells to ionizing radiation. HCT-116 derived XPF knockout cells were exposed to increasing intensities of ionizing radiation to provide a theoretical maximal sensitization of HCT-116 cells to ionizing radiation. Addition of 0.5 and 1 μM 6 sensitized HCT-116 cells in a dose-dependent manner (**Figure 6C**).

### ADME

Further investigation of the pharmacokinetics of compound 6 was carried out by WuXi AppTec (Shanghai) Co by performing absorption, distribution, metabolism, and excretion (ADME) measurements. The screening included distribution coefficient (log D), solubility, cell permeability, serum protein binding, CYP inhibition, and microsomal and hepatocyte stability (**Table 2**). The compound has a log D at pH 7.4 of 3.95 and a low to moderate metabolic stability as determined by both liver microsome and hepatocyte assays. The results show that 6 is a moderate inhibitor of CYP1A2, CYP2D6, and CYP 3A4-M and weak inhibitor of CYP2C9 and CYP2C19.

**Table 2:** Pharmacokinetic profile of compounds **4** and **6**.

Screening Test	Compound	Results				
**LogD at pH 7.4**	Compound 6	3.95				
	Compound 4	2.86				
**Metabolic stability in human liver microsomes**	Compound 6	376.2 (mL/min/kg)				
	Compound 4	44.0 (mL/min/kg)				
**Metabolic stability in cryopreserved human hepatocytes**	Compound 6	33.5 (mL/min/kg), T^1/2^ 114.9 (min)				
	Compound 4	48.8 (mL/min/kg), T^1/2^ 79.0 (min)				
**Permeability** **(Efflux Ratio)**	Compound 6	27.51				
	Compound 4	8.92				
**Inhibition of cytochrome P450** **(IC_50_)**	Compound 6	CYP1A2	CYP2C9	CYP2C19	CYP2D6	CYP3A4-M
		6.51 µM	33.4 µM	10.8 µM	2.92 µM	1.9 µM
	Compound 4	CYP1A2	CYP2C9	CYP2C19	CYP2D6	CYP3A4-M
		6.40 µM	>50 µM	>50 µM	16.0 µM	37.1 µM

## Discussion

Our previous studies (70) made it clear that significant improvements in IC_50_ could be realized by modifying the piperazine side chain of 1, although it was originally unclear if the improvement of IC_50_ could be attributed to simply increased steric bulk in that area, the addition of a heteroatom, or any other interactions with the ERCC1-XPF interface. To interrogate the key interactions and to investigate what further changes could be made to this site we carried out an MOE pharmacaphore-assisted docking experiment to identify compounds with modifications in this area that could improve upon the binding observed with 4. The top 32 scoring compounds were selected based on their GBVI/WSA score and MM/GBSA scores were calculated; from these MM/GBSA scores (top ten in **Table 1**) there was a standout performer in compound 6 with significantly lower binding energy than any of the other hits.

We then set out to synthesize the top ten performing inhibitors for *in vitro* testing. Of the top ten inhibitors from the *in silico* screen six compounds were successfully synthesized, including the top performing compound 6. These six compounds were tested in an *in vitro* endonuclease assay to assess their ability to inhibit the endonuclease activity of ERCC1-XPF (**Figure 3A**); surprisingly, compounds 8, 10, 11, 12, and 15 were unable to improve on the inhibition shown by the parent compound 4 despite having similar or lower calculated GBSA scores. However, the top scoring compound 6 did show a modest increase in inhibition over the parent compound 4, and a lowering of calculated IC_50_ from 0.33 μM for 4 (70) to 0.17 μM for 6 (**Figure 3A**).

To first test if the increased inhibition of endonuclease activity would translate to activity in cells we tested the ability of 6 to inhibit the removal of UV induced CPD in cells. As expected, compound 6 was able to slow the removal of CPD by ERCC1-XPF at a concentration of 2 μM, whereas our negative control compound 10 at the same concentration was unable to slow the removal of CPD *vs* the control (**Figure 4**). Interestingly, the inhibition of the removal of CPD was slightly less effective using 2 μM concentration of compound 6 than the same concentration of compound 4 (67% and 60% (70) CPD removal after 24 hours, respectively). Despite the slightly reduced inhibition of CPD removal as compared to compound 4, the inhibition of CPD removal observed with compound 6 provided strong evidence that 6 inhibited cellular NER and would likely be able to sensitize cells to DNA damaging chemotherapies with similar efficacy as that observed with compound 4.

Before carrying out sensitization studies we attempted to gather evidence that the inhibition of cellular NER was a result of compound 6 binding to ERCC1-XPF and inhibiting the heterodimerization. Fluorescence spectroscopy revealed a dose dependent quenching of the intrinsic fluorescence of ERCC1-XPF tryptophan residues (**Figure 3C**) providing strong evidence of compound 6 binding directly to ERCC1-XPF. However, the calculated K_d_ of 140 nM from the intrinsic fluorescence spectroscopy indicates slightly weaker binding of compound 6 to ERCC1-XPF than the K_d_ of 100 nM calculated for compound 4 (70), which runs contrary to what was expected from the computational results. The confounding results from the computational screening and the binding affinity means that we may need to re-evaluate our calculated mode of binding in future work to build a more accurate computational model. Nonetheless, the calculated K_d_, as well as inhibition of endonuclease activity, and inhibition of cellular NER provided strong evidence that compound 6 could be an effective inhibitor of ERCC1-XPF.

The PLA results also provided further evidence that compound 6 inhibits DNA repair by inhibiting the heterodimerization of ERCC1 and XPF. In the A549 cells there is clear interaction between ERCC1 and XPF at the site of DAPI stained DNA (**Figure 5**), with an average of 76 foci per cell. However, when media containing 2 μM concentration of compound 6 is added the number of foci per cell drops to an average of 3 per cell, showing a clear disruption of ERCC1-XPF heterodimerization (**Figure 5**). This result is in keeping with the PLA results previously observed for 1, 4, and 5 (73), where this series of inhibitors showed clear inhibition of the interaction between ERCC1 and XPF when A549 cells were treated with 20 μM cisplatin and 1 μM concentrations of the inhibitors. However, only compound 5 showed any significant disruption of ERCC1-XPF heterodimerization on A549 not treated with cisplatin in those experiments. At this juncture it is difficult to make direct comparisons between these results and those obtained by Ciniero et al. (73) due to the large difference in the foci per cell of the A549 control cells and the different concentrations of inhibitors used between the experiments (2 μM and 1 μM, respectively). Despite the differences in details of experimental methodology, it is clear that compound 6 has a profound impact on the heterodimerization of ERCC1 and XPF, which provides further evidence for our hypothesized mode of inhibition.

We then tested the ability of compound 6 to sensitize cells to DNA damaging agents. Since compound 6 had already been shown to inhibit removal of UV-induced CPD, sensitization of HCT-116 cells to UVC radiation was the obvious starting point. At a concentration of 0.5 μM, compound 6 sensitized HCT-116 cells to UVC radiation as expected, with the negative control 10 showing no sensitization (**Figure 6A**). For example, at 4 J/m^2^ 91% of the untreated cells survived but <50% of cells treated with 0.5 μM compound 6 survived. By comparison, 1.0 μM compound 4 reduced survival of HCT-116 cells to **~** 50% under the same conditions (70). This sensitization of cells to UVC radiation is a promising sign that HCT-116 cells can be made more sensitive to treatments that cause DNA damage that is repaired by NER, allowing for more effective treatments at lower doses.

Following the successful sensitization of HCT-116 cells to UVC radiation we wished to investigate the sensitization of HCT-116 cells to the DNA crosslinking agent cyclophosphamide. Successful sensitization of cancer cells to cyclophosphamide would imply that 6 inhibited cellular ICL as well as NER, lending credence to the notion that compound 6 was indeed inhibiting NER by inhibiting the endonuclease activity of ERCC1-XPF and not *via* inhibition of another protein involved in NER. Compound 6 sensitized HCT-116 cells to cyclophosphamide, with 10 again showing no sensitization (**Figure 6B**). Notably, at the lowest dosage of cyclophosphamide (50 μM) only 62% of the HCT-116 cells treated with 0.5 μM of 6 survived, in contrast to the 91.5% that survived when treated with cyclophosphamide alone, and at 250 μM cyclophosphamide there were almost no surviving HCT-116 cells when treated with 0.5 μM compound 6. Elmenoufy and co-workers (70) had previously reported much more modest sensitization of HCT-116 cells to 50 μM concentration of cyclophosphamide (about 75% cell survival) with double the concentration of compound 4 (i.e.,1 μM). This increase in the potentiation of cyclophosphamide activity by compound 6 offers support to the notion that inhibition of ERCC1-XPF could allow lowering of the effective doses of cytotoxic DNA-targeting drugs such as cyclophosphamide, which is known to have a plethora of negative side effects (81–87).

Following the outcome of compound 6 in sensitizing HCT-116 cells to DNA therapies that cause DNA damage that is repaired by NER and ICL, we turned our attention to cells damaged by cobalt-60 γ-rays. Previous studies have indicated that ERCC1-XPF deficient mammalian cells display enhanced sensitivity to ionizing radiation and reduced DSB repair (24, 88, 89). In agreement with these earlier findings, our CRISPR-mediated XPF knockout cells were shown to be significantly more sensitive to ionizing radiation than the control HCT-116 cells (**Figure 6C**). We therefore treated HCT-116 cells with 0.5 and 1 μM concentrations of compound 6 before exposure to increasing doses of ionizing radiation. Compound 6 sensitized HCT-116 cells to ionizing radiation in a dose dependent manner (**Figure 6C**). Importantly, sensitization was observed even at the typical clinical dose of 2 Gy (90) with both 0.5 and 1 μM concentrations of 6. Our data clearly indicate a potential role for targeting ERCC1-XPF to enhance radiotherapy.

The ADME results (**Table 2**) when compared with the previous ADME screening of parent hit compound 1, and first and second generation compounds 4 (70) and 5 (71) indicated fairly similar responses to compound 4. However, two differences in the ADME data between compounds 6 and 4 may have a possible bearing on the lower sensitization capacity seen with compound 4 relative compound 6. First, compound 6 inhibits cytochromes 2C19, 2D6 and 3A4-M, much more effectively than compound 4. It is therefore feasible that compound 4 is metabolized more rapidly in HCT116 cells than compound 6. Similarly, if the clearance from human hepatocytes reflects the clearance from HCT116 cells, it is noticeable that compound 4 is cleared more rapidly than compound 6 (T^1/2^ = 79 *vs* 114.9 min), which would imply lower cellular retention of active compound 4 *vs* 6. These possibilities will need to be further explored.

In conclusion, we used a computer aided drug design strategy to identify potential inhibitors of ERCC1-XPF based on the modification of the piperazine side-chain of the previously reported inhibitor 4. Of the compounds screened, compound 6 was the best performing compound by a wide margin. Six of the 10 best performing compounds were synthesized and subjected to *in vitro* testing to inhibit the endonuclease activity of ERCC1-XPF. Compound 6 was the best performing of the synthesized inhibitors *in vitro* and was subsequently shown to inhibit NER in cells. Our binding studies and PLA provided further evidence that the observed inhibition was due to the inhibition of the heterodimerization of ERCC1 and XPF. Compound 6 was then shown to sensitize HCT-116 cells to UVC radiation, cyclophosphamide, and ionizing radiation; proving that it is a promising candidate to be used alongside existing DNA damaging therapies. Furthermore, we have found that variation of the piperazine side-chain is well tolerated and does not interfere with what we believe to be the key binding between the binding pocket in the HhH2 domain of XPF and the aminophenol substituted acridine moieties found in 1, 4, 5, and 6. This discovery allows for further functionalization at this site and the potential for the introduction of moieties to improve the binding affinity and pharmacokinetic properties of this series of inhibitors.

## Data Availability Statement

The datasets presented in this study can be found in online repositories. The names of the repository/repositories and accession number(s) can be found in the article/**Supplementary Material**.

## Author Contributions

CW synthesized and characterized all the compounds. DJ expressed and purified ERCC1-XPF protein, carried out the *in vitro* endonuclease assays, and the PLA. JD wrote the manuscript. FG carried out the computational studies. FK-B and XY carried out cell culturing, UV dimer repair assays, and clonogenic survival assays. RM conducted the binding affinity study. YY provided the XPF knockout cells. KB and JT supervised FG in designing the computational studies. AE contributed to compound design and interpretation of assay results. MW supervised XY and co-wrote and edited the manuscript. FW supervised CW, JD and AE and co-wrote and edited the manuscript. All authors contributed to the article and approved the submitted version.

## Funding

This work was supported by grants funded by the Alberta Cancer Foundation Transformative Program Project (26603) and the Alberta Cure Cancer Foundation. We also acknowledge the support of the Savard Family Lung Research Fund. JD was supported by the La Vie En Rose Scholarship for Fundamental Breast Cancer Research. FG was supported by an Alberta Innovates graduate student scholarship and a Novartis Pharmaceuticals Canada Inc. graduate scholarship. The funder was not involved in the study design, collection, analysis, interpretation of data, the writing of this article or the decision to submit it for publication.

## Conflict of Interest

The authors declare that the research was conducted in the absence of any commercial or financial relationships that could be construed as a potential conflict of interest.

## Publisher’s Note

All claims expressed in this article are solely those of the authors and do not necessarily represent those of their affiliated organizations, or those of the publisher, the editors and the reviewers. Any product that may be evaluated in this article, or claim that may be made by its manufacturer, is not guaranteed or endorsed by the publisher.
