# Supplementary material for: Modulation of ERCC1-XPF Heterodimerization Inhibition via Structural Modification of Small Molecule Inhibitor Side-Chains
Source: Front Oncol. 2022 Mar 17;12:819172. doi: 10.3389/fonc.2022.819172 (PMC8968952; doi:10.3389/fonc.2022.819172)
Supplement: Supplementary file 1 [file DataSheet_1.pdf]

Supplementary Material

1 Inhibition of ERCC1-XPF Endonuclease Activity

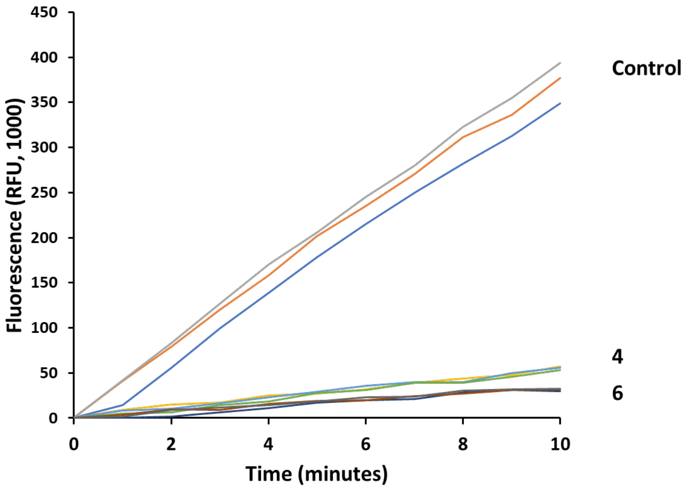

Supplementary Figure 1. ERCC1-XPF mediated cleavage of the stem-loop DNA substrate, in which the FAM signal is quenched, releases the fluorescently tagged octanucleotide. Triplicate measurements of the control (DMSO) and the effect of 10 µM of compounds **4** and **6** on the incision activity is shown.

2 PLA Control Experiments

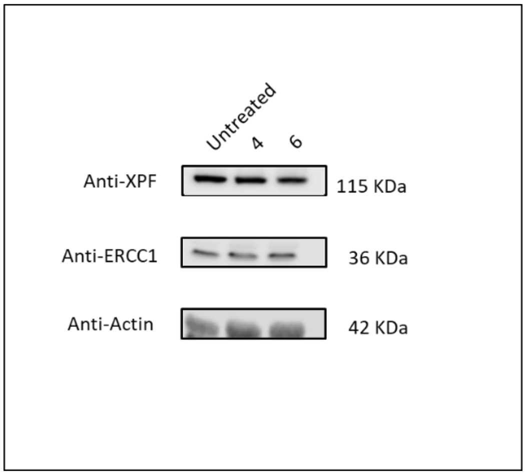

Supplementary Figure 2. A549 cells were seeded in 6-well culture dishes (Thermo Fishes Scientific) at a density of 0.3 X 10^6 cells/well in a final volume of 3 ml. The cells were left to adhere for 24 h before adding 2 µM of compound **4** or compound **6**. The chambers were incubated for 24 h and then washed with PBS and the cells were solubilized with RIPA buffer in the presence of protease inhibitor cocktail (Cell Signaling Technology) plus 2 mM EDTA. Proteins were dissolved in sample buffer and separated ina 4-20 % polyacrylamide gel and electrophoretically transferred to

nitrocellulose. Proteins were visualized using ERCC1 (A73368-100, 1:1000; EpiGentek), XPF (LS-C173159, 1:1000; LifeSpan BioSciences, Seattle, WA) and B-Actin (sc-47778, 1:1000; Santa Cruz) primary antibodies followed by secondary anti-mouse or anti-rabbit secondary HRP-conjugated secondary antibodies. ECL chemiluminescent substrate (Promega) was used to detect the proteins.

3 Cytotoxicity Analysis

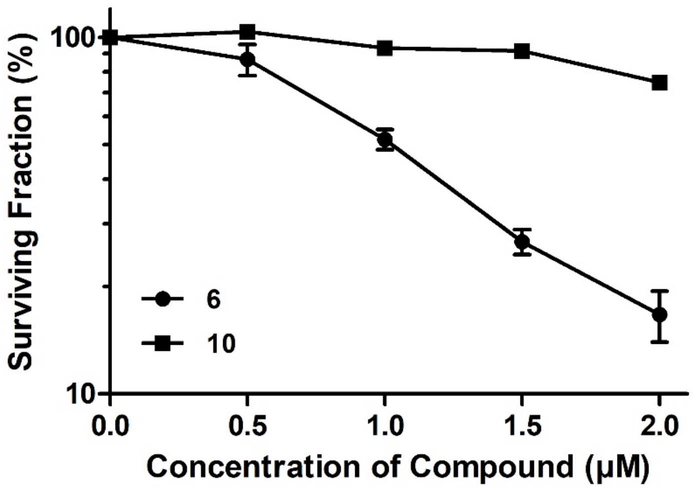

Supplementary Figure 3. Cytotoxicity assay using colony forming assay

4 HCT-116 Derived XPF Knockout Cells

For CRISPR deletion of XPF from HCT-116 cells, the pSpCas9(BB)-2A-GFP (pX458) vector (Addgene plasmid # 48138) was used. The short guide sequence to target XPF exon one was: 5'-GGGCTAGTAGTGTGCGCCCG- 3' and target XPF exon eight was: 5'-CTATATCACTCTTGGAGCGG-3'. All short guide sequences oligonucleotides were synthesized, annealed and cloned into pX458 vector and were confirmed by DNA sequencing at DNA laboratory, University of Calgary. The DNA plasmid (pX458 containing XPF short guide RNA sequence, 5 µg) was transfected into HCT-116 cells using Lipofectamine 2000 (Invitrogen) according to the manufacturer's instructions. Forty-eight hours after transfection, cells were harvested and genomic DNA was isolated using the KAPA Express Extract Kit (Kapa Biosystems) according to the manufacturer's instructions. Genomic DNA fragments of XPF around the short guide RNA site were amplified by PCR. The primers used to amplify the genomic region of XPF exon one were: Forward: 5'- CACTAGGAGTCGGCTTCCTT-3', Reverse: 5'- TCTCTGTGTCATCGCGTAGT-3' and exon eight were: Forward: 5'- TCGGGTGAAGGAATAAGGGG-3', Reverse: 5'-ATTTCTTCCGGGCAGCTTTC-3'. PCR products were used for Surveyor nuclease mutation detection assays using the SURVEYOR Mutation Detection Kit (IDT) according to manufacturer's protocol. After transfection, GFP containing cells were sorted into 96-well plates by flow cytometry, at the Flow Cytometry Facility, University of Calgary. Single cells were then expanded for further analysis (i.e., Western blot and DNA sequencing). All CRISPR gene knockout clones were screened by Western blot using anti-XPF antibody (MA12060, ThermoFisher). The membrane was also probed with Mre11 antibody (Novus) as a loading control. For DNA sequencing confirmation of

XPF knockout clones, DNA fragments of XPF knockout clones around the short guide RNA site were amplified by PCR. PCR products were subcloned into pEGFP-C2 vector (Clontech) and plasmid DNA from individual clones were sent for Sanger DNA sequencing at DNA laboratory, University of Calgary to confirm all indels.

Allele 1

```
XPF-E1          ATGGAGTCAGGGCAGCCGGCTCGACGGATTGCCATGGCGCCGCTGCTGGAGTACGAGC
1-2-1a          ATGGAGTCAGGGCAGCCGGCTCGACGGATTGCCATGGCGCCGCTGCTGGAGTACGAGC
                **********************************************************

XPF-E1          GACAGCTGGTGCTGGAACTGCTCGACACTGACGGGCTAGTAGTGTGCGC-----------
1-2-1a          GACAGCTGGTGCTGGAACTGCTCGACACTGACGGGCTAGTAGTGTGCGCGCGGCCTCAGT
                *************************************************

XPF-E1          -------------------------------------------------CCGCGGGCTCG
1-2-1a          GAGCGAGCGAGCGCGCAGCTGCCTGCAGGGGCGCCTGATGCGGTATTTTCCGCGGGCTCG
                                                                 ***********

XPF-E1          GCGCGGACCGGCTCCTCTACCACTTTCTCCAGCTGCACTGCCACCCAGCCTGCCTGGTGC
1-2-1a          GCGCGGACCGGCTCCTCTACCACTTTCTCCAGCTGCACTGCCACCCAGCCTGCCTGGTGC
                ************************************************************


XPF-E1          TGGTGCTCAACACGCAGCCGGCCGAGGAG
1-2-1a          TGGTGCTCAACACGCAGCCGGCCGGGGAG
                ************************ ****
```

Allele 2

```
XPF-E1          ATGGAGTCAGGGCAGCCGGCTCGACGGATTGCCATGGCGCCGCTGCTGGAGTACGAGCGA
1-2-1b          ATGGAGTCAGGGCAGCCGGCTCGACGGATTGCCATGGCGCCGCTGCTGGAGTACGAGCGA
                ************************************************************

XPF-E1          CAGCTGGTGCTGGAACTGCTCGACACTGACGGGCTAGTAGTGTGCGCCCGCGGGCTCGGC
1-2-1b          CAGCTGGTGCTGGAACTGCTCGACACTGACGGGCTAGTAGTGTGCGCC-GCGGGCTCGGC
                ************************************************ ***********

XPF-E1          GCGGACCGGCTCCTCTACCACTTTCTCCAGCTGCACTGCCACCCAGCCTGCCTGGTGCTG
1-2-1b          GCGGACCGGCTCCTCTACCACTTTCTCCAGCTGCACTGCCACCCAGCCTGCCTGGTGCTG
                ************************************************************


XPF-E1          GTGCTCAACACGCAGCCGGCCGAGGAG
1-2-1b          GTGCTCAACACGCAGCCGGCCGAGGAG
                ***************************
```

Supplementary Figure 4. Sequence analysis of the HCT116 XPF-/- cell clone (1-2-1a). 60 nucleotides were inserted and one nucleotide was mutated in Allele 1; one nucleotide was deleted in Allele 2. The targeted sequences in the WT exon 1 allele (XPF-E1) corresponding to the guide RNA is underlined.

5 Compound Synthetic Procedures and Characterization

General Information

Reactions were carried out in flame or oven dried glassware under a positive nitrogen atmosphere unless otherwise stated. Transfer of anhydrous solvents and reagents was accomplished with oven-dried syringes or cannulae. Solvents and some reagents were distilled before use. Commercially available reagents were used without further purification. Thin layer chromatography was performed on glass plates precoated with 0.25 mm silica gel. Flash chromatography was performed on 230-400 mesh silica gel with the indicated eluents. Nuclear magnetic resonance (NMR) spectra were recorded in indicated deuterated solvents and are reported in ppm in the presence of TMS as internal standard and coupling constants (*J*) are reported in hertz (Hz). The spectra are referenced to residual solvent peaks: chloroform-*d* (7.26 ppm, ^{1}H; 77.26 ppm, ^{13}C), DMSO-*d*6 (2.50 ppm, ^{1}H; 39.51 ppm, ^{13}C), acetone-*d*6 (2.05 ppm, ^{1}H; 206.68 and 29.92 ppm, ^{13}C) and methanol-*d4* (3.31 157 ppm, ^{1}H; 49.00 ppm, ^{13}C). Proton nuclear magnetic spectra (^{1}H NMR) and carbon nuclear magnetic resonance spectra (^{13}C NMR) were recorded at 500/400 and 125/100 MHz respectively. Mass spectra were recorded by using electrospray ionization (ESI).

4-((6-Chloro-2-methoxyacridin-9-yl)amino)-2-((4-(2-(diisopropylamino)ethyl)piperazin-1-yl)methyl)phenol (6)

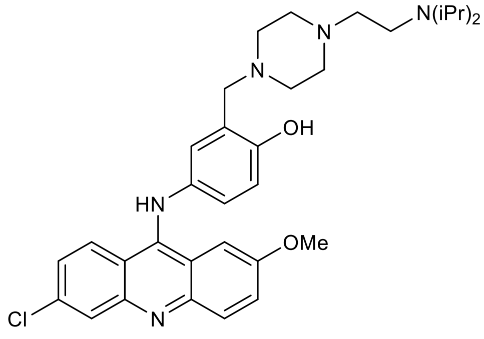

6

Compound **20** (93 mg, 0.19 mmol) was dissolved in DCM (2.5 ml). Diisopropylamine (30 µl, 21 mg, 0.21 mmol) and acetic acid (3 µl, 3 mg, 0.06 mmol) were added and allowed to stir for 30 minutes before addition of $NaBH(OAc)_3$ (48 mg, 0.23 mmol). The reaction mixture was allowed to stir at room temperature overnight. After the completion of the reaction, as indicated by TLC, the reaction was quenched with satd. $NaHCO_3$ solution, then 1 M NaOH solution. The reaction mixture was then adjusted to pH = 8-9 by addition of 1M HCl. Extraction with DCM and drying of organic extracts with Na_2SO_4, filtration and removal of solvent under reduced pressure afforded the crude product. The compound was purified by column chromatography and recrystallization with MeCN produced a brown solid (84 mg, 76%). ^{1}H NMR (400 MHz, Chloroform-*d*) δ 8.14 (d, *J* = 2.1 Hz, 1H), 8.04 (d, *J* = 9.4 Hz, 1H), 7.89 (d, *J* = 9.1 Hz, 1H), 7.41 (dd, *J* = 9.5, 2.7 Hz, 1H), 7.06 (d, *J* = 2.7 Hz, 1H), 6.89 – 6.81 (m, 1H), 6.76 (d, *J* = 8.5 Hz, 1H), 6.54 (s, 1H), 6.44 (s, 1H), 3.74 (s, 3H), 3.57 (s, 2H), 2.98 (p, *J* = 6.5 Hz, 2H), 2.54 (dd, *J* = 10.0, 6.0 Hz, 2H), 2.39 (dd, *J* = 9.8, 6.2 Hz, 2H), 1.55 (broad s, 8H), 0.99 (d, *J* = 6.5 Hz, 12H); ^{13}C NMR (175 MHz, Chloroform-*d*) δ 156.3, 153.5, 148.4, 147.5, 143.0, 136.5, 134.9, 131.7, 128.5, 125.5, 125.3, 124.4, 122.0, 120.1, 119.8, 117.9, 116.9, 99.6, 61.3, 60.6, 55.4, 53.5, 53.4, 52.5, 49.3, 43.0, 29.7, 20.8, 1.0. HRMS (ESI) *m/z* calculated for $C_{33}H_{41}ClN_5O_2$ $[M-H]^-$ 574.2954; found 574.2945.

4-((6-Chloro-2-methoxyacridin-9-yl)amino)-2-((4-(3-((2,3-dihydro-1H-inden-2-yl)(methyl)amino)propyl)piperazin-1-yl)methyl)phenol (8)

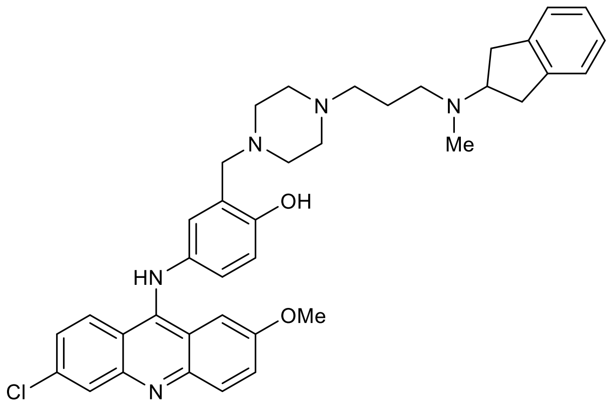

8

Compound **25** (142 mg, 0.28 mmol) was dissolved in DCM (5 ml). *N*-methyl-2,3-dihydro-1H-inden-2-amine (46 mg, 0.31 mmol) and acetic acid (5 µl, 5 mg, 0.08 mmol) were added and allowed to stir for 30 minutes before addition of $NaBH(OAc)_3$ (72 mg, 0.34 mmol). The reaction mixture was allowed to stir at room temperature overnight. After the completion of the reaction, as indicated by TLC, the reaction was quenched with satd. $NaHCO_3$ solution, then 1 M NaOH solution. The reaction mixture was then adjusted to pH = 8-9 by addition of 1M HCl. Extraction with DCM and drying of organic extracts with Na_2SO_4, filtration and removal of solvent under reduced pressure afforded the crude product. The compound was purified by column chromatography and recrystallization with MeCN produced a brown oil (23 mg, 16%). HRMS (ESI) *m/z* calculated for $C_{38}H_{43}ClN_5O_2$ $[M+H]^+$ 636.3100; found 636.3096

2-((4-(2-(Benzylamino)ethyl)piperazin-1-yl)methyl)-4-((6-chloro-2-methoxyacridin-9-yl)amino)phenol (10)

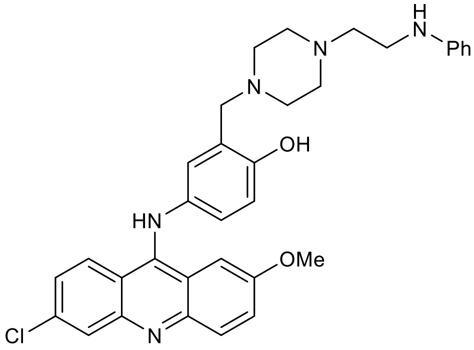

10

Compound **20** (77 mg, 0.16 mmol) was dissolved in DCM (2 ml). Benzylamine (16 µl, 16 mg, 0.21 mmol) and acetic acid (3 µl, 3 mg, 0.06 mmol) were added and allowed to stir for 30 minutes before addition of $NaBH(OAc)_3$ (40 mg, 0.19 mmol). The reaction mixture was allowed to stir at room temperature for 2 days. After the completion of the reaction, as indicated by TLC, the reaction was quenched with satd. $NaHCO_3$ solution, then 1 M NaOH solution. The reaction mixture was then adjusted to pH = 8-9 by addition of 1M HCl. Extraction with DCM and drying of organic extracts with Na_2SO_4, filtration and removal of solvent under reduced pressure afforded the crude product. The compound was purified by column chromatography and recrystallization with MeCN produced an orange solid (41 mg, 49%). ^1H NMR (500 MHz, Chloroform-*d*) δ 8.15 (s, 1H), 8.05 (d, *J* = 9.3

Hz, 1H), 7.91 (d, *J* = 9.3 Hz, 1H), 7.42 (d, *J* = 9.3 Hz, 1H), 7.19 (dd, *J* = 8.6, 7.4 Hz, 2H), 7.07 (s, 1H), 6.85 (dd, *J* = 8.5, 2.7 Hz, 1H), 6.78 (d, *J* = 8.6 Hz, 1H), 6.75 – 6.69 (m, 1H), 6.63 (dd, *J* = 8.6, 1.1 Hz, 2H), 6.55 (d, *J* = 2.7 Hz, 1H), 6.45 (s, 1H), 4.23 (s, 1H), 3.75 (s, 3H), 3.60 (s, 2H), 3.50 (s, 2H), 3.17 (t, *J* = 5.9 Hz, 2H), 2.98 – 2.32 (m, 10H).; ^{13}C NMR (125 MHz, Chloroform-*d*) δ 156.4, 153.5, 148.5, 136.8, 135.1, 129.4, 125.3, 124.7, 122.1, 120.3, 119.9, 118.0, 117.6, 117.0, 113.0, 99.9, 61.4, 56.6, 55.5, 53.6, 52.8, 52.6, 40.4, 30.1, 29.8; HRMS (ESI) *m/z* calculated for $C_{33}H_{35}ClN_5O_2$ $[M+H]^+$ 568.2474; found 568.2471.

4-((6-Chloro-2-methoxyacridin-9-yl)amino)-2-((4-(2-((thiophen-2-ylmethyl)amino)ethyl)piperazin-1-yl)methyl)phenol (11)

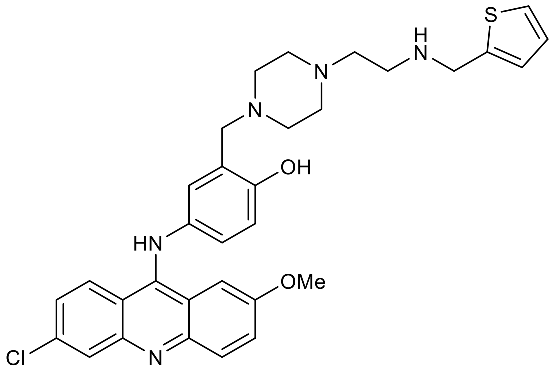

11

Compound **20** (93 mg, 0.19 mmol) was dissolved in DCM (2.5 ml). 2-Thiophenemethylamine (21 µl, 24 mg, 0.38 mmol) and acetic acid (3 µl, 3 mg, 0.06 mmol) were added and allowed to stir for 30 minutes before addition of $NaBH(OAc)_3$ (48 mg, 0.23 mmol). The reaction mixture was allowed to stir at room temperature for 3 days. After the completion of the reaction, as indicated by TLC, the reaction was quenched with satd. $NaHCO_3$ solution, then 1 M NaOH solution. The reaction mixture was then adjusted to pH = 8-9 by addition of 1M HCl. Extraction with DCM and drying of organic extracts with Na_2SO_4, filtration and removal of solvent under reduced pressure afforded the crude product. The compound was purified by column chromatography and recrystallization with MeCN produced a yellow solid (75 mg, 67%). ^{1}H NMR (500 MHz, Chloroform-*d*) δ 1H NMR (500 MHz, Chloroform-d) δ 8.15 (s, 1H), 8.05 (d, *J* = 9.2 Hz, 1H), 7.91 (d, *J* = 9.2 Hz, 1H), 7.42 (d, *J* = 9.4 Hz, 1H), 7.21 (dd, *J* = 5.0, 1.2 Hz, 1H), 7.07 (s, 1H), 6.95 (dd, *J* = 5.0, 3.4 Hz, 1H), 6.93 – 6.91 (m, 1H), 6.85 (dd, *J* = 8.4, 2.1 Hz, 1H), 6.78 (d, *J* = 8.5 Hz, 1H), 6.55 (d, *J* = 2.7 Hz, 1H), 6.45 (s, 1H), 4.01 (s, 2H), 3.75 (s, 3H), 3.58 (s, 2H), 3.50 (s, 2H), 2.78 – 2.33 (m, 12H); HRMS (ESI) *m/z* calculated for $C_{32}H_{35}ClN_5O_2S$ $[M+H]^+$ 588.2195; found 588.2191.

3-(4-(5-((6-Chloro-2-methoxyacridin-9-yl)amino)-2-hydroxybenzyl)piperazin-1-yl)-N,2,2-trimethylpropanamide (12)

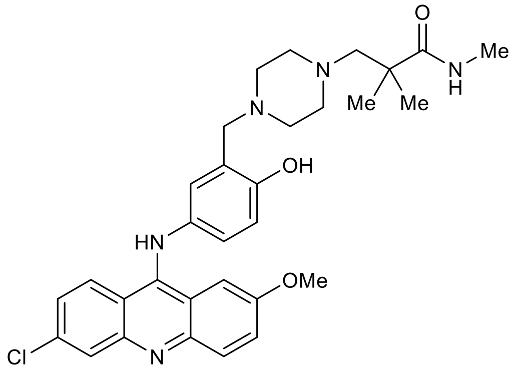

12

6,9-Dichloro-2-methoxyacridine (68 mg, 0.21 mmol) and **32** (59 mg, 0.21 mmol) were dissolved in EtOH (4.5 ml), 5 drops concentrated HCl were added, and the mixture was heated at reflux overnight. The reaction was quenched with saturated $NaHCO_3$ solution and extracted with DCM. The combined organic extracts were dried with $MgSO_4$, filtered, and the solvent evaporated under reduced pressure to afford the crude product as a brown semisolid. The compound was purified by column chromatography, recrystallization from hexanes/DCM, and recrystallization from MeCN (29 mg, 24%). ^{1}H NMR (400 MHz, Chloroform-*d*) δ 8.06 (s, 1H), 7.97 (d, *J* = 9.4 Hz, 1H), 7.86 (d, *J* = 9.3 Hz, 1H), 7.36 (dd, *J* = 9.5, 2.7 Hz, 1H), 7.22 – 7.15 (m, 2H), 7.08 (s, 1H), 6.87 (dd, *J* = 8.6, 2.7 Hz, 1H), 6.77 (d, *J* = 8.6 Hz, 1H), 6.53 (s, 1H), 3.72 (s, 3H), 3.55 (s, 2H), 2.75 (d, *J* = 4.8 Hz, 3H), 2.52 (d, *J* = 81.2 Hz, 10H), 1.13 (s, 6H); ^{13}C NMR (125 MHz, Chloroform-*d*) δ 177.9, 156.3, 153.4, 135.1, 125.3, 124.6, 121.9, 120.3, 119.9, 119.8, 117.0, 66.5, 61.2, 55.4, 54.9, 53.4, 52.9, 42.5, 30.9, 29.7, 26.1, 24.6, 14.1; HRMS (ESI) *m/z* calculated for $C_{31}H_{37}ClN_5O_3$ $[M+H]^+$ 562.2579; found 562.2571.

4-((6-Chloro-2-methoxyacridin-9-yl)amino)-2-((4-(2-methoxyethyl)piperazin-1-yl)methyl)phenol (15)

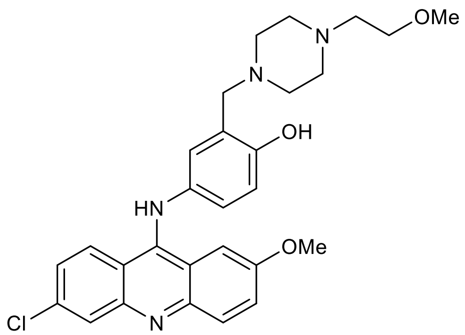

15

6,9-Dichloro-2-methoxyacridine (89 mg, 0.32 mmol) and **35** (85 mg, 0.32 mmol) were dissolved in EtOH (7 ml), 3 drops concentrated HCl were added, and the mixture was heated at reflux overnight. The reaction was quenched with saturated $NaHCO_3$ solution and extracted with DCM. The combined organic extracts were dried with $MgSO_4$, filtered, and the solvent evaporated under reduced pressure to afford the crude product as an orange semisolid. The compound was purified by column chromatography and recrystallization from MeCN (86 mg, 53%). ^{1}H NMR (500 MHz, Chloroform-*d*) δ 8.13 (s, 1H), 8.03 (s, 1H), 7.90 (d, *J* = 9.3 Hz, 1H), 7.40 (d, *J* = 9.4 Hz, 1H), 7.08 (s, 1H), 6.84

(dd, J = 8.5, 2.7 Hz, 1H), 6.77 (d, J = 8.6 Hz, 1H), 6.56 (d, J = 2.7 Hz, 1H), 6.47 (s, 1H), 3.74 (s, 3H), 3.59 (s, 2H), 3.51 (t, J = 5.5 Hz, 2H), 3.36 (s, 3H), 2.60 (t, J = 5.5 Hz, 10H); HRMS (ESI) *m/z* calculated for $C_{28}H_{32}ClN_4O_3$ $[M+H]^+$ 207.2157; found 207.2156.

1-(2,2-Dimethoxyethyl)piperazine (16)

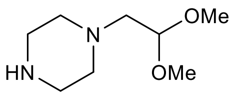

16

Piperizine (2.50 g, 29.0 mmol), 2-bromoacetaldehyde dimethyl acetal (1.7 ml, 2.44 g, 14.5 mmol), and triethylamine (2.0 ml, 1.47 g, 14.5 mmol) were dissolved in EtOH (17 ml) and heated at reflux overnight. After completion of the reaction, as indicated by TLC, the reaction mixture was allowed to cool to room temperature, filtered and the solvent evaporated under reduced pressure. The crude mixture was purified by column chromatography to afford the product (1.66 g, 66%). ^{1}H NMR (500 MHz, Chloroform-*d*) δ 4.53 (t, J = 5.2 Hz, 1H), 3.37 (s, 6H), 2.90 (t, J = 4.9 Hz, 4H), 2.54 – 2.47 (m, 6H), 1.83 (s, 1H); ^{13}C NMR (100 MHz, Chloroform-*d*) δ 102.6, 60.1, 53.8, 53.4; HRMS (ESI) *m/z* calculated for $C_8H_{19}N_2O_2$ $[M+H]^+$ 175.1440; found 175.1440.

2-((4-(2,2-Dimethoxyethyl)piperazin-1-yl)methyl)-4-nitrophenol (17)

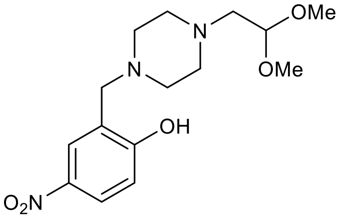

17

2-chloromethyl-4-nitrophenol hydrochloride (1.42 g, 6.35 mmol) and **16** (1.44 g, 8.25 mmol) were dissolved in DCM (52 ml). Triethylamine (1.1 ml, 835 mg, 0.73 mmol) was added in 3 portions and the reaction mixture was allowed to stir at room temperature overnight. After completion of the reaction, as indicated by TLC, the solvent was removed under reduced pressure and the crude mixture was purified by column chromatography to afford the product (2.04 g, 97%). ^{1}H NMR (500 MHz, Chloroform-*d*) δ 8.10 (dd, J = 9.0, 2.8 Hz, 1H), 7.96 (d, J = 2.8 Hz, 1H), 6.86 (d, J = 9.0 Hz, 1H), 4.52 (t, J = 5.2 Hz, 1H), 3.80 (s, 2H), 3.38 (s, 6H), 2.81 – 2.50 (m, 10H); ^{13}C NMR (175 MHz, Chloroform-*d*) δ 164.4, 140.1, 125.4, 124.8, 121.0, 116.5, 102.5, 60.7, 59.4, 53.5, 53.3, 52.3.; HRMS (ESI) *m/z* calculated for $C_{15}H_{24}N_3O_5$ $[M+H]^+$ 326.1710; found 326.1707.

4-Amino-2-((4-(2,2-dimethoxyethyl)piperazin-1-yl)methyl)phenol (18)

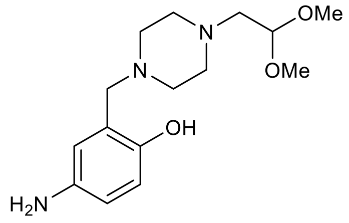

18

MeOH (40 ml) was added to a 3-neck round bottomed flask containing 10% Pd/C (50 mg), before addition of a solution of **17** (1.91 g, 5.87 mmol) in MeOH (50 ml) was added. The reaction vessel was evacuated and backfilled with nitrogen 3 times, then evacuated and backfilled with H_2 3 times before being allowed to stir at room temperature for 7 days. After the completion of the reaction, as indicated by TLC, the reaction mixture was filtered over celite and the solvent removed under reduced pressure. The crude product was purified by column chromatography (1.62 g, 93%).^{1}H NMR (500 MHz, Chloroform-*d*) δ 6.66 (d, *J* = 8.4 Hz, 1H), 6.56 (dd, *J* = 8.4, 2.8 Hz, 1H), 6.39 (d, *J* = 2.8 Hz, 1H), 4.52 (t, *J* = 5.2 Hz, 1H), 3.61 (s, 2H), 3.37 (s, 6H), 2.79 – 2.50 (m, 10H); ^{13}C NMR (100 MHz, Chloroform-*d*) δ 150.4, 138.5, 121.8, 116.6, 116.2, 116.0, 102.4, 61.4, 59.7, 53.7, 53.5, 52.4; HRMS (ESI) *m/z* calculated for $C_{15}H_{26}N_3O_3$ $[M+H]^+$ 296.1970; found 296.1960.

4-((6-Chloro-2-methoxyacridin-9-yl)amino)-2-((4-(2,2-dimethoxyethyl)piperazin-1-yl)methyl)phenol (19)

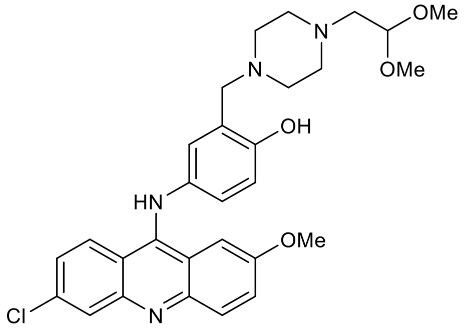

19

6,9-Dichloro-2-methoxyacridine (540 mg, 1.94 mmol) and **18** (573 mg, 1.94 mmol) were dissolved in MeOH (40 ml), 6 drops concentrated HCl were added, and the mixture was heated at reflux overnight. The reaction was quenched with saturated $NaHCO_3$ solution and extracted with DCM. The combined organic extracts were dried with $MgSO_4$, filtered, and the solvent evaporated under reduced pressure to afford the crude product as an orange solid. The compound was purified by column chromatography and recrystallization from MeCN (889 mg, 85%). ^{1}H NMR (500 MHz, Chloroform-*d*) δ 8.13 (s, 1H), 8.03 (s, 1H), 7.89 (d, *J* = 9.3 Hz, 1H), 7.40 (d, *J* = 9.4 Hz, 1H), 7.25 (d, *J* = 8.0 Hz, 1H), 7.07 (s, 1H), 6.84 (dd, *J* = 8.6, 2.7 Hz, 1H), 6.78 (d, *J* = 8.5 Hz, 1H), 6.54 (d, *J* = 2.7 Hz, 1H), 6.51 (s, 1H), 4.52 (t, *J* = 5.2 Hz, 1H), 3.73 (s, 3H), 3.58 (s, 2H), 3.38 (s, 6H), 2.77 – 2.41 (m, 10H); ^{13}C NMR (125 MHz, Chloroform-*d*) δ 156.3, 153.4, 143.1, 136.5, 134.9, 131.7, 128.4, 125.4, 125.2, 124.5, 122.0, 120.1, 119.8, 117.9, 116.9, 116.9, 102.5, 99.6, 61.3, 59.6, 55.3, 53.6, 53.4, 52.4; HRMS (ESI) *m/z* calculated for $C_{29}H_{34}ClN_4O_4$ $[M+H]^+$ 537.2263; found 537.2257.

2-(4-(5-((6-Chloro-2-methoxyacridin-9-yl)amino)-2-hydroxybenzyl)piperazin-1-yl)acetaldehyde (20)

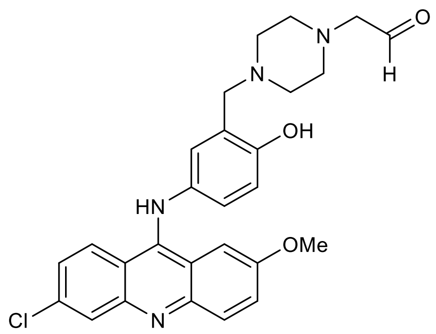

20

Compound **19** (321 mg, 0.60 mmol) was dissolved in DCM (25 ml) and cooled to 0 °C. BBr_3 (1.32 ml, 1.32 mmol) was added dropwise and allowed to stir at 0 °C for 15 minutes. After completion of the reaction, as indicated by TLC, the reaction was carefully quenched by addition of H_2O and the pH adjusted to 8 with a saturated $NaHCO_3$ solution. The reaction mixture was extracted with DCM and the combined organic extracts dried with Na_2SO_4, filtered, and evaporated. The crude product was purified by column chromatography to afford the product as an orange solid (222 mg, 75%). ^{1}H NMR (400 MHz, Chloroform-*d*) δ 9.68 (s, 1H), 8.09 (s, 1H), 7.99 (d, *J* = 9.4 Hz, 1H), 7.87 (d, *J* = 9.2 Hz, 1H), 7.41 – 7.34 (m, 1H), 7.22 (d, *J* = 9.2 Hz, 1H), 7.08 (s, 1H), 6.86 (dd, *J* = 8.6, 2.7 Hz, 1H), 6.77 (d, *J* = 8.6 Hz, 1H), 6.57 (s, 1H), 3.73 (s, 2H), 3.60 (s, 1H), 3.22 (d, *J* = 1.4 Hz, 2H), 2.62 (s, 5H); HRMS (ESI) *m/z* calculated for $C_{28}H_{30}ClN_4O_3$ $[M+H]^+$ 505.2001; found 505.2000.

1-(3,3-Diethoxypropyl)piperazine (21)

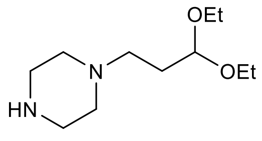

21

Piperizine (2.57 g, 29.9 mmol), 3-chloropropionaldehyde diethyl acetal (1.8 ml, 2.49 g, 15.0 mmol), and triethylamine (2.1 ml, 1.52 g, 15.0 mmol) were dissolved in EtOH (17 ml) and heated at reflux overnight. After completion of the reaction, as indicated by TLC, the reaction mixture was allowed to cool to room temperature, filtered and the solvent evaporated under reduced pressure. The crude mixture was purified by column chromatography to afford the product (1.51 g, 47%).^{1}H NMR (400 MHz, Chloroform-*d*) δ 4.56 (t, *J* = 5.7 Hz, 1H), 3.64 (dq, *J* = 9.4, 7.1 Hz, 2H), 3.49 (dq, *J* = 9.4, 7.0 Hz, 2H), 2.90 (t, *J* = 4.9 Hz, 4H), 2.51 – 2.33 (m, 6H), 1.86 – 1.75 (m, 2H), 1.19 (t, *J* = 7.1 Hz, 6H); ^{13}C NMR (100 MHz, Chloroform-*d*) δ 101.7, 61.2, 54.6, 54.4, 46.0, 30.9, 15.4; HRMS (ESI) *m/z* calculated for $C_{11}H_{25}N_2O_2$ $[M+H]^+$ 217.1911; found 217.1909.

3-((4-(3,3-Diethoxypropyl)piperazin-1-yl)methyl)-4-hydroxybenzoic acid (22)

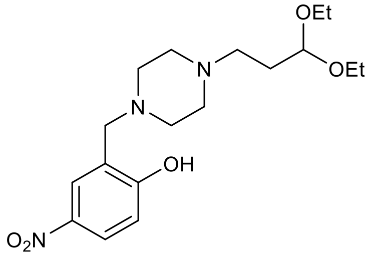

22

2-chloromethyl-4-nitrophenol hydrochloride (652 mg, 2.93 mmol) and **21** (822 mg, 3.80 mmol) were dissolved in DCM (15 ml). Triethylamine (0.5 ml, 385 mg, 3.80 mmol) was added in 3 portions and the reaction mixture was allowed to stir at room temperature overnight. After completion of the reaction, as indicated by TLC, the solvent was removed under reduced pressure and the crude mixture was purified by column chromatography to afford the product (1.04 g, 97%). ^{1}H NMR (500 MHz, Chloroform-*d*) δ 8.07 (dd, *J* = 9.0, 2.8 Hz, 1H), 7.93 (d, *J* = 2.7 Hz, 1H), 6.83 (d, *J* = 9.0 Hz, 1H), 4.56 (t, *J* = 5.6 Hz, 1H), 3.78 (s, 2H), 3.63 (dq, *J* = 9.4, 7.0 Hz, 2H), 3.53 – 3.44 (m, 2H), 2.91 – 2.27 (m, 10H), 1.85 – 1.75 (m, 2H), 1.19 (t, *J* = 7.1 Hz, 6H); ^{13}C NMR (125 MHz, Chloroform-*d*) δ 164.5, 140.1, 125.4, 124.8, 121.1, 116.5, 101.3, 61.2, 60.7, 53.6, 52.7, 52.5, 31.1, 15.3; HRMS (ESI) *m/z* calculated for $C_{18}H_{30}N_3O_5$ $[M+H]^+$ 368.2180; found 368.2180.

4-Amino-2-((4-(3,3-diethoxypropyl)piperazin-1-yl)methyl)phenol (23)

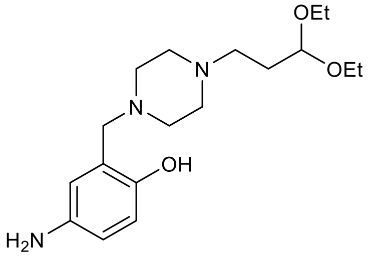

23

MeOH (10 ml) was added to a 3-neck round bottomed flask containing 10% Pd/C (35 mg), before addition of a solution of **22** (438 mg, 1.19 mmol) in MeOH (10 ml) was added. The reaction vessel was evacuated and backfilled with nitrogen 3 times, then evacuated and backfilled with H_2 3 times before being allowed to stir at room temperature for 7 days. After the completion of the reaction, as indicated by TLC, the reaction mixture was filtered over celite and the solvent removed under reduced pressure. The crude product was purified by column chromatography (385 mg, 97%). ^{1}H NMR (400 MHz, Chloroform-*d*) δ 6.65 (d, *J* = 8.4 Hz, 1H), 6.55 (dd, *J* = 8.5, 2.8 Hz, 1H), 6.38 (d, *J* = 2.8 Hz, 1H), 4.56 (t, *J* = 5.6 Hz, 1H), 3.69 – 3.56 (m, 4H), 3.49 (dq, *J* = 9.5, 7.0 Hz, 2H), 2.83 – 2.26 (m, 10H), 1.81 (q, *J* = 6.3 Hz, 2H), 1.19 (t, *J* = 7.0 Hz, 6H); ^{13}C NMR (100 MHz, Chloroform-*d*) δ 150.4, 138.5, 121.8, 116.6, 116.2, 116.0, 101.5, 61.5, 61.2, 53.8, 53.1, 52.5, 31.0, 15.4; HRMS (ESI) *m/z* calculated for $C_{18}H_{32}N_3O_3$ $[M+H]^+$ 338.2440; found 338.2430.

4-((6-Chloro-2-methoxyacridin-9-yl)amino)-2-((4-(3,3-dimethoxypropyl)piperazin-1-yl)methyl)phenol (24)

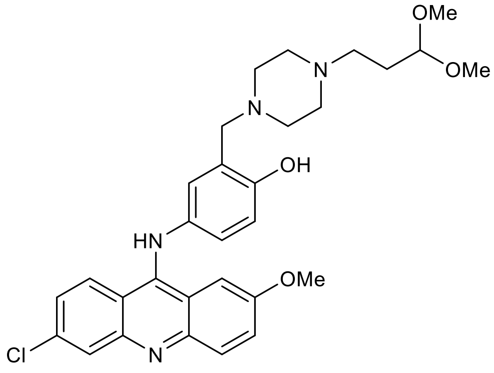

24

6,9-Dichloro-2-methoxyacridine (308 mg, 1.11 mmol) and **23** (373 mg, 1.11 mmol) were dissolved in MeOH (23 ml), 3 drops concentrated HCl were added, and the mixture was heated at reflux overnight. The reaction was quenched with saturated $NaHCO_3$ solution and extracted with DCM. The combined organic extracts were dried with $MgSO_4$, filtered, and the solvent evaporated under reduced pressure to afford the crude product as red solid. The compound was purified by column chromatography and recrystallization from MeCN (338 mg, 56%). ^{1}H NMR (500 MHz, Chloroform-*d*) δ 8.17 (s, 1H), 8.07 (d, J = 9.4 Hz, 1H), 7.92 (d, J = 9.3 Hz, 1H), 7.44 (d, J = 9.5 Hz, 1H), 7.08 (d, J = 6.4 Hz, 1H), 6.87 (d, J = 8.6 Hz, 1H), 6.80 (d, J = 8.5 Hz, 1H), 6.57 (s, 1H), 6.46 (s, 1H), 4.46 (t, J = 5.7 Hz, 1H), 3.77 (s, 2H), 3.34 (s, 6H), 2.92 – 2.34 (m, 10H), 2.20 (s, 3H), 1.81 (q, J = 6.8 Hz, 2H); HRMS (ESI) *m/z* calculated for $C_{30}H_{36}ClN_4O_4$ $[M+H]^+$ 551.2420; found 551.2418.

3-(4-(5-((6-Chloro-2-methoxyacridin-9-yl)amino)-2-hydroxybenzyl)piperazin-1-yl)propanal (25)

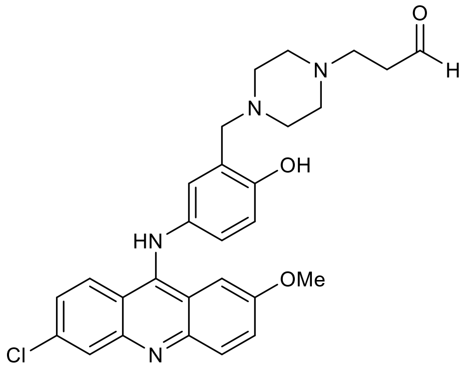

25

Compound **24** (177 mg, 0.31 mmol) was dissolved in DCM (10 ml) and cooled to 0 °C. 1M BBr_3 in DCM (0.7 ml) was added dropwise and allowed to stir at 0 °C for 15 minutes. After completion of the reaction, as indicated by TLC, the reaction was carefully quenched by addition of H_2O and the pH adjusted to 8 with a saturated $NaHCO_3$ solution. The reaction mixture was extracted with DCM and the combined organic extracts dried with Na_2SO_4, filtered, and evaporated. The crude product was purified by column chromatography to afford the product as an orange solid (153 mg, 97%). The impure mixture obtained was used without further purification.

Methyl 2,2-dimethyl-3-oxopropanoate (27)[1]

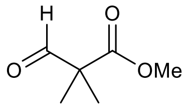

27

A solution of oxalyl chloride (0.39 ml, 552 mg, 4.35 mmol) in DCM (9ml) was cooled to -78 °C. A mixture of DMSO (0.4 ml, 443 mg, 5.67 mmol) and DCM (0.5 ml) was added dropwise and allowed to stir for 5 minutes before addition of a solution of methyl 3-hydroxy-2,2-dimethylpropanoate (500 mg, 3.78 mmol) in DCM (2 ml) was added dropwise and allowed to stir for a further 5 minutes before the dropwise addition of triethylamine (2.62 ml, 1.91 g, 18.9 mmol). The reaction mixture was slowly allowed to warm to room temperature. After completion of the reaction, as indicated by TLC, Et_2O and H_2O were added and the combined organic extracts were dried with $MgSO_4$, filtered, and evaporated. The product was purified by column chromatography (169 mg, 34%). ^1H NMR (500 MHz, Chloroform-*d*) δ 9.67 (s, 1H), 3.77 (s, 3H), 1.37 (s, 6H); ^{13}C NMR (125 MHz, Chloroform-*d*) δ 199.0, 173.2, 53.9, 52.6, 19.7; HRMS (EI) *m/z* calculated for $C_6H_{11}O_3$ $[M+H]^+$ 131.0708; found 131.0710.

4-Nitro-2-(piperazin-1-ylmethyl)phenol (28)

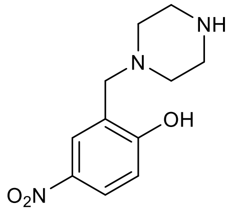

28

2-Hydroxy-5-nitrobenzaldehyde (1.0 g, 6.0 mmol) was dissolved in DCM (50 ml). Boc-piperazine (1.2 g, 6.6 mmol) and acetic acid (0.10 ml, 0.11 g, 1.8 mmol) were added and allowed to stir for 30 minutes before addition of $NaBH(OAc)_3$ (1.5 g, 7.2 mmol). The reaction mixture was allowed to stir at room temperature overnight. After the completion of the reaction, as indicated by TLC, the reaction was quenched with satd. $NaHCO_3$ solution, then 1 M NaOH solution. The reaction mixture was then adjusted to pH = 8-9 by addition of 1M HCl. Extraction with DCM and drying of organic extracts with Na_2SO_4, filtration and removal of solvent under reduced pressure afforded the crude boc-protected product, which was purified by column chromatography (1.74 g, 86%). The boc-protected product was dissolved in DCM (20 ml) and TFA (4 ml) and allowed to stir at room temperature for 2 days. After the completion of the reaction, as indicated by TLC, the reaction mixture was quenched with a saturated solution of $NaHCO_3$ and extracted with DCM. The combined organic extracts were dried with $MgSO_4$, filtered, and the solvent removed under reduced pressure. The crude product was purified by column chromatography (quantitative yield). ^1H NMR (700 MHz, Chloroform-*d*) δ 8.09 (dd, *J* = 9.0, 2.8 Hz, 1H), 7.94 (dd, *J* = 2.8, 0.9 Hz, 1H), 6.84 (d, *J* = 8.9 Hz, 1H), 3.78 (s, 2H), 3.01 – 2.43 (m, 9H); ^{13}C NMR (175 MHz, Chloroform-*d*) δ 164.4, 125.4, 124.8,

[1]Ng SS, Ho CY, Jamison TF. Nickel-catalyzed coupling of alkenes, aldehydes, and silyl triflates. *J Am Chem Soc* (2006) **128**:11513–11528. doi:10.1021/ja062866w

121.0, 116.5, 61.3, 53.5, 53.4, 45.7; HRMS (ESI) *m/z* calculated for $C_{11}H_{16}N_3O_3$ $[M+H]^+$ 238.1186; found 238.1184.

Methyl 3-(4-(2-hydroxy-5-nitrobenzyl)piperazin-1-yl)-2,2-dimethylpropanoate (29)

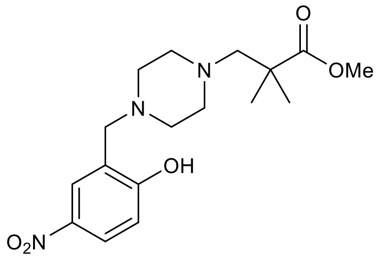

29

Compound **27** (470 mg, 3.61 mmol) was dissolved in DCM (25 ml). Compound **28** (777 mg, 3.28 mmol) and acetic acid (0.10 ml, 0.11 g, 1.8 mmol) were added and allowed to stir for 30 minutes before addition of $NaBH(OAc)_3$ (56µl, 59 mg, 1.0 mmol). The reaction mixture was allowed to stir at room temperature overnight. After the completion of the reaction, as indicated by TLC, the reaction was quenched with satd. $NaHCO_3$ solution, then 1 M NaOH solution. The reaction mixture was then adjusted to pH = 8-9 by addition of 1M HCl. Extraction with DCM and drying of organic extracts with Na_2SO_4, filtration and removal of solvent under reduced pressure afforded the crude product, which was purified by column chromatography (990 mg, 86%). ^{1}H NMR (400 MHz, Chloroform-*d*) δ 8.08 (dd, *J* = 9.0, 2.8 Hz, 1H), 7.92 (d, *J* = 2.7 Hz, 1H), 6.83 (d, *J* = 9.0 Hz, 1H), 3.76 (s, 2H), 3.65 (s, 3H), 2.68 – 2.46 (m, 8H), 1.16 (s, 6H).

3-(4-(2-Hydroxy-5-nitrobenzyl)piperazin-1-yl)-2,2-dimethylpropanoic acid (30)

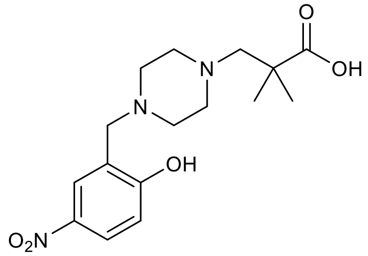

30

Compound **29** (515 mg, 1.50 mmol) was dissolved in THF (4 ml) and H_2O (4 ml). $LiOH.H_2O$ (123 mg, 2.93 mmol) was added and the reaction was heated at reflux for 6 hours. The reaction mixture was allowed to cool to room temperature and the THF was evaporated under reduced pressure. Extractions of the aqueous layer with DCM at pH = 14, pH = 1, and pH = 7 were carried out and the combined organic extracts were dried with $MgSO_4$, filtered, and evaporated under reduced pressure to afford the desired product (280 mg, 57%). ^{1}H NMR (700 MHz, Chloroform-*d*) δ 8.11 (dd, *J* = 9.0, 2.7 Hz, 1H), 7.96 (d, *J* = 2.7 Hz, 1H), 6.87 (d, *J* = 9.0 Hz, 1H), 3.83 (s, 2H), 2.96 – 2.55 (m, 8H), 1.23 (s, 6H); ^{13}C NMR (175 MHz, Chloroform-*d*) δ 177.6, 163.7, 140.5, 125.6, 124.9, 120.5, 116.6, 65.2, 60.5, 54.4, 52.2, 41.5, 25.2.

3-(4-(2-Hydroxy-5-nitrobenzyl)piperazin-1-yl)-N,2,2-trimethylpropanamide (31)

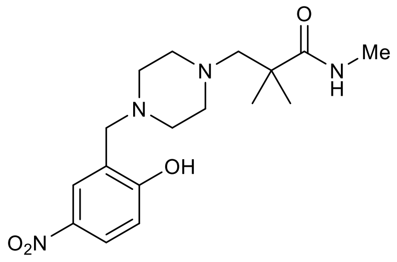

31

Compound **30** (236 mg, 0.78 mmol) was dissolved in DCM (7 ml). DMF (7 drops) then thionyl chloride (0.17 ml, 279 mg, 2.34 mmol) was added and the reaction mixture allowed to stir at room temperature overnight. After the completion of the reaction, as indicated by TLC, the solvent was evaporated under reduced pressure to afford the crude acid chloride as a colourless solid, which was dissolved in DCM (20 ml). Methylamine (2M in THF, 2.0 ml) was added and the reaction mixture was allowed to stir at room temperature overnight. After the completion of the reaction, as indicated by TLC, the reaction was quenched with H_2O and extracted with DCM. The combined organic extracts were washed with brine, dried with $MgSO_4$, filtered, and evaporated under reduced pressure. The crude product was purified by column chromatography to afford the desired product (256 mg, 84%). ^{1}H NMR (500 MHz, Chloroform-*d*) δ 8.07 (dd, *J* = 9.0, 2.8 Hz, 1H), 7.92 (d, *J* = 2.8 Hz, 1H), 6.94 (s, 1H), 6.83 (d, *J* = 9.0 Hz, 1H), 3.77 (s, 2H), 2.76 (d, *J* = 4.8 Hz, 3H), 2.72 – 2.40 (m, 9H), 1.13 (s, 6H); ^{13}C NMR (125 MHz, Chloroform-*d*) δ 177.8, 164.3, 140.2, 125.4, 124.8, 121.0, 116.6, 66.4, 60.6, 54.6, 52.8, 42.6, 26.2, 24.4; HRMS (ESI) *m/z* calculated for $C_{17}H_{27}N_4O_4$ $[M+H]^+$ 351.2027; found 351.2028.

3-(4-(5-Amino-2-hydroxybenzyl)piperazin-1-yl)-N,2,2-trimethylpropanamide (32)

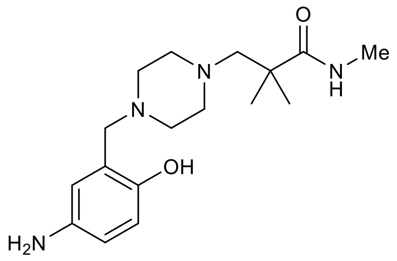

32

MeOH (5 ml) was added to a 3-neck round bottomed flask containing 10% Pd/C (20 mg), before addition of a solution of **31** (148 mg, 0.42 mmol) in MeOH (5 ml) was added. The reaction vessel was evacuated and backfilled with nitrogen 3 times, then evacuated and backfilled with H_2 3 times before being allowed to stir at room temperature for 7 days. After the completion of the reaction, as indicated by TLC, the reaction mixture was filtered over celite and the solvent removed under reduced pressure. The crude product was purified by column chromatography (77 mg, 57%). ^{1}H NMR (700 MHz, Chloroform-*d*) δ 6.65 (d, *J* = 8.4 Hz, 1H), 6.55 (dd, *J* = 8.4, 2.8 Hz, 1H), 6.36 (d, *J* = 2.8 Hz, 1H), 3.59 (s, 2H), 2.76 (d, *J* = 4.8 Hz, 3H), 2.70 – 2.38 (m, 9H), 1.12 (s, 6H); HRMS (ESI) *m/z* calculated for $C_{17}H_{29}N_4O_2$ $[M+H]^+$ 321.2285; found 321.2284.

***Tert*-butyl 4-(2-methoxyethyl)piperazine-1-carboxylate (33)**

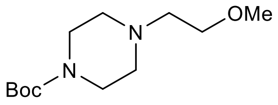

33

1-(2-Hydroxyethyl)piperazine (1.0 g, 7.7 mmol) was dissolved in MeOH (22 ml). Boc_2O (1.8 ml, 1.7 g, 7.7 mmol) was added dropwise and the reaction mixture was allowed to stir at room temperature overnight. The solvent was evaporated to afford crude *tert*-butyl 4-(2-hydroxyethyl)piperazine-1-carboxylate whcih was used without further purification. NaH (40 %, 88 mg, 2.2 mmol) was dissolved in THF (10 ml) and cooled to 0 °C, *tert*-butyl 4-(2-hydroxyethyl)piperazine-1-carboxylate (421 mg, 1.8 mmol) in THF (10 ml) was added and the reaction mixture was allowed to stir at 0 °C for 1 hour. Methyl iodide (137 µl, 312 mg, 2.2 mmol) was added and the reaction mixture was allowed to warm to room temperature and stir overnight. After the completion of the reaction, as indicated by TLC, the reaction was quenched with H_2O and extracted with EtOAc. The combined organic extracts were dried with Na_2SO_4, filtered, the solvent removed under reduced pressure to afford the product (422 mg, 95%). ^1H NMR (400 MHz, Chloroform-*d*) δ 3.51 (t, J = 5.6 Hz, 2H), 3.45 (t, J = 5.0 Hz, 4H), 3.35 (s, 3H), 2.58 (t, J = 5.6 Hz, 2H), 2.43 (t, J = 5.1 Hz, 4H), 1.45 (s, 9H); ^{13}C NMR (100 MHz, Chloroform-*d*) δ 154.8, 79.6, 70.1, 59.0, 58.1, 53.4, 28.5, -9.1.

1-(2-Methoxyethyl)piperazine (TFA salt) (34)

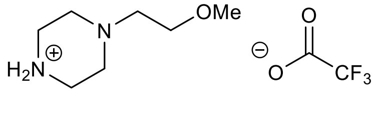

34

Compound **33** (412 mg, 1.69 mmol) was dissolved in DCM (7.0 ml) and TFA (1.4 ml) and allowed to stir at room temperature overnight. After the completion of the reaction, as indicated by TLC, DCM was evaporated and residual TFA was co-evaporated with toluene to afford the product as a beige solid that was used without further purification (quantitative yield).

4-Amino-2-((4-(2-methoxyethyl)piperazin-1-yl)methyl)phenol (35)

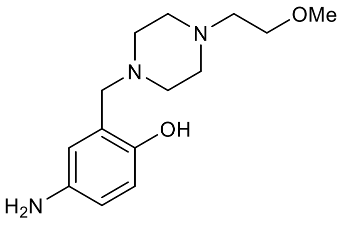

35

Compound **34** (630 mg, 2.4 mmol), acetaminophen (250 mg, 1.6 mmol), and formaldehyde (37% aq. Soln, 0.8ml) were dissolved in isopropanol (12 ml). The reaction mixture was heated at reflux overnight. After the completion of the reaction, as indicated by TLC, the solvent was evaporated under reduced pressure and the crude mixture purified by column chromatography to afford *N*-(4-

hydroxy-3-((4-(2-methoxyethyl)piperazin-1-yl)methyl)phenyl)acetamide (192 mg, 56%), which was used without further purification. *N*-(4-hydroxy-3-((4-(2-methoxyethyl)piperazin-1-yl)methyl)phenyl)acetamide (166 mg, 0.54 mmol) was dissolved in 6M aqueous HCl (1.5 ml). The reaction was heated at reflux for 2 hours. After the completion of the reaction, as indicated by TLC, the reaction mixture was allowed to cool to room temperature, neutralized and extracted with DCM. The combined organic extracts were dried with $MgSO_4$, filtered, and the solvent evaporated under reduced pressure. The crude product was purified by column chromatography (98 mg, 68%). ^{1}H NMR (400 MHz, Chloroform-*d*) δ 6.65 (d, *J* = 8.4 Hz, 1H), 6.55 (dd, *J* = 8.4, 2.8 Hz, 1H), 6.38 (d, *J* = 2.8 Hz, 1H), 3.61 (s, 2H), 3.51 (t, *J* = 5.5 Hz, 2H), 3.35 (s, 3H), 2.84 – 2.39 (m, 10H); HRMS (ESI) *m/z* calculated for $C_{14}H_{24}N_3O_2$ $[M+H]^+$ 266.1863; found 266.1864.

NMR Spectra

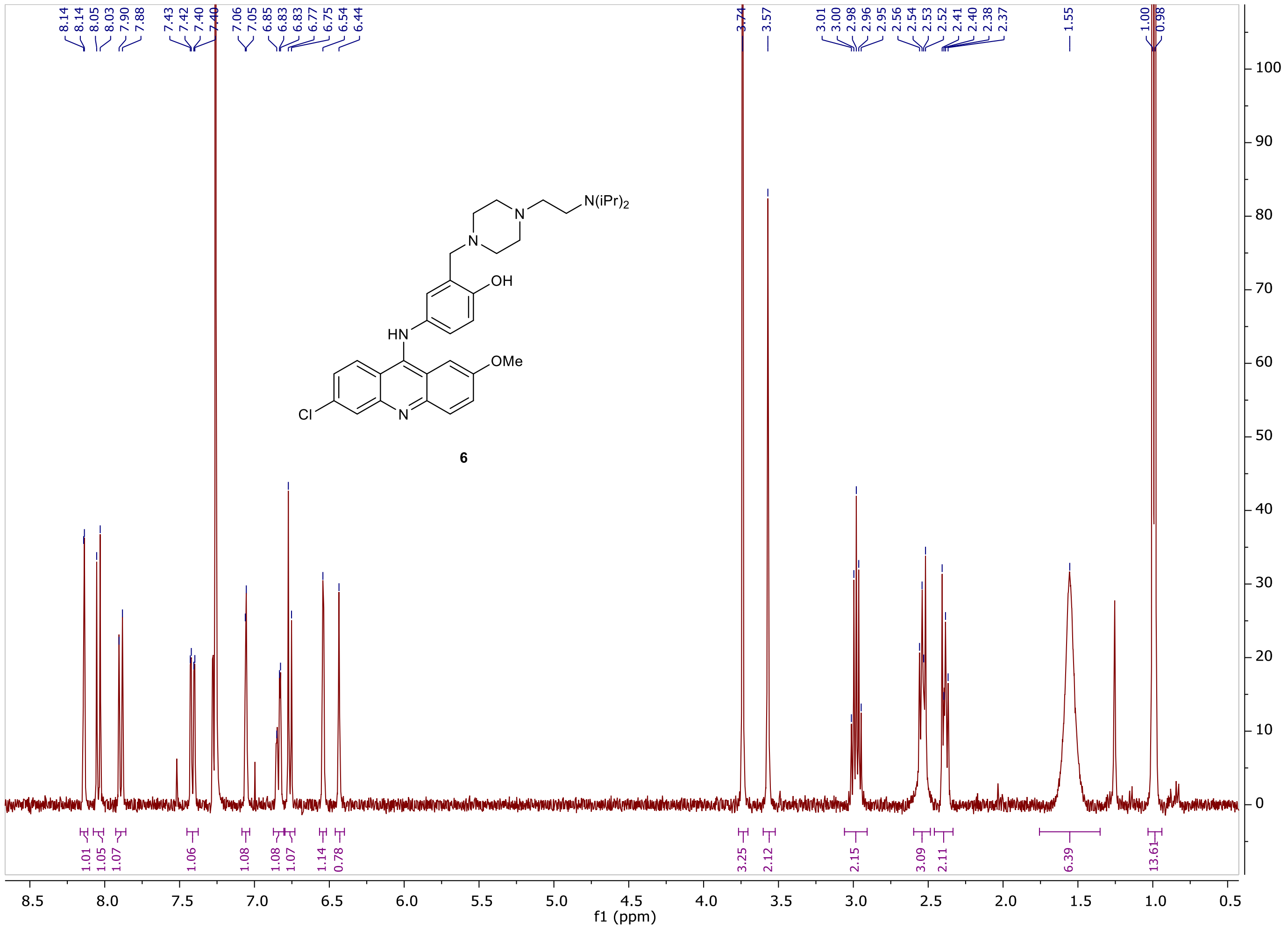
N(iPr)2
OH
HN
OMe
Cl
N
6
f1 (ppm)

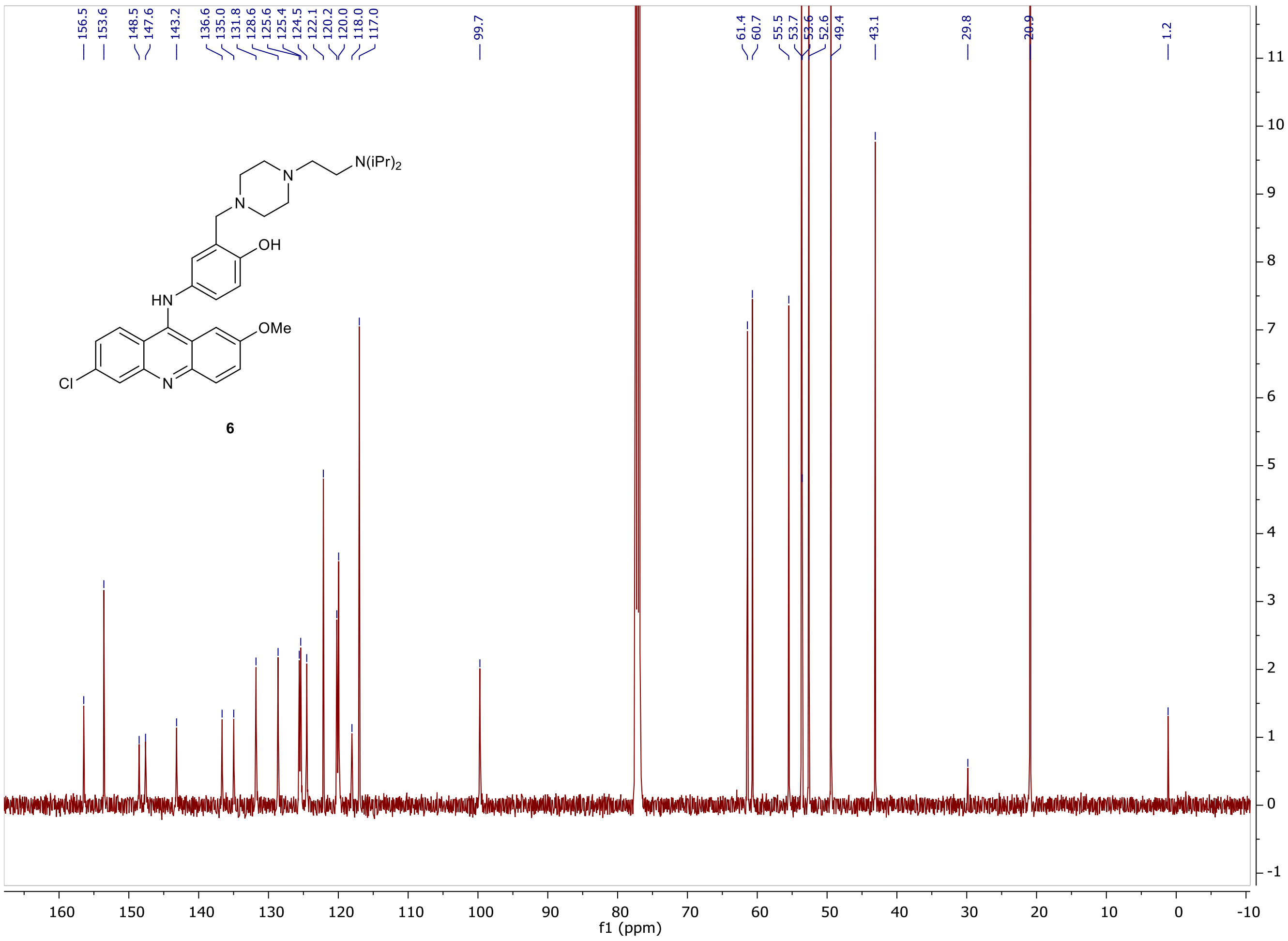
156.5
153.6
148.5
147.6
143.2
136.6
135.0
131.8
128.6
125.6
125.4
124.5
122.1
120.2
120.0
118.0
117.0
99.7
61.4
60.7
55.5
53.7
53.6
52.6
49.4
43.1
29.8
20.9
1.2
N(iPr)2
N
OH
HN
OMe
Cl
6
f1 (ppm)

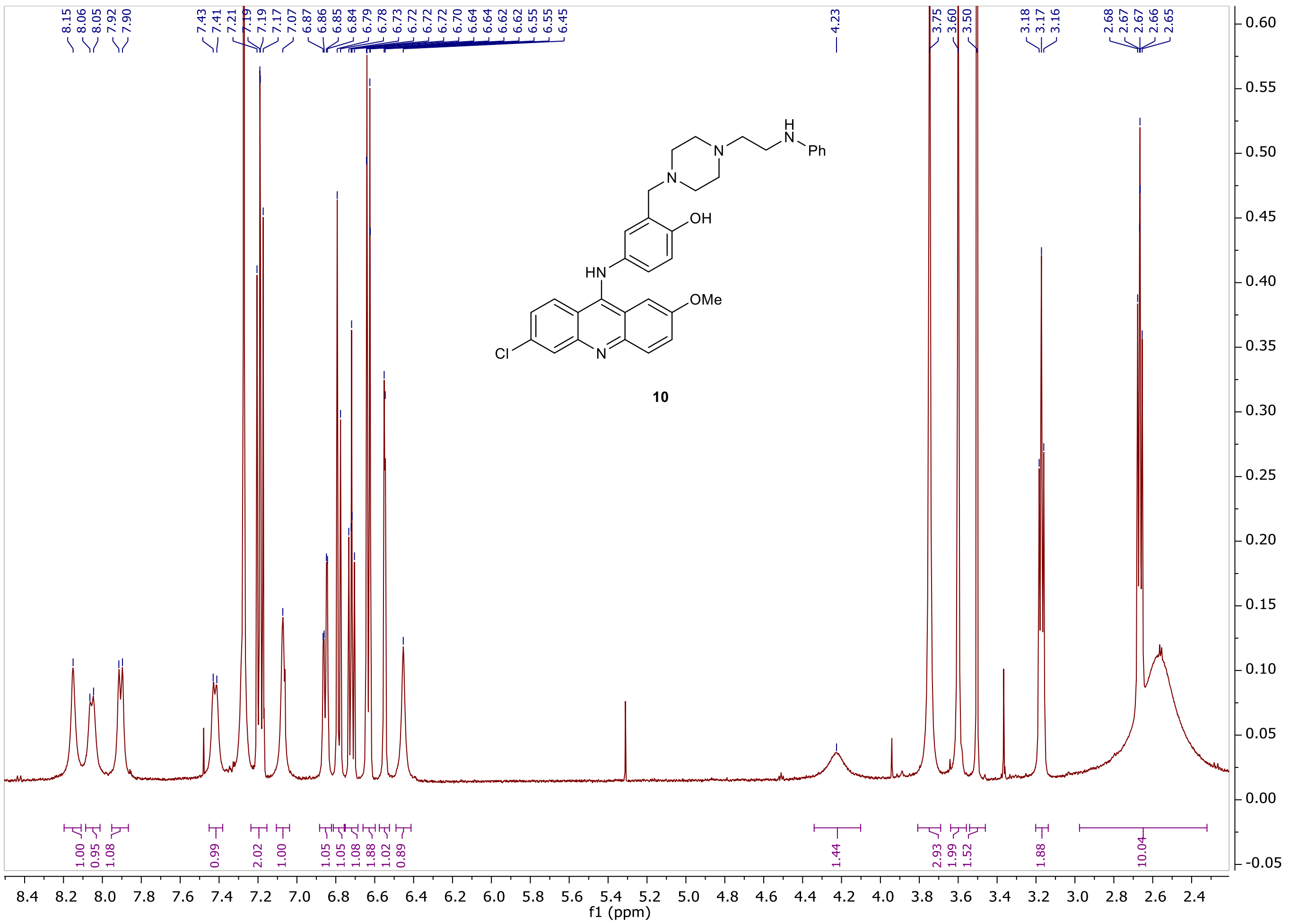

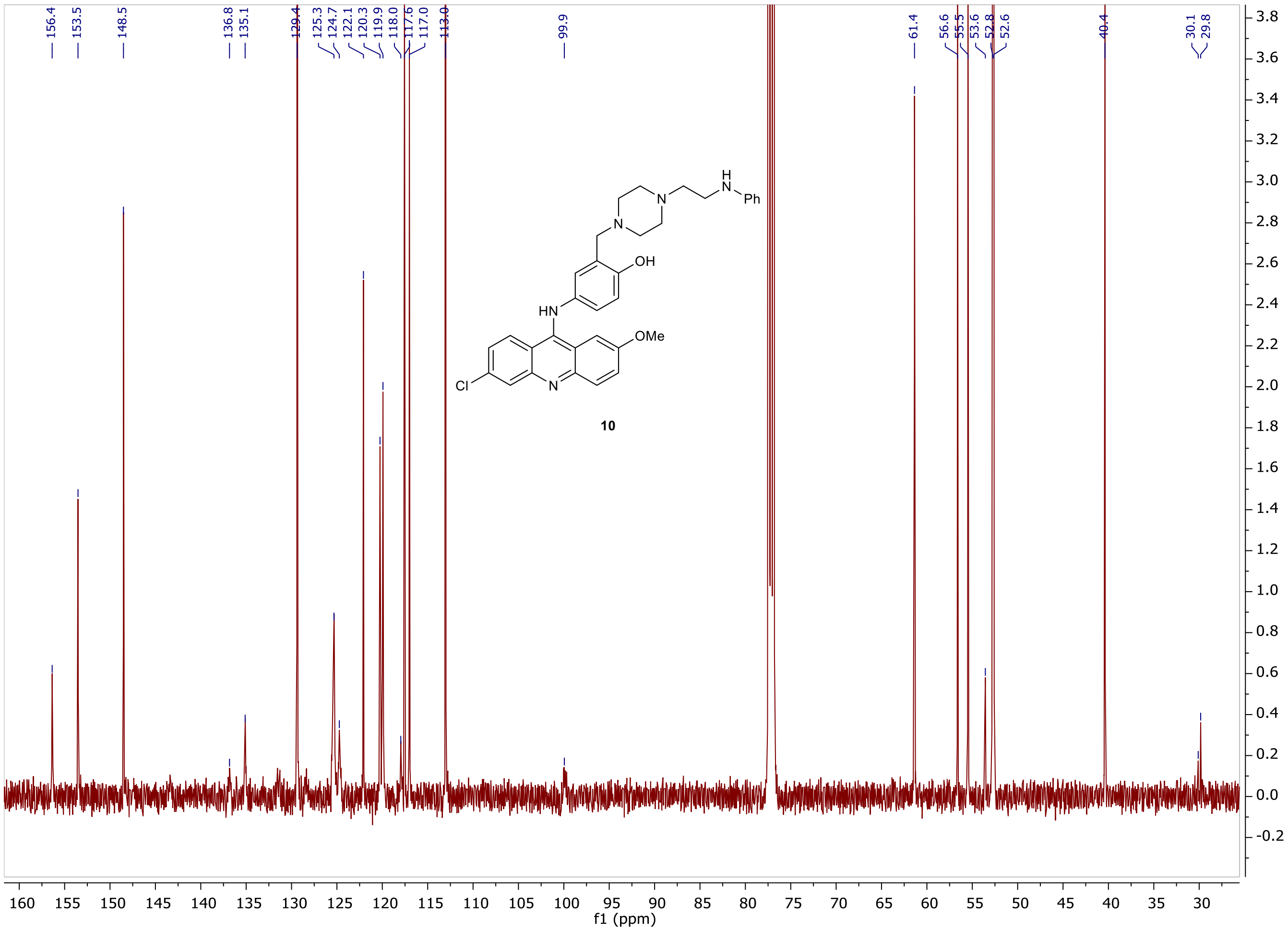
156.4
153.5
148.5
136.8
135.1
129.4
125.3
124.7
122.1
120.3
119.9
118.0
117.6
117.0
113.0
99.9
61.4
56.6
55.5
53.6
52.8
52.6
40.4
30.1
29.8
H
N
Ph
N
N
OH
HN
OMe
Cl
N
10
f1 (ppm)

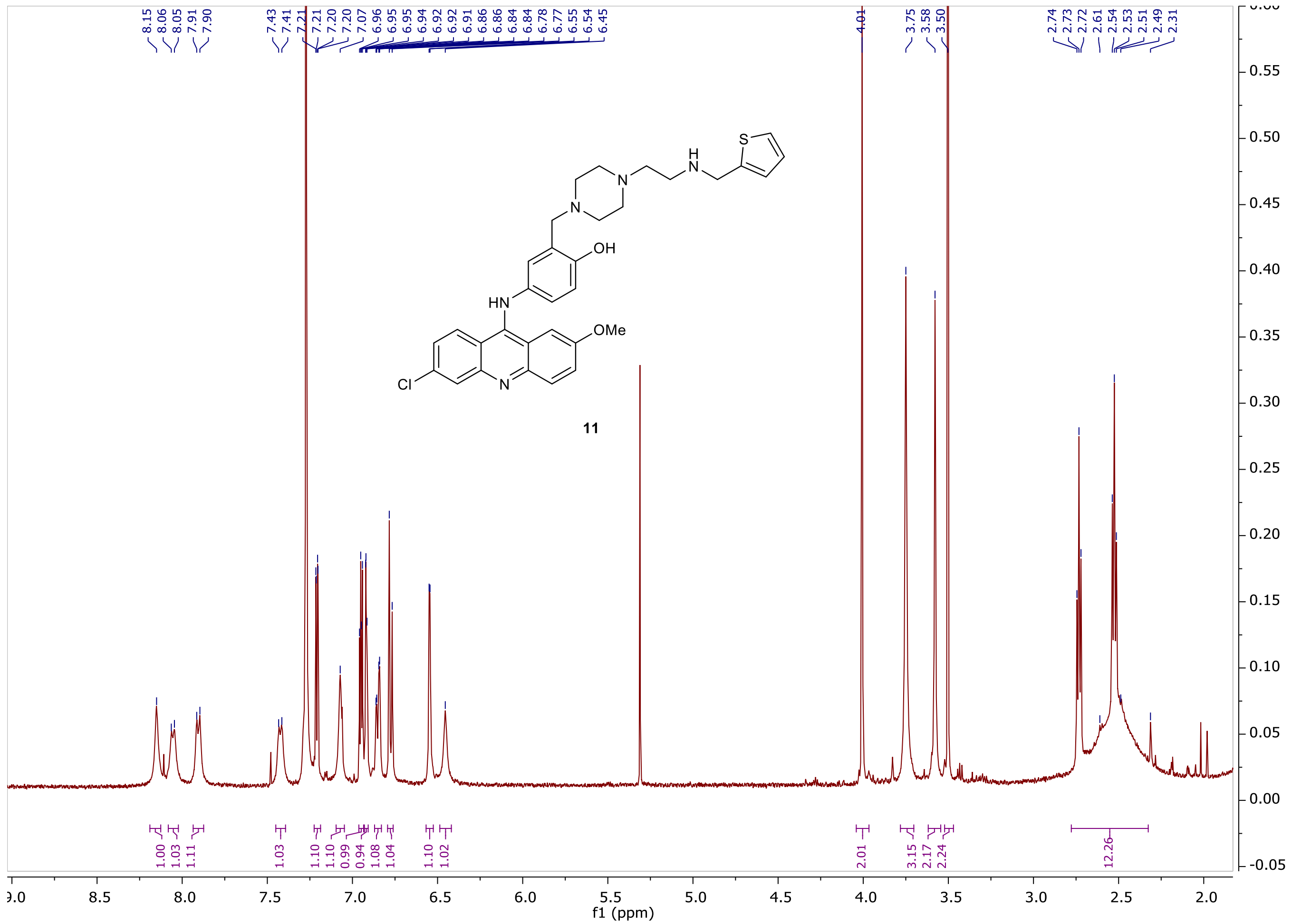
11
f1 (ppm)

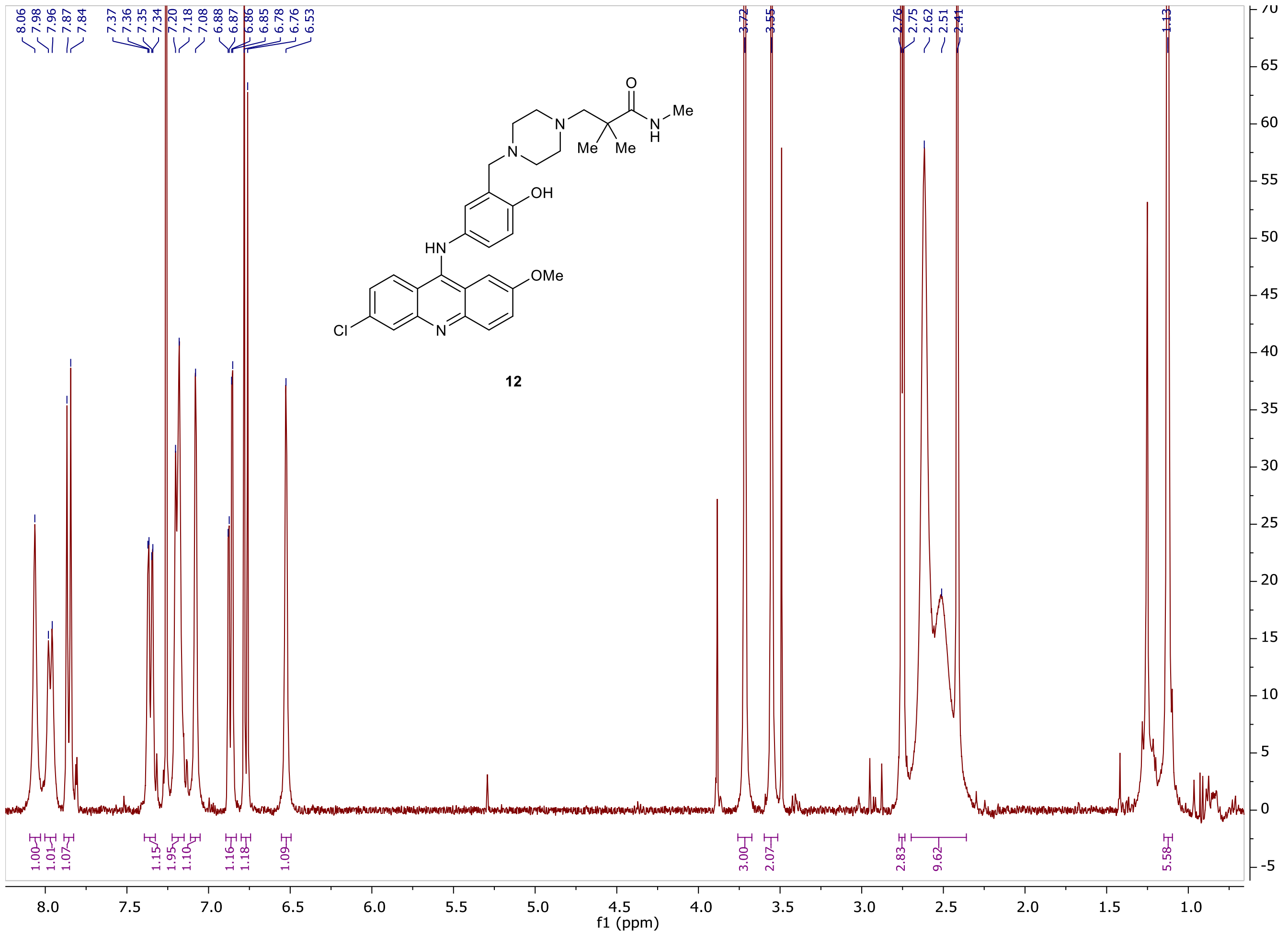
8.06
7.98
7.96
7.87
7.84
7.37
7.36
7.35
7.34
7.20
7.18
7.08
6.88
6.87
6.86
6.85
6.78
6.76
6.53
3.72
3.55
2.76
2.75
2.62
2.51
2.41
1.13
OH
HN
OMe
Cl
N
Me
O
H
12
1.00
1.01
1.07
1.15
1.95
1.10
1.16
1.18
1.09
3.00
2.07
2.83
9.62
5.58
f1 (ppm)

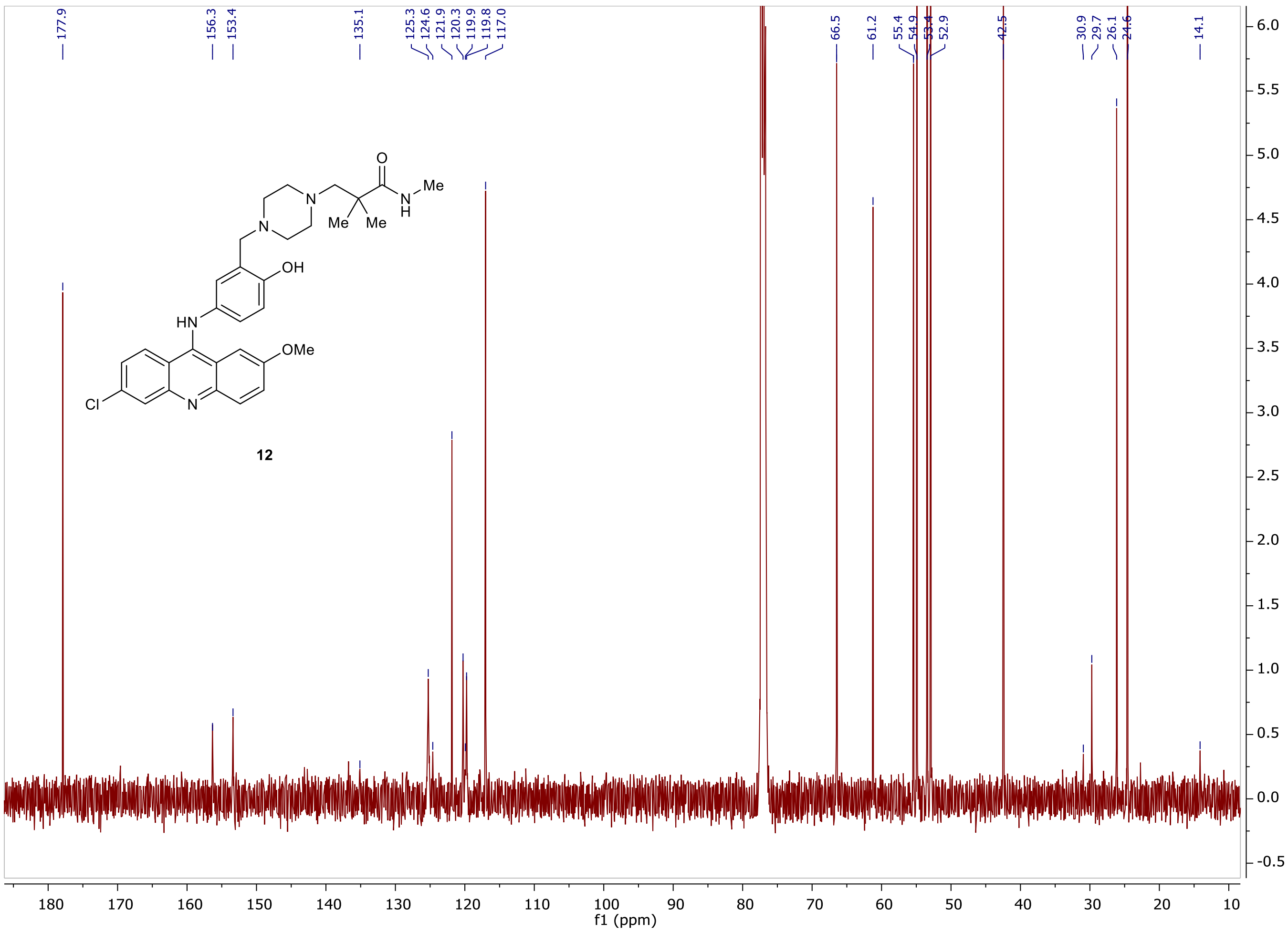
177.9
156.3
153.4
135.1
125.3
124.6
121.9
120.3
119.9
119.8
117.0
66.5
61.2
55.4
54.9
53.4
52.9
42.5
30.9
29.7
26.1
24.6
14.1
f1 (ppm)
O
N
Me
H
Me
Me
OH
HN
OMe
Cl
N

12

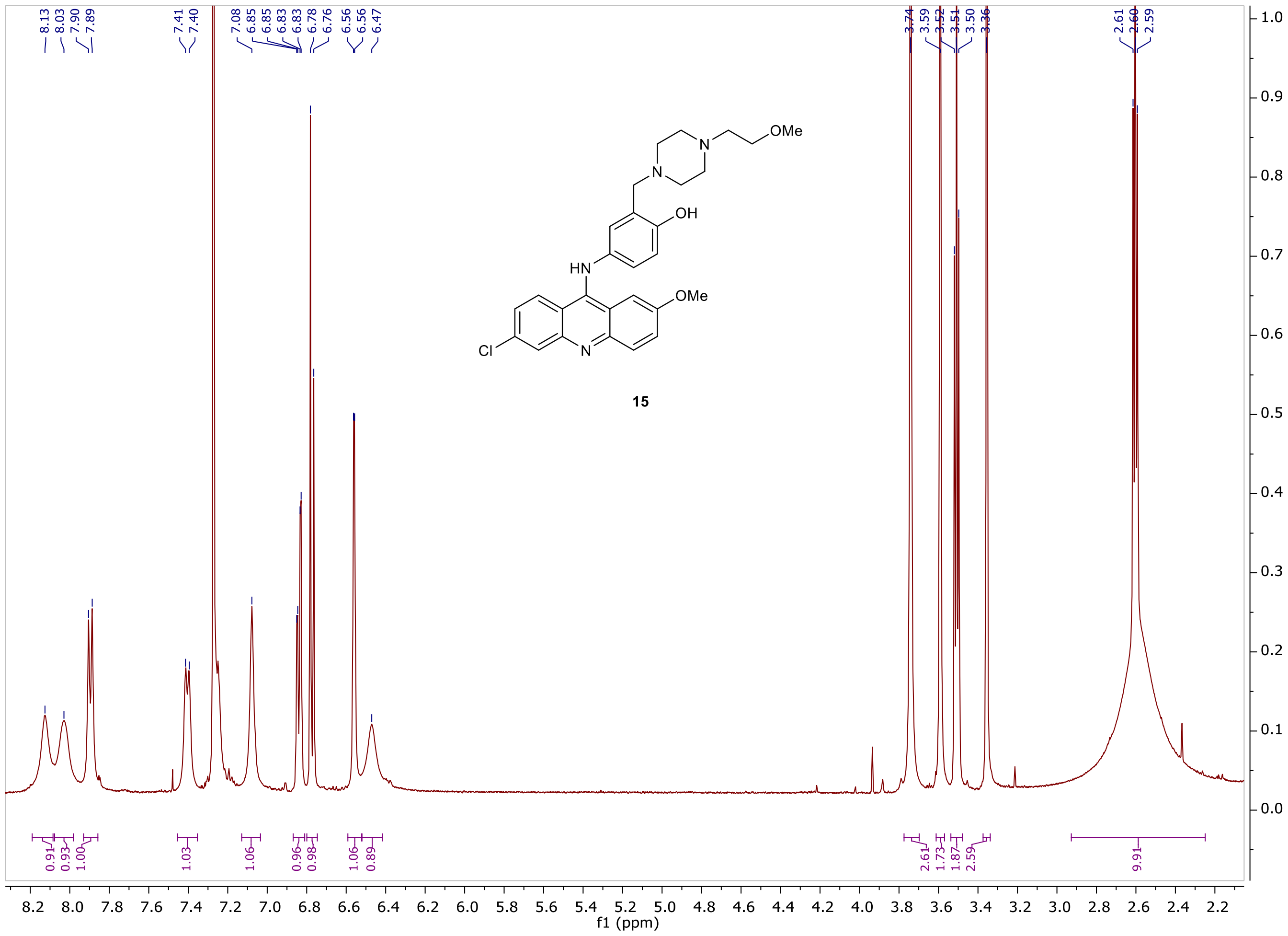
8.13
8.03
7.90
7.89
7.41
7.40
7.08
6.85
6.85
6.83
6.83
6.78
6.76
6.56
6.56
6.47
3.74
3.59
3.52
3.51
3.50
3.36
2.61
2.60
2.59
OMe
N
N
OH
HN
OMe
Cl
N
15
0.91
0.93
1.00
1.03
1.06
0.96
0.98
1.06
0.89
2.61
1.73
1.87
2.59
9.91
f1 (ppm)

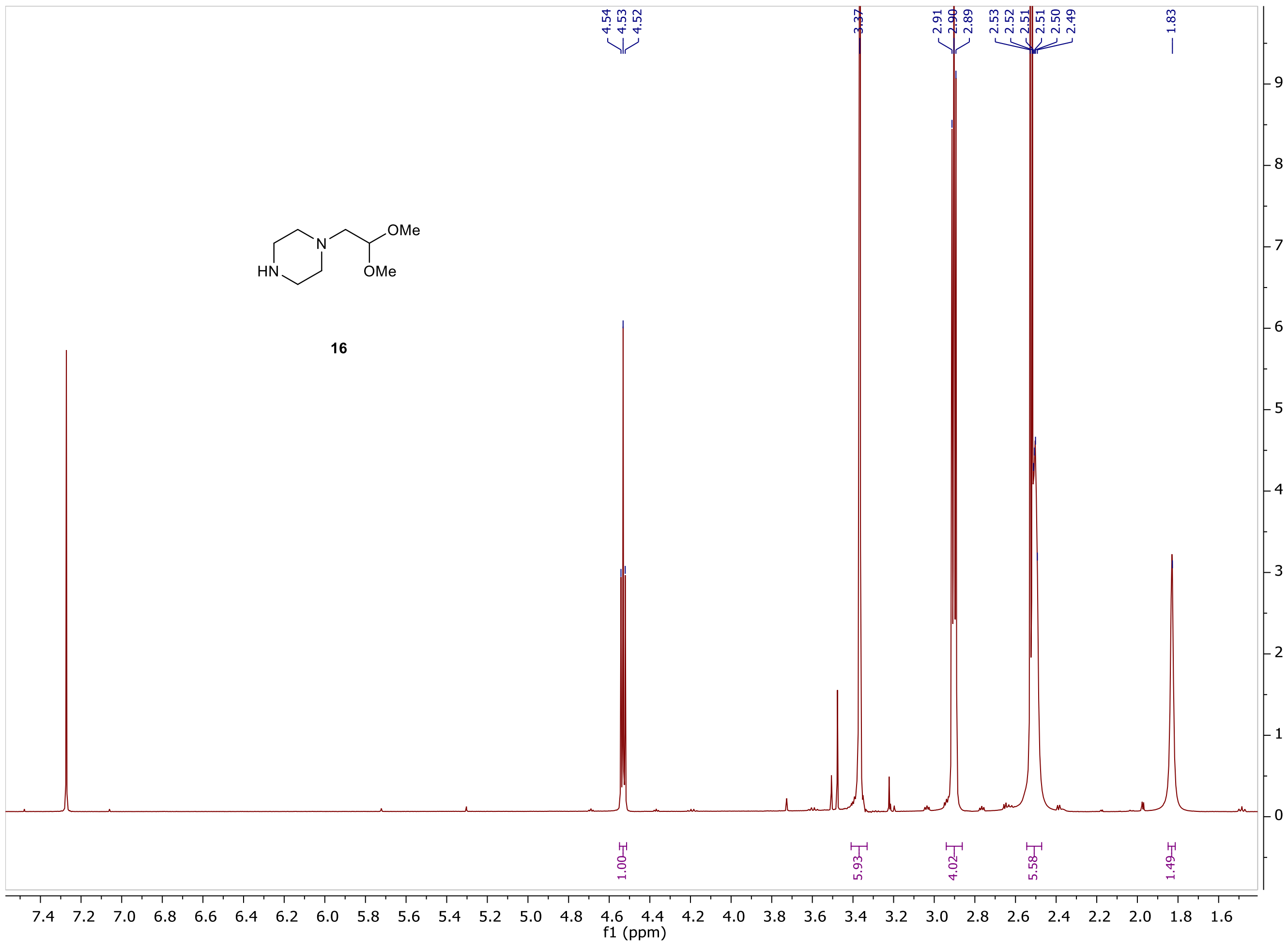
4.54
4.53
4.52
3.37
2.91
2.90
2.89
2.53
2.52
2.51
2.51
2.50
2.49
1.83
OMe
N
HN
OMe
16
1.00
5.93
4.02
5.58
1.49
7.4 7.2 7.0 6.8 6.6 6.4 6.2 6.0 5.8 5.6 5.4 5.2 5.0 4.8 4.6 4.4 4.2 4.0 3.8 3.6 3.4 3.2 3.0 2.8 2.6 2.4 2.2 2.0 1.8 1.6
f1 (ppm)
9 8 7 6 5 4 3 2 1 0

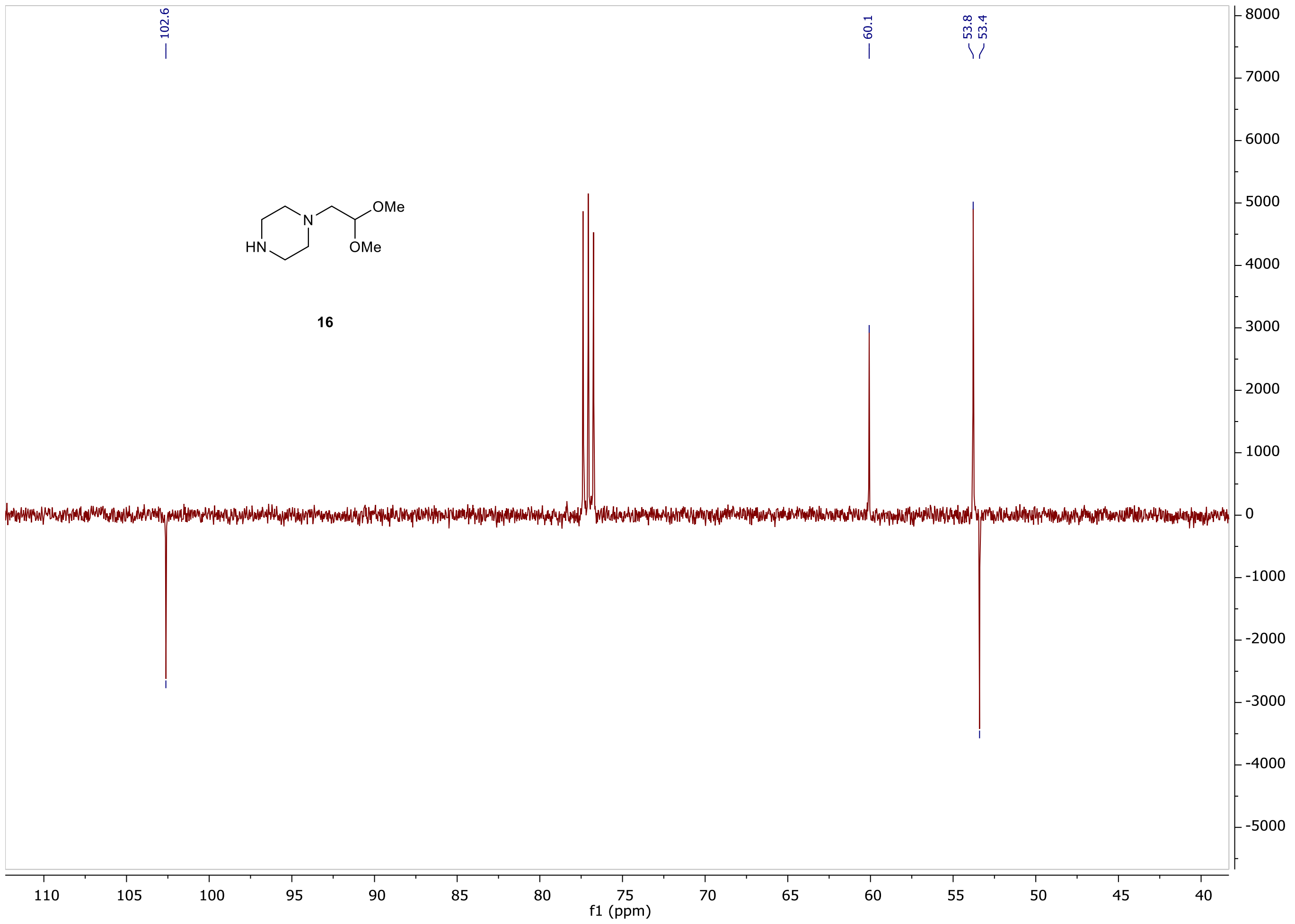

102.6
60.1
53.8
53.4
OMe
N
HN
OMe
16
8000
7000
6000
5000
4000
3000
2000
1000
0
-1000
-2000
-3000
-4000
-5000
110
105
100
95
90
85
80
75
70
65
60
55
50
45
40
f1 (ppm)

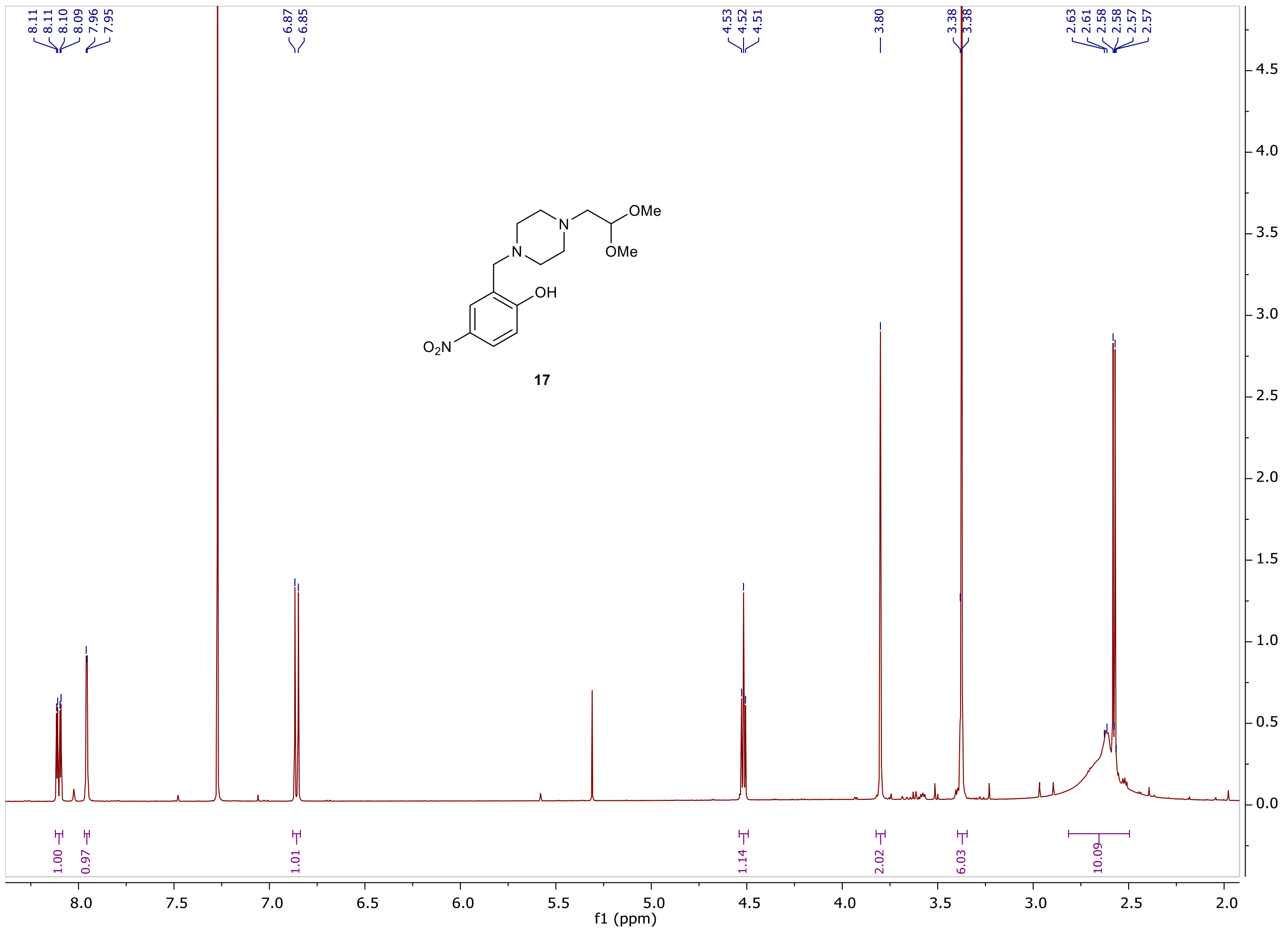
8.11
8.11
8.10
8.09
7.96
7.95
6.87
6.85
4.53
4.52
4.51
3.80
3.38
3.38
2.63
2.61
2.58
2.58
2.57
2.57
OMe
N
N
OMe
OH
O_2N
17
1.00
0.97
1.01
1.14
2.02
6.03
10.09
4.5
4.0
3.5
3.0
2.5
2.0
1.5
1.0
0.5
0.0
8.0
7.5
7.0
6.5
6.0
5.5
5.0
4.5
4.0
3.5
3.0
2.5
2.0
f1 (ppm)

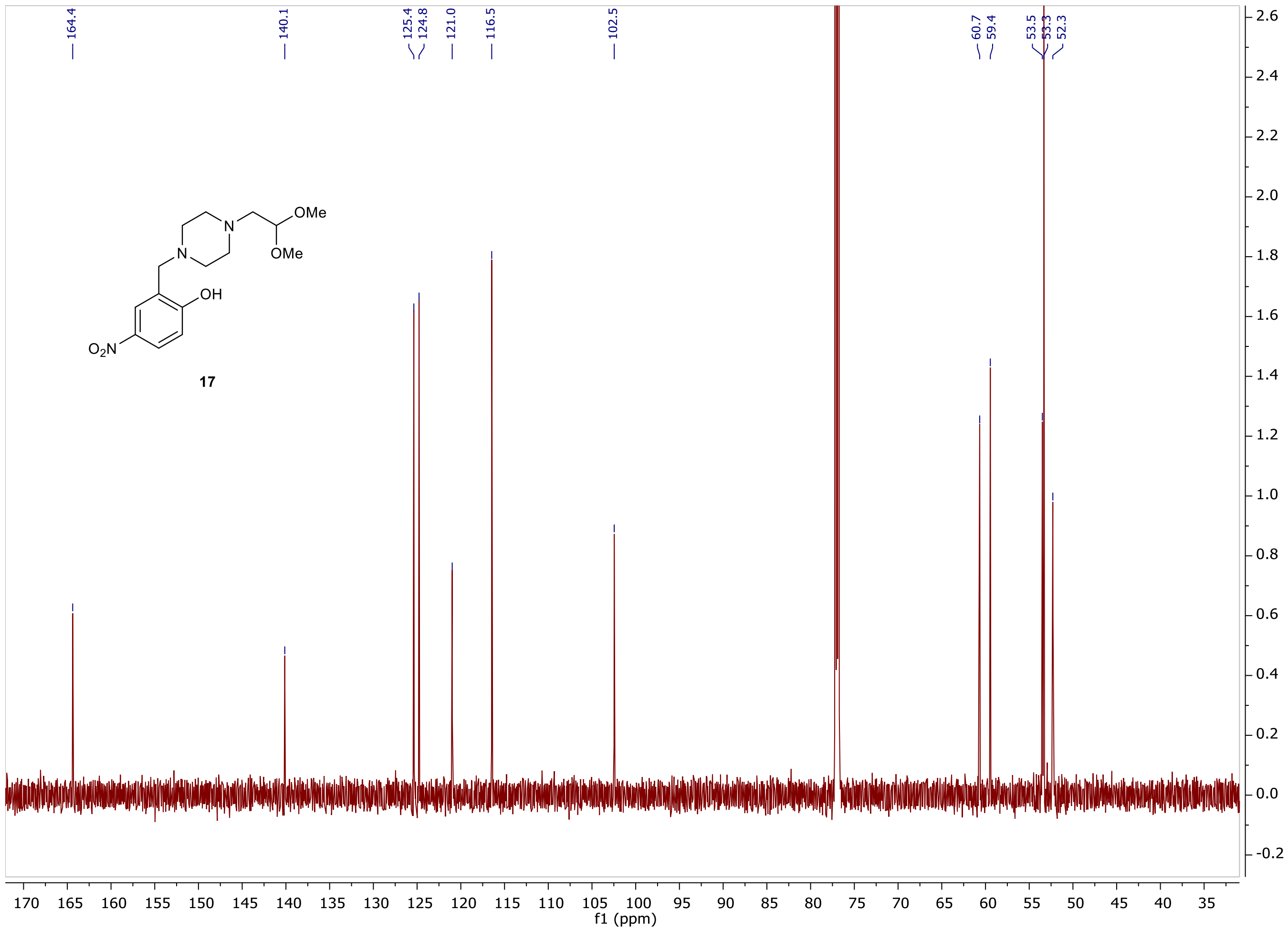

164.4
140.1
125.4
124.8
121.0
116.5
102.5
60.7
59.4
53.5
53.3
52.3
OMe
OMe
N
N
OH
O2N
17
f1 (ppm)

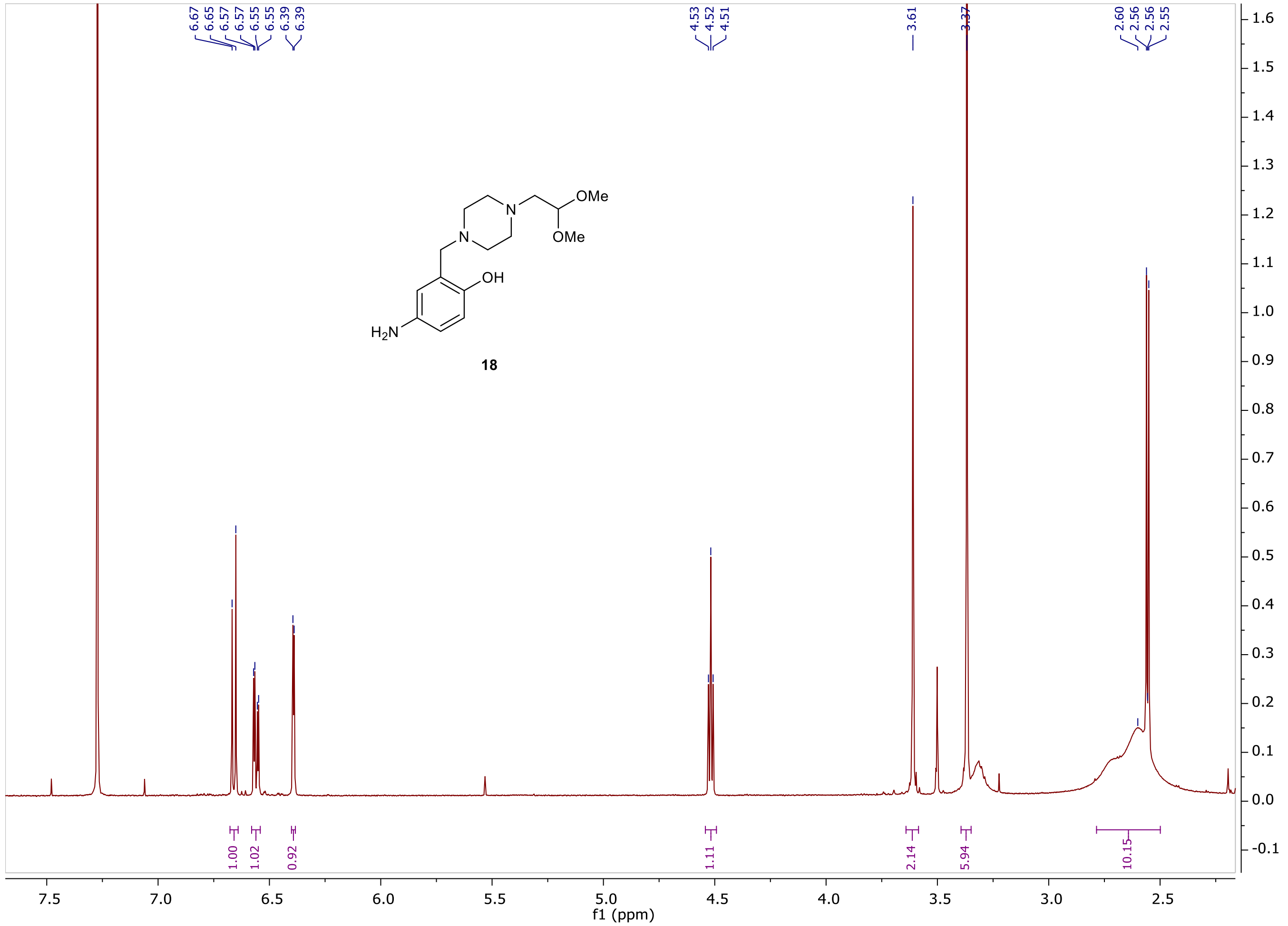
6.67
6.65
6.57
6.57
6.55
6.55
6.39
6.39
4.53
4.52
4.51
3.61
3.37
2.60
2.56
2.56
2.55
OMe
OMe
N
N
OH
H2N
18
1.00
1.02
0.92
1.11
2.14
5.94
10.15
f1 (ppm)

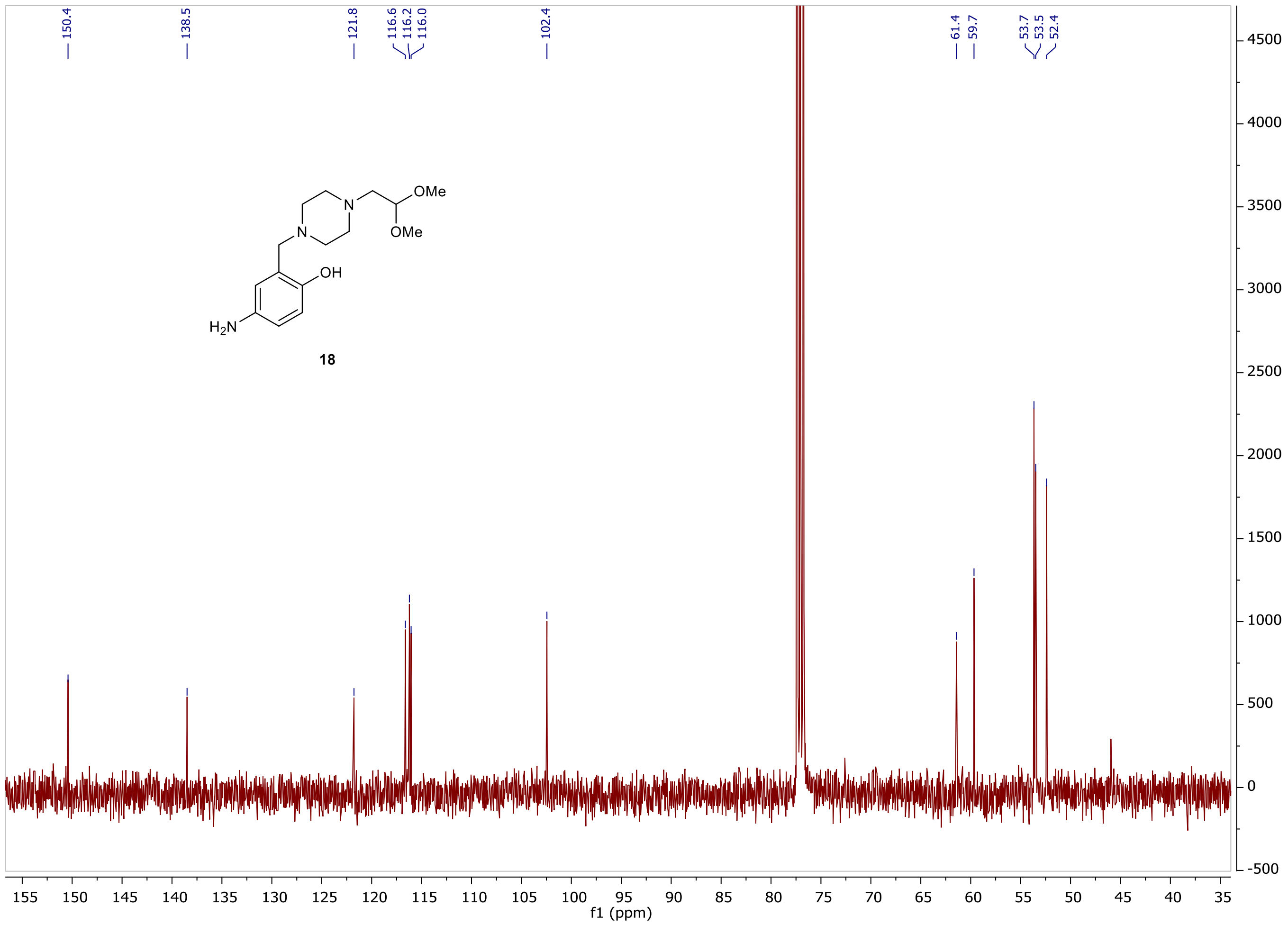
150.4
138.5
121.8
116.6
116.2
116.0
102.4
61.4
59.7
53.7
53.5
52.4
OMe
N
N
OMe
OH
H2N
18
4500
4000
3500
3000
2500
2000
1500
1000
500
0
-500
155 150 145 140 135 130 125 120 115 110 105 100 95 90 85 80 75 70 65 60 55 50 45 40 35
f1 (ppm)

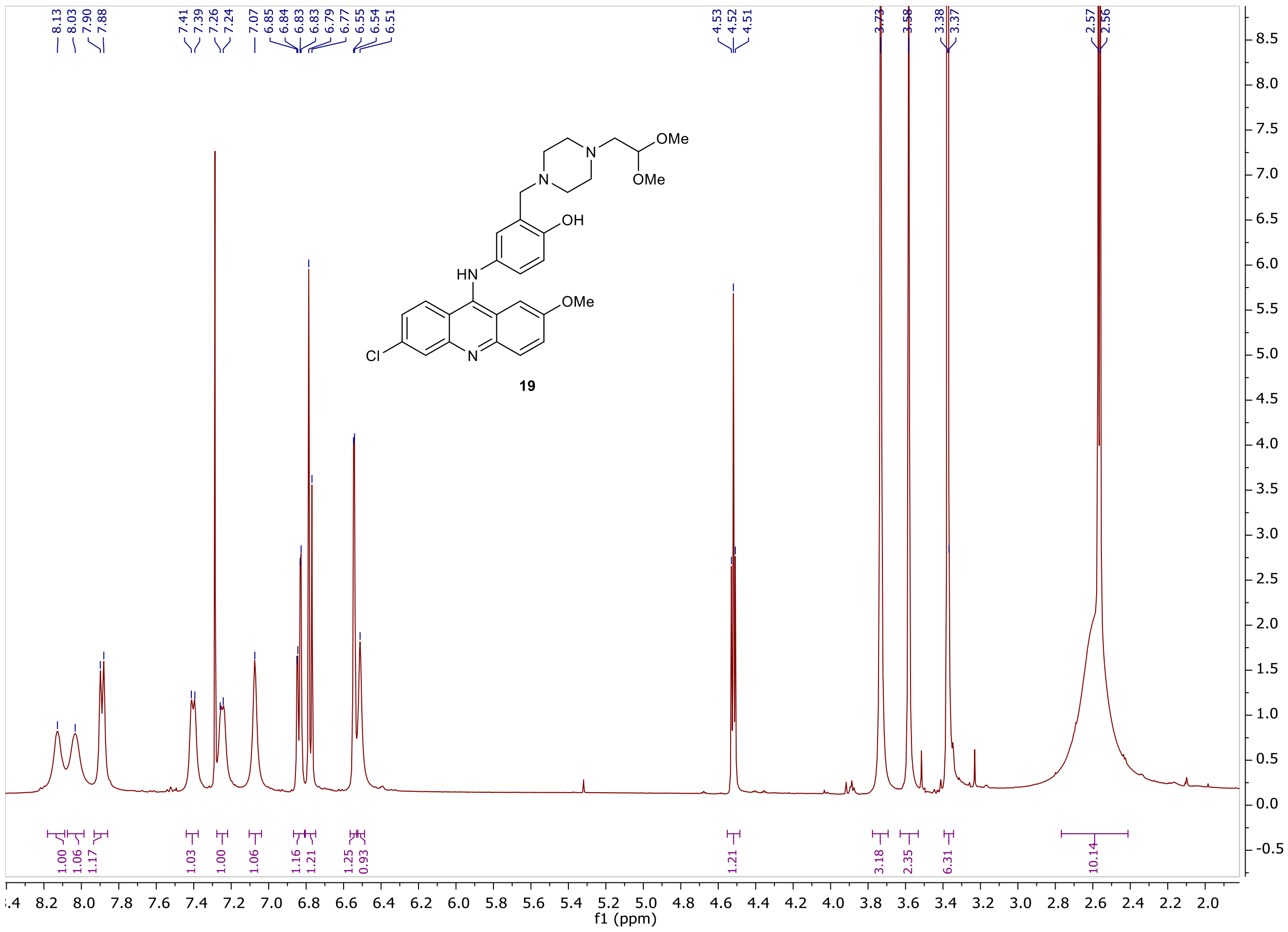
8.13
8.03
7.90
7.88
7.41
7.39
7.26
7.24
7.07
6.85
6.84
6.83
6.83
6.79
6.77
6.55
6.54
6.51
4.53
4.52
4.51
3.73
3.58
3.38
3.37
2.57
2.56
OMe
N
N
OMe
OH
HN
OMe
Cl
N
19
1.00
1.06
1.17
1.03
1.00
1.06
1.16
1.21
1.25
0.93
1.21
3.18
2.35
6.31
10.14
f1 (ppm)

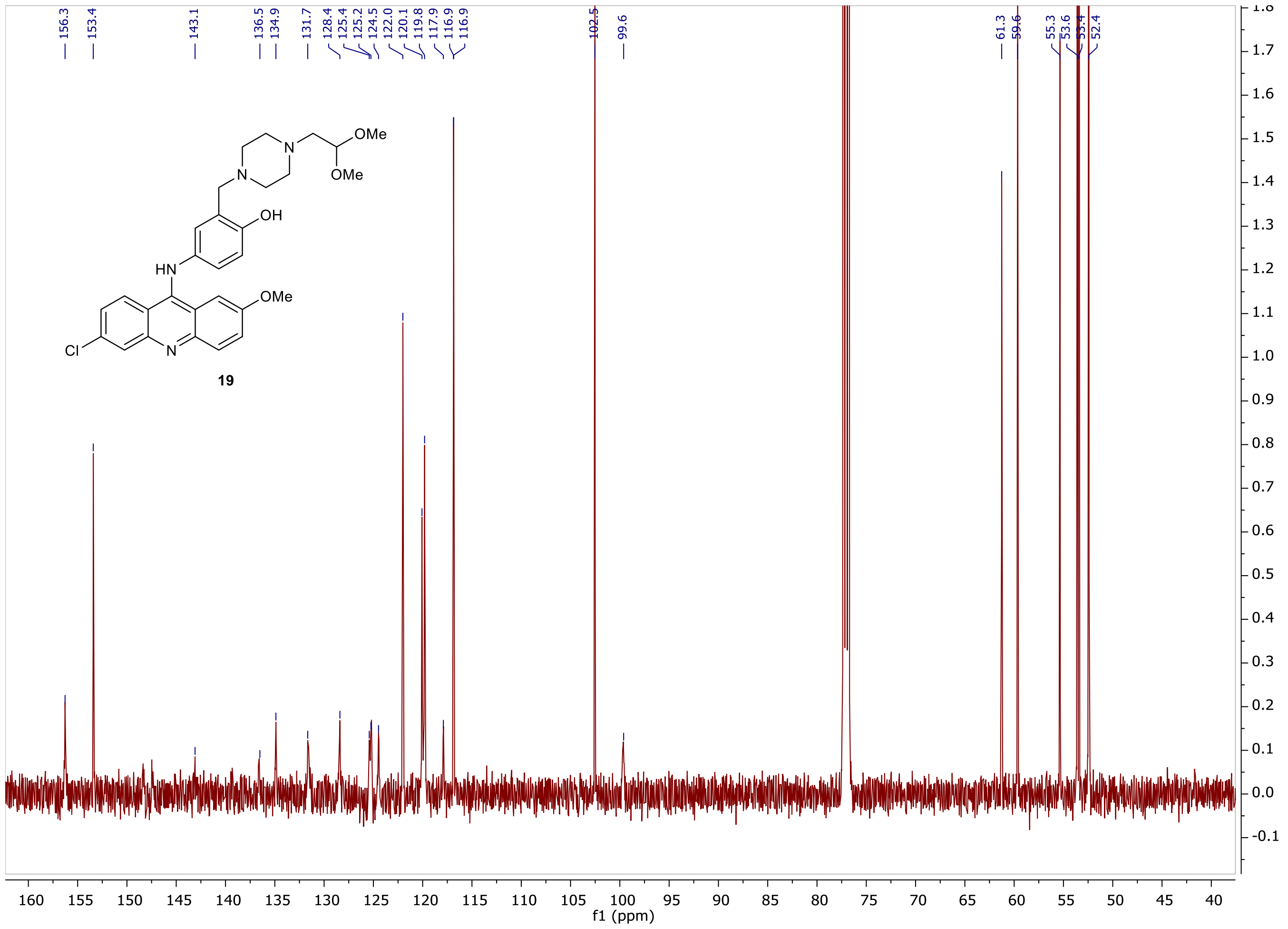
156.3
153.4
143.1
136.5
134.9
131.7
128.4
125.4
125.2
124.5
122.0
120.1
119.8
117.9
116.9
116.9
102.5
99.6
61.3
59.6
55.3
53.6
53.4
52.4
OMe
OMe
N
N
OH
HN
OMe
Cl
N
19
f1 (ppm)

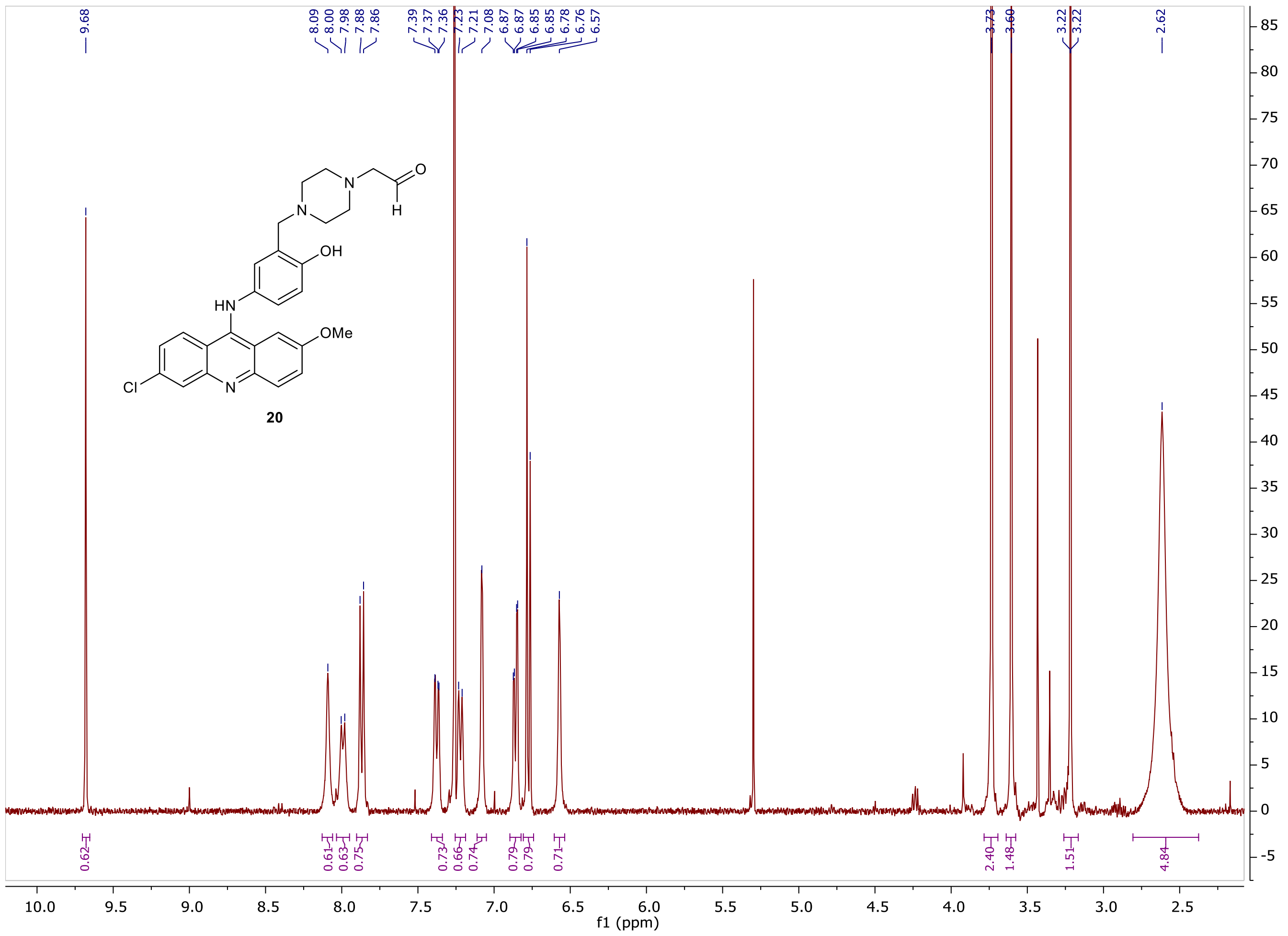

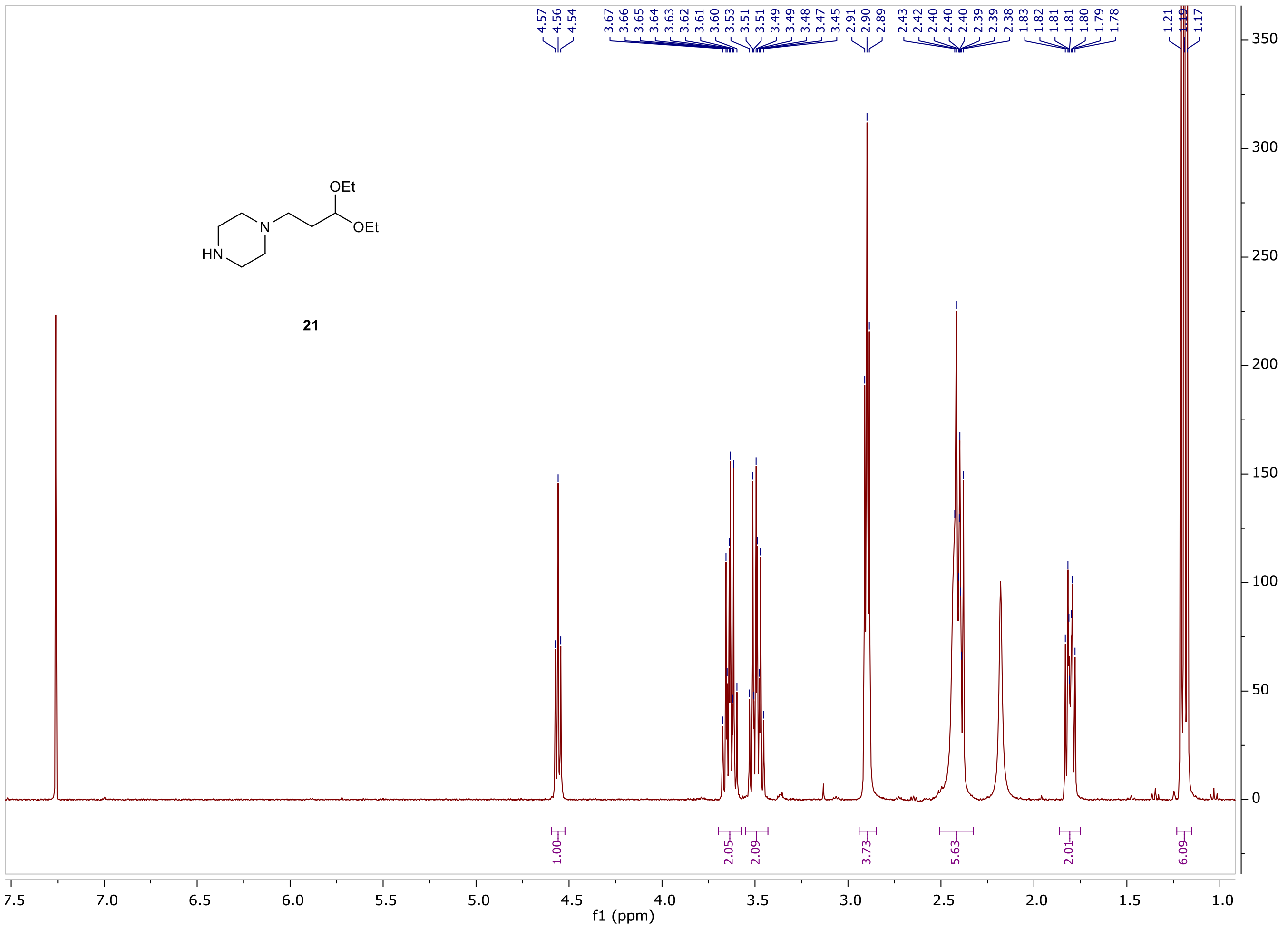
OEt
OEt
HN
N
21
f1 (ppm)

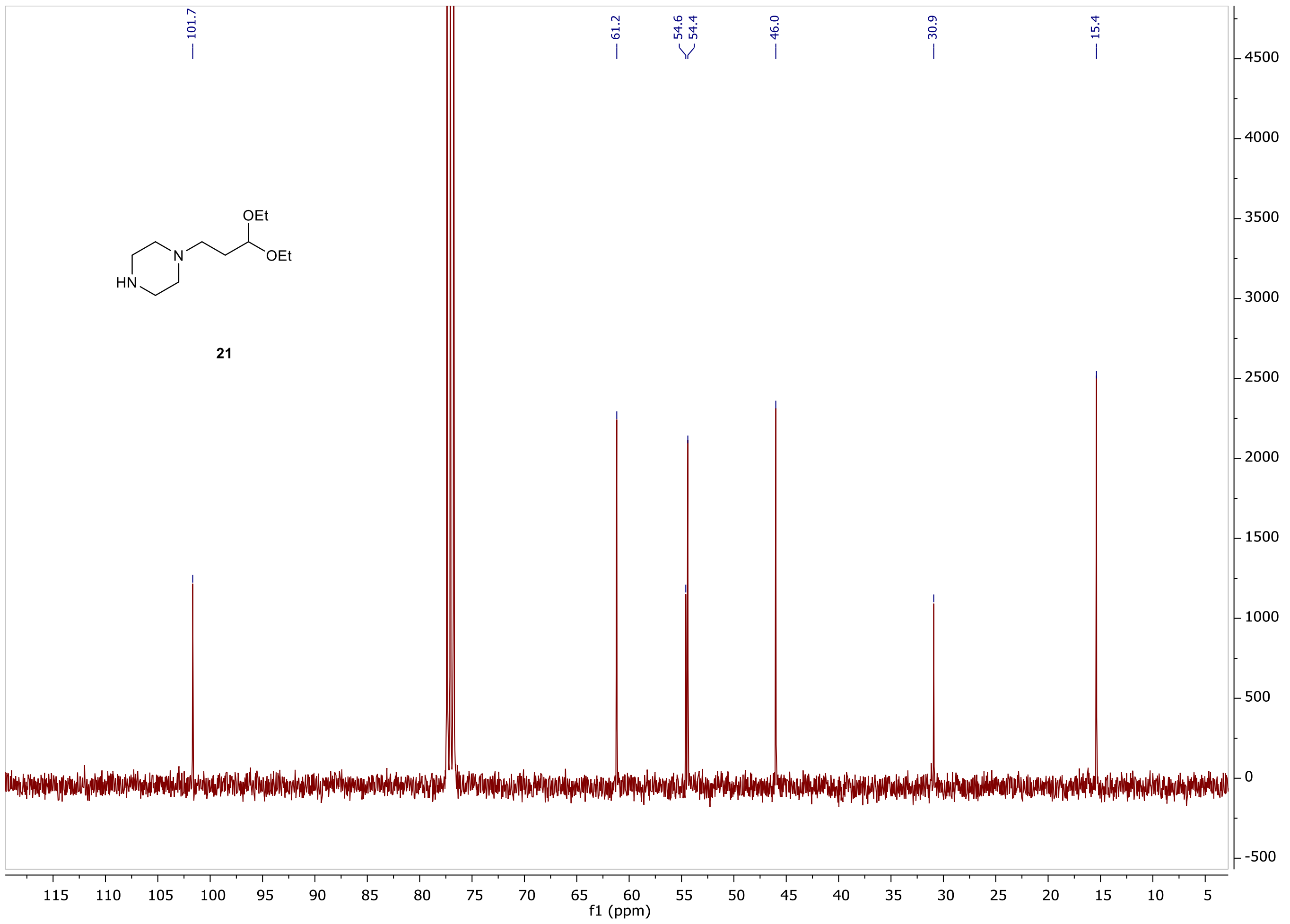
101.7
61.2
54.6
54.4
46.0
30.9
15.4
OEt
OEt
N
HN
21
f1 (ppm)

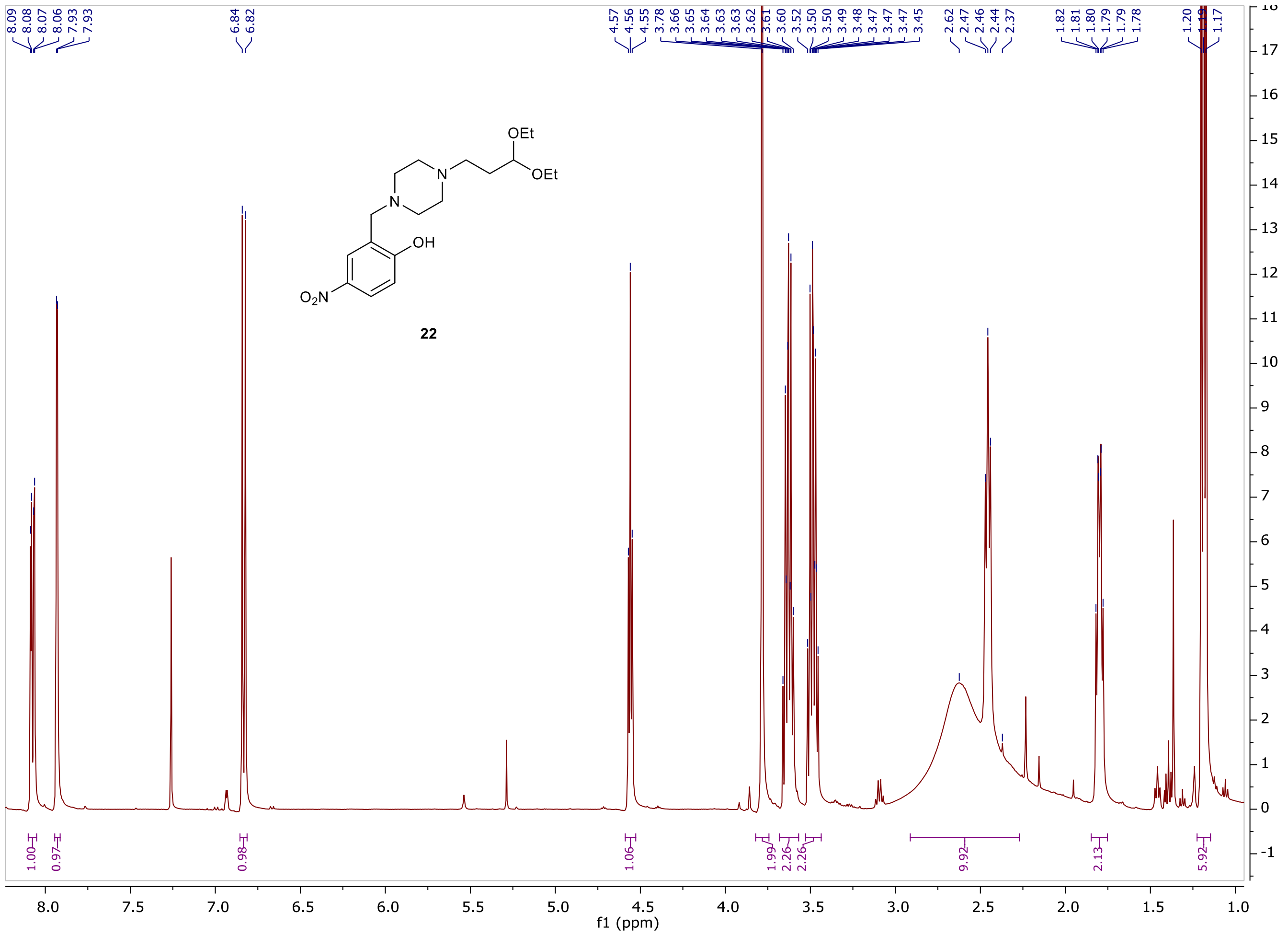
8.09
8.08
8.07
8.06
7.93
7.93
6.84
6.82
4.57
4.56
4.55
3.78
3.66
3.65
3.64
3.63
3.63
3.62
3.61
3.60
3.52
3.50
3.50
3.49
3.48
3.47
3.47
3.47
3.45
2.62
2.47
2.46
2.44
2.37
1.82
1.81
1.80
1.79
1.79
1.78
1.20
1.19
1.17
OEt
OEt
N
N
OH
O_2N
22
1.00
0.97
0.98
1.06
1.99
2.26
2.26
9.92
2.13
5.92
f1 (ppm)

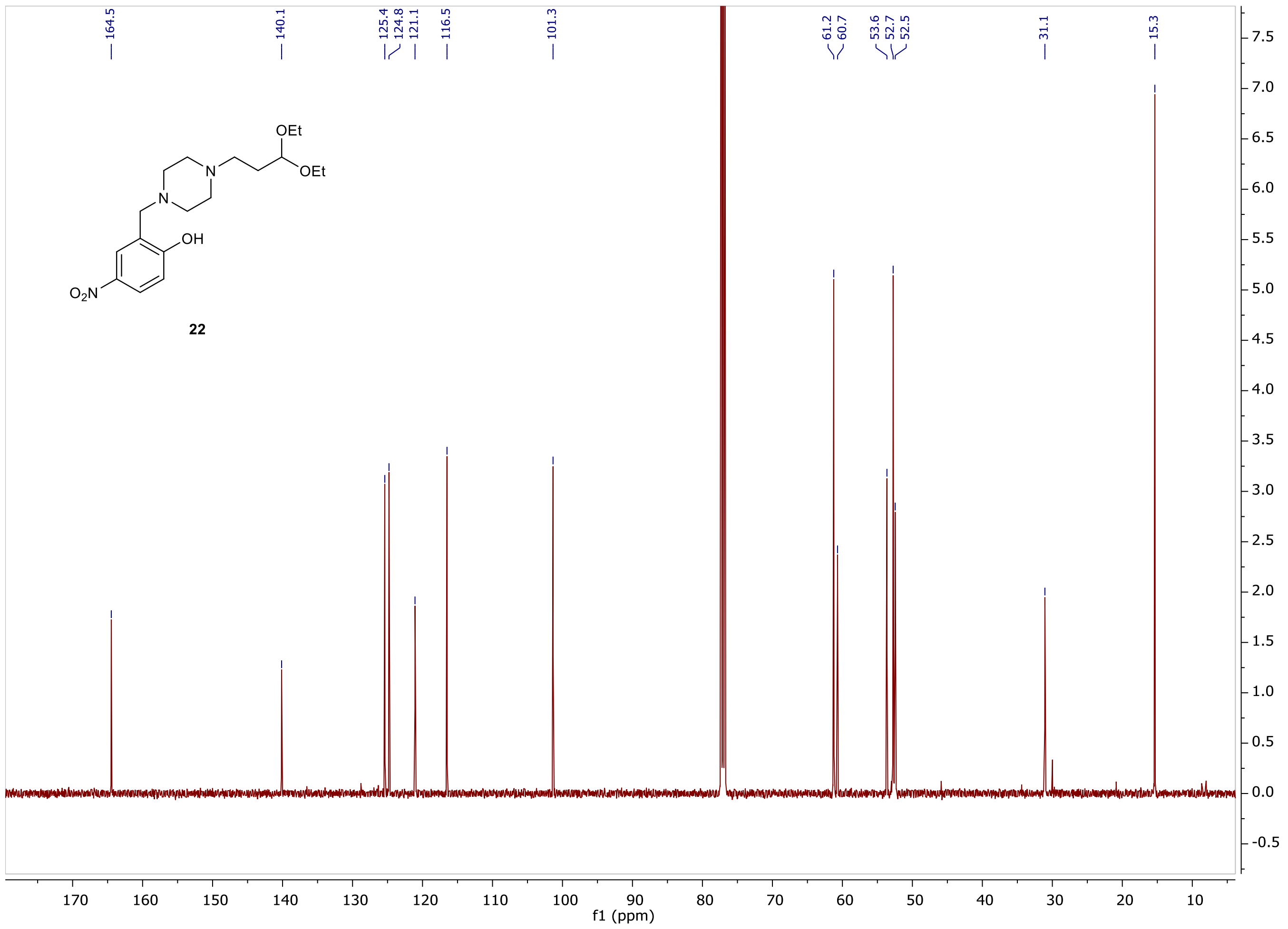
164.5
140.1
125.4
124.8
121.1
116.5
101.3
61.2
60.7
53.6
52.7
52.5
31.1
15.3
OEt
OEt
N
N
OH
O2N
f1 (ppm)

22

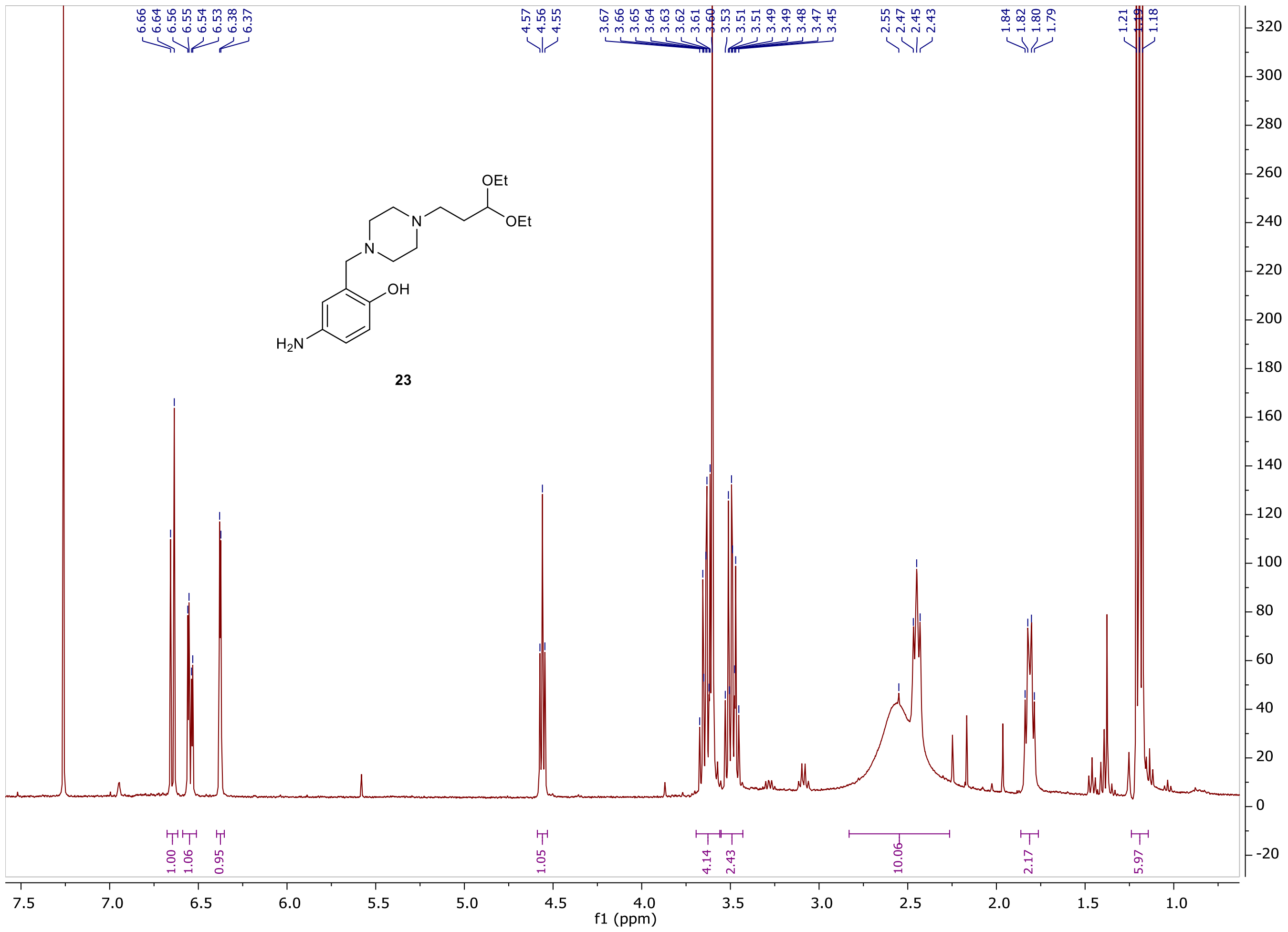

23

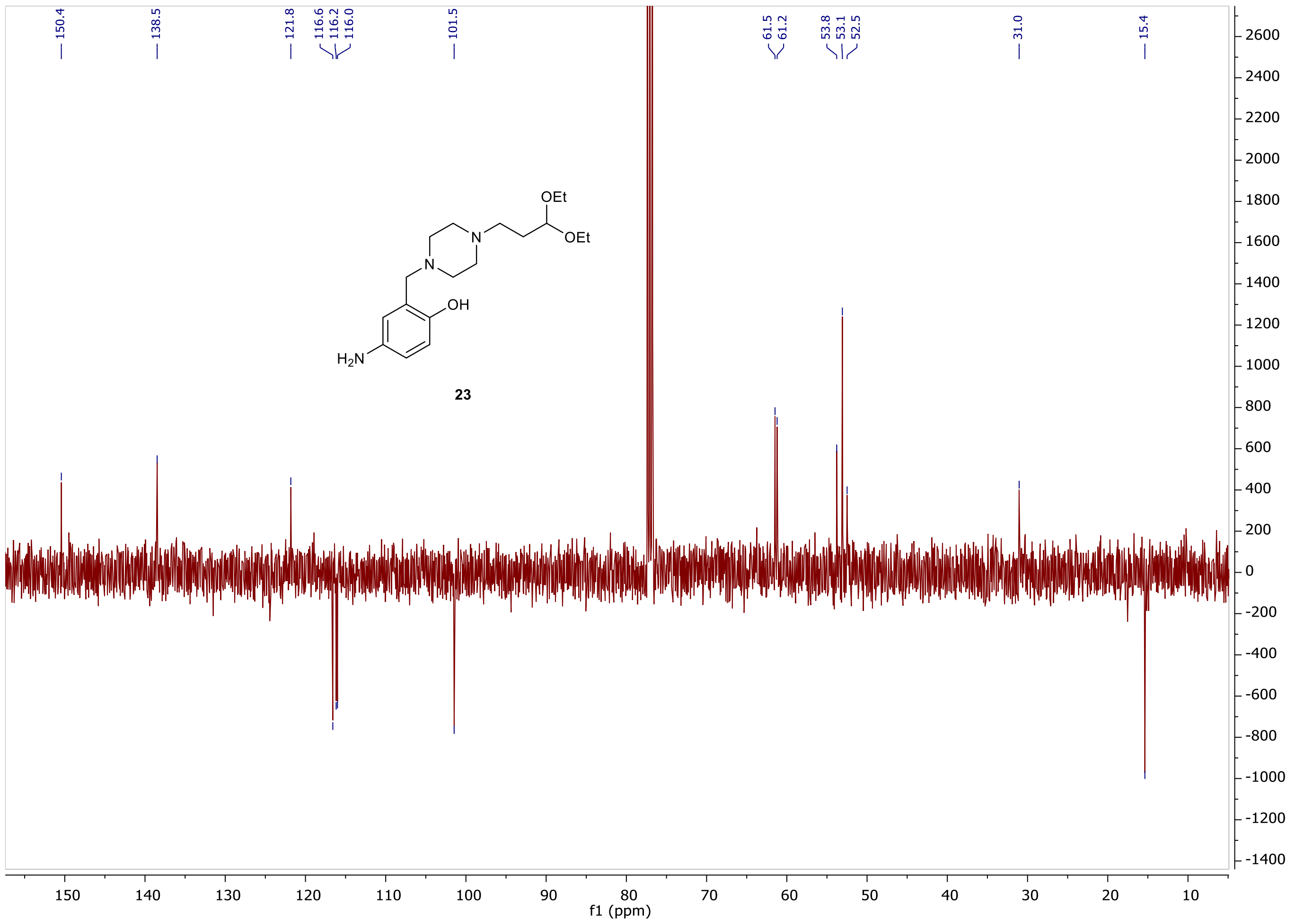
150.4
138.5
121.8
116.6
116.2
116.0
101.5
61.5
61.2
53.8
53.1
52.5
31.0
15.4
OEt
OEt
N
N
OH
H2N
23
f1 (ppm)

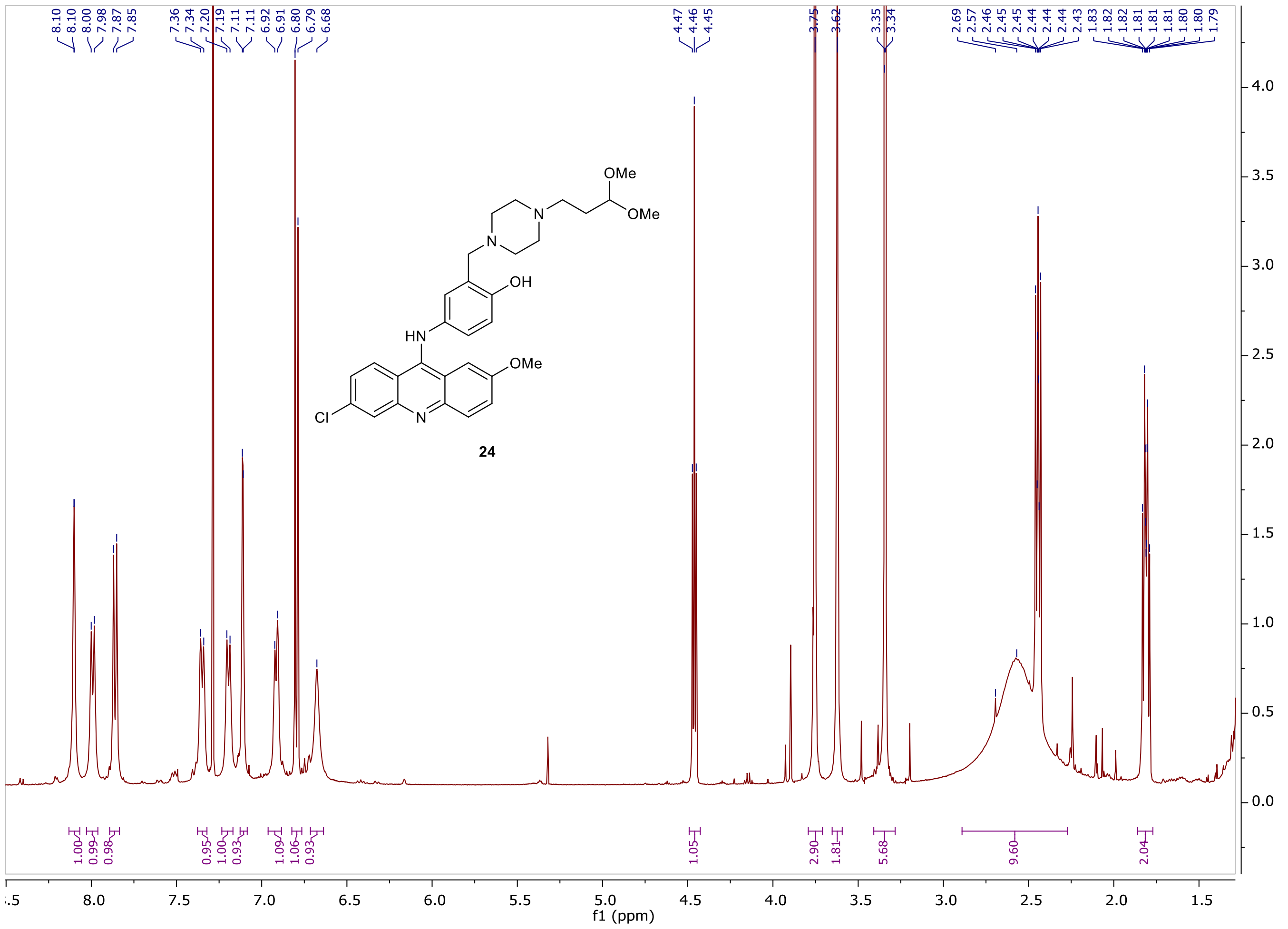
24
f1 (ppm)

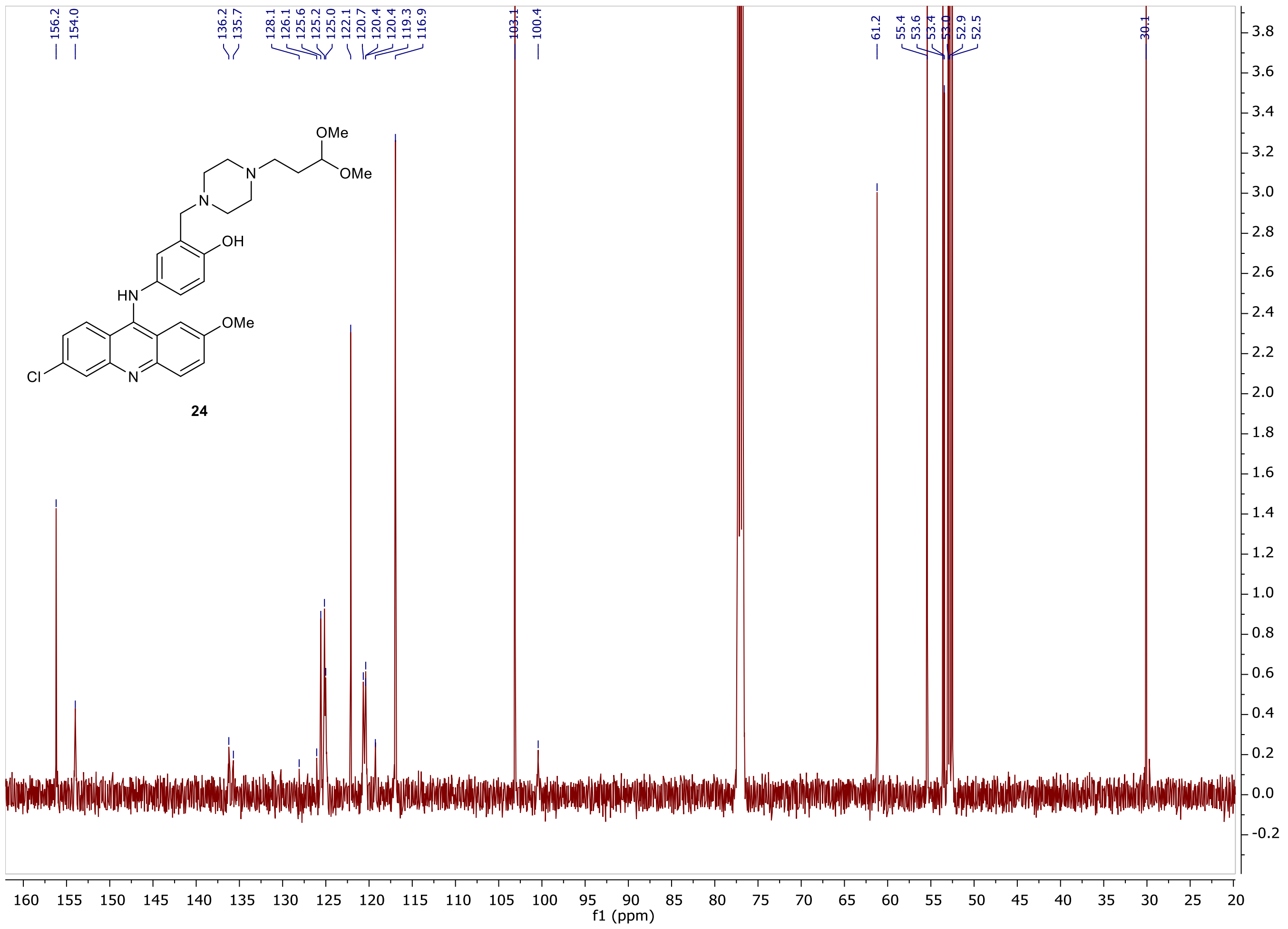
156.2
154.0
136.2
135.7
128.1
126.1
125.6
125.2
125.0
122.1
120.7
120.4
120.4
119.3
116.9
103.1
100.4
61.2
55.4
53.6
53.4
53.0
52.9
52.5
30.1
OMe
OMe
N
N
OH
HN
OMe
Cl
N
24
f1 (ppm)

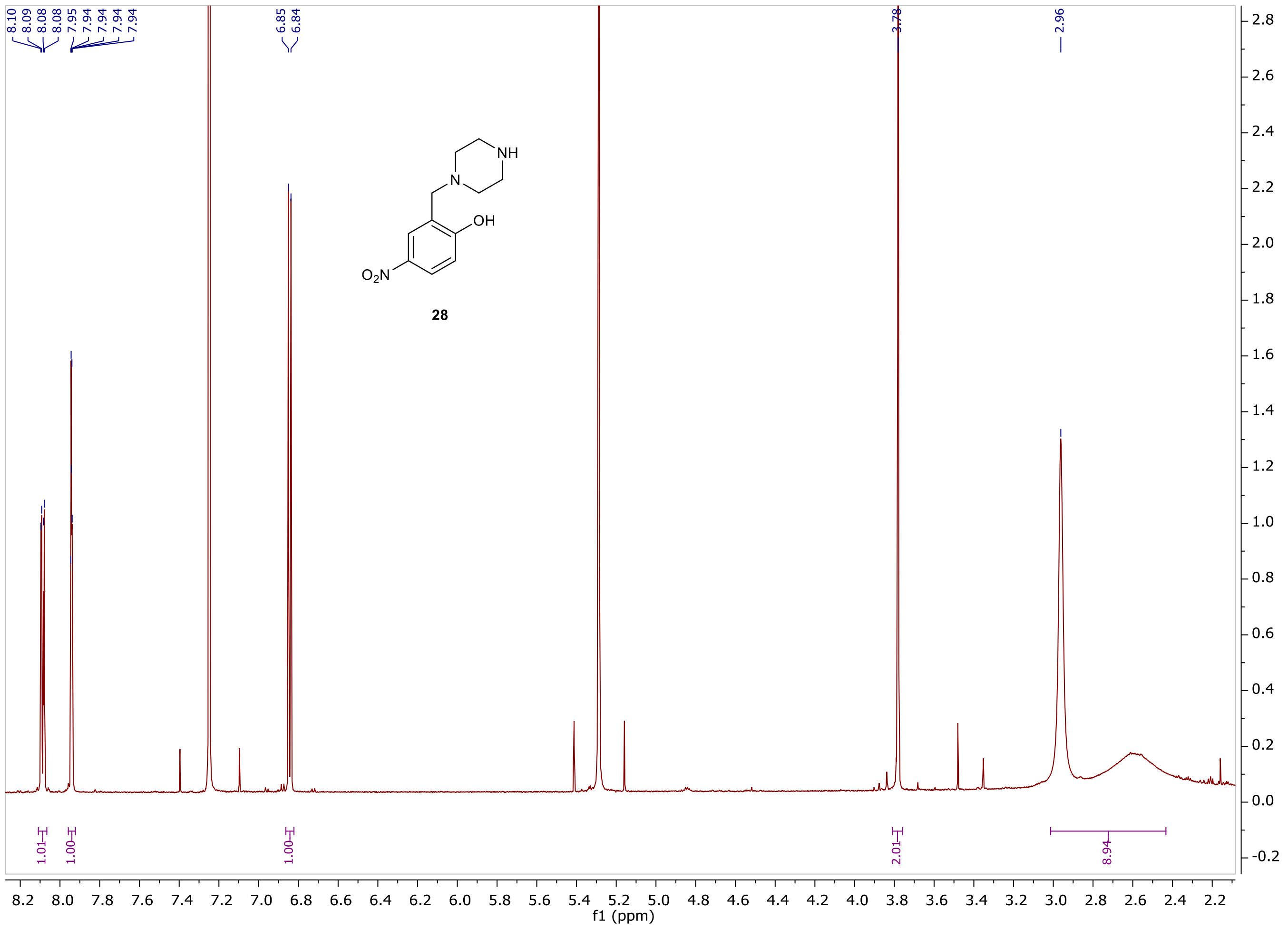
8.10
8.09
8.08
8.08
7.95
7.94
7.94
7.94
7.94
6.85
6.84
3.78
2.96
NH
N
OH
O_2N
28
1.01
1.00
1.00
2.01
8.94
f1 (ppm)

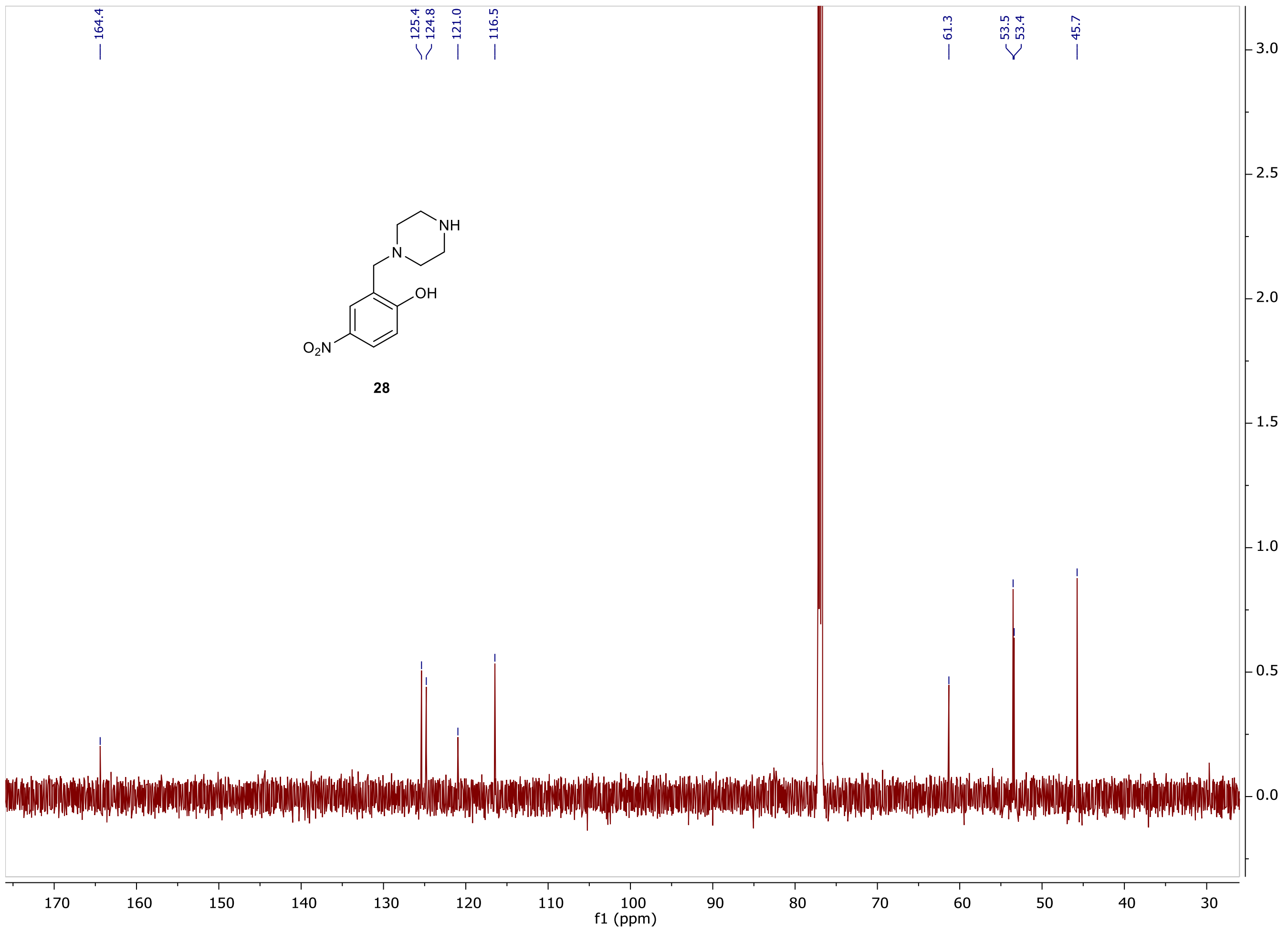
164.4
125.4
124.8
121.0
116.5
61.3
53.5
53.4
45.7
NH
N
OH
O2N
28
3.0
2.5
2.0
1.5
1.0
0.5
0.0
170
160
150
140
130
120
110
100
90
80
70
60
50
40
30
f1 (ppm)

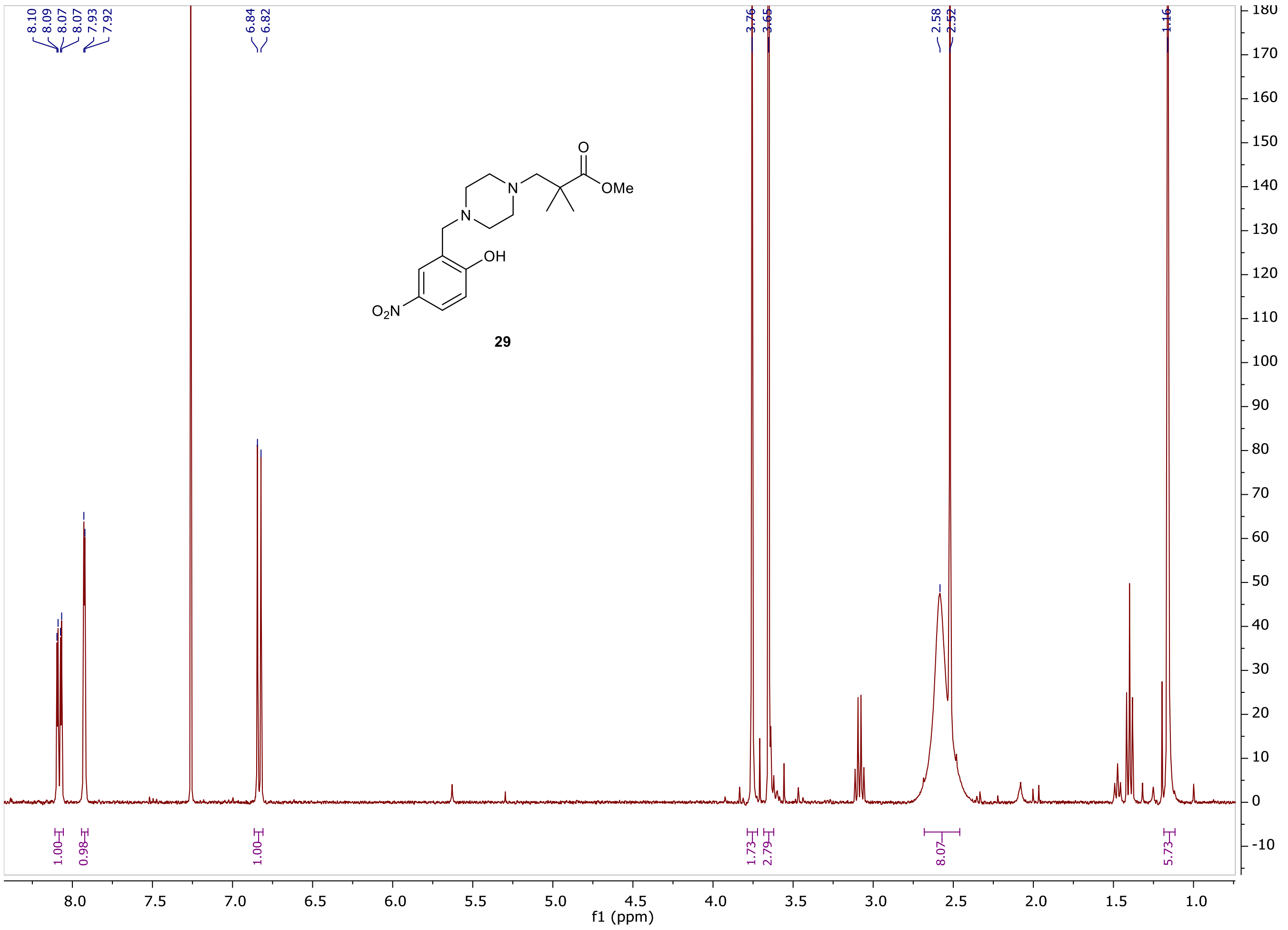
8.10
8.09
8.07
8.07
7.93
7.92
6.84
6.82
3.76
3.65
2.58
2.52
1.16
OH
O
OMe
N
N
O2N
29
1.00
0.98
1.00
1.73
2.79
8.07
5.73
f1 (ppm)

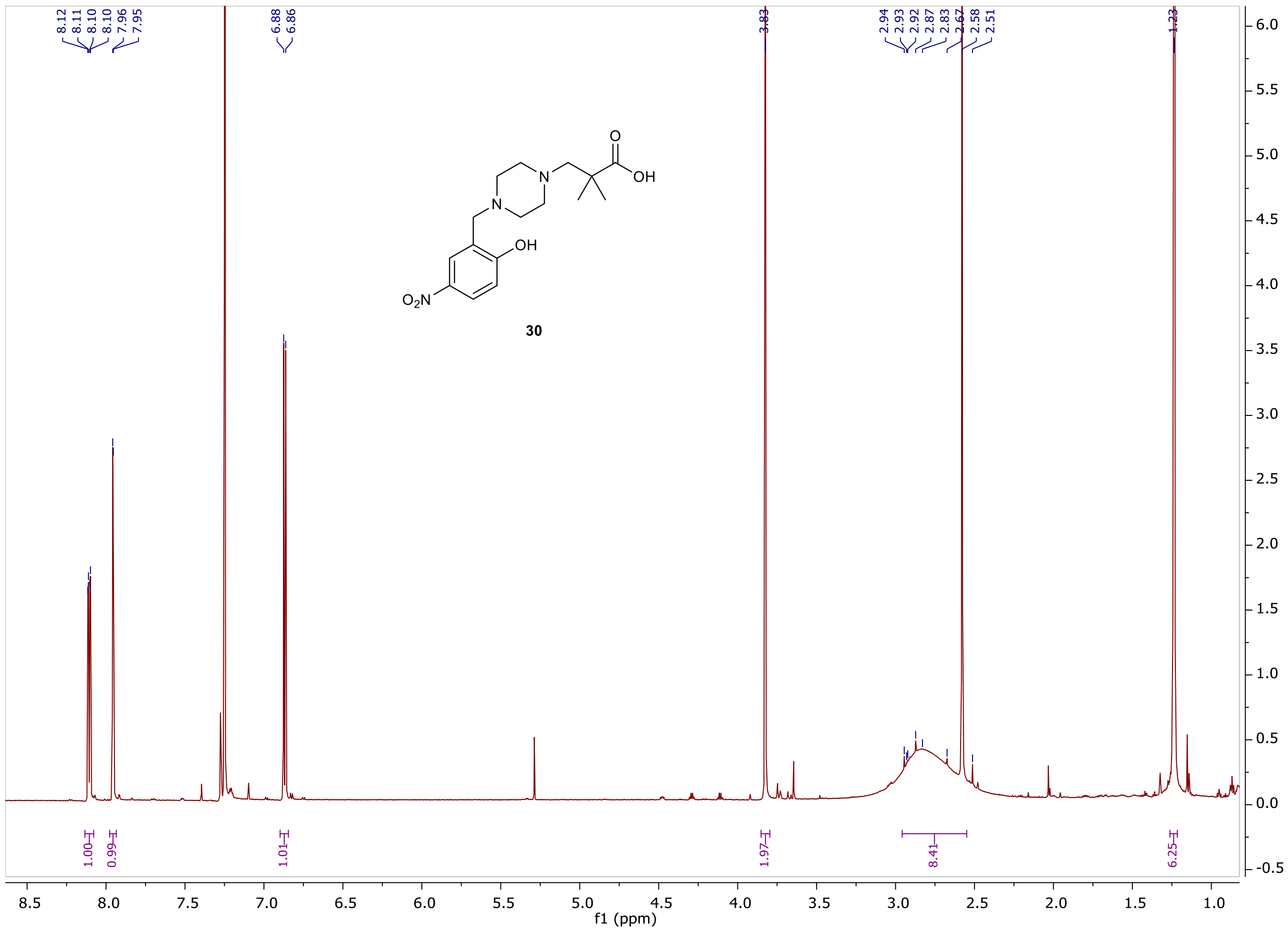
8.12
8.11
8.10
8.10
7.96
7.95
6.88
6.86
3.83
2.94
2.93
2.92
2.87
2.83
2.67
2.58
2.51
1.22
O
OH
N
N
OH
O2N
30
1.00
0.99
1.01
1.97
8.41
6.25
f1 (ppm)

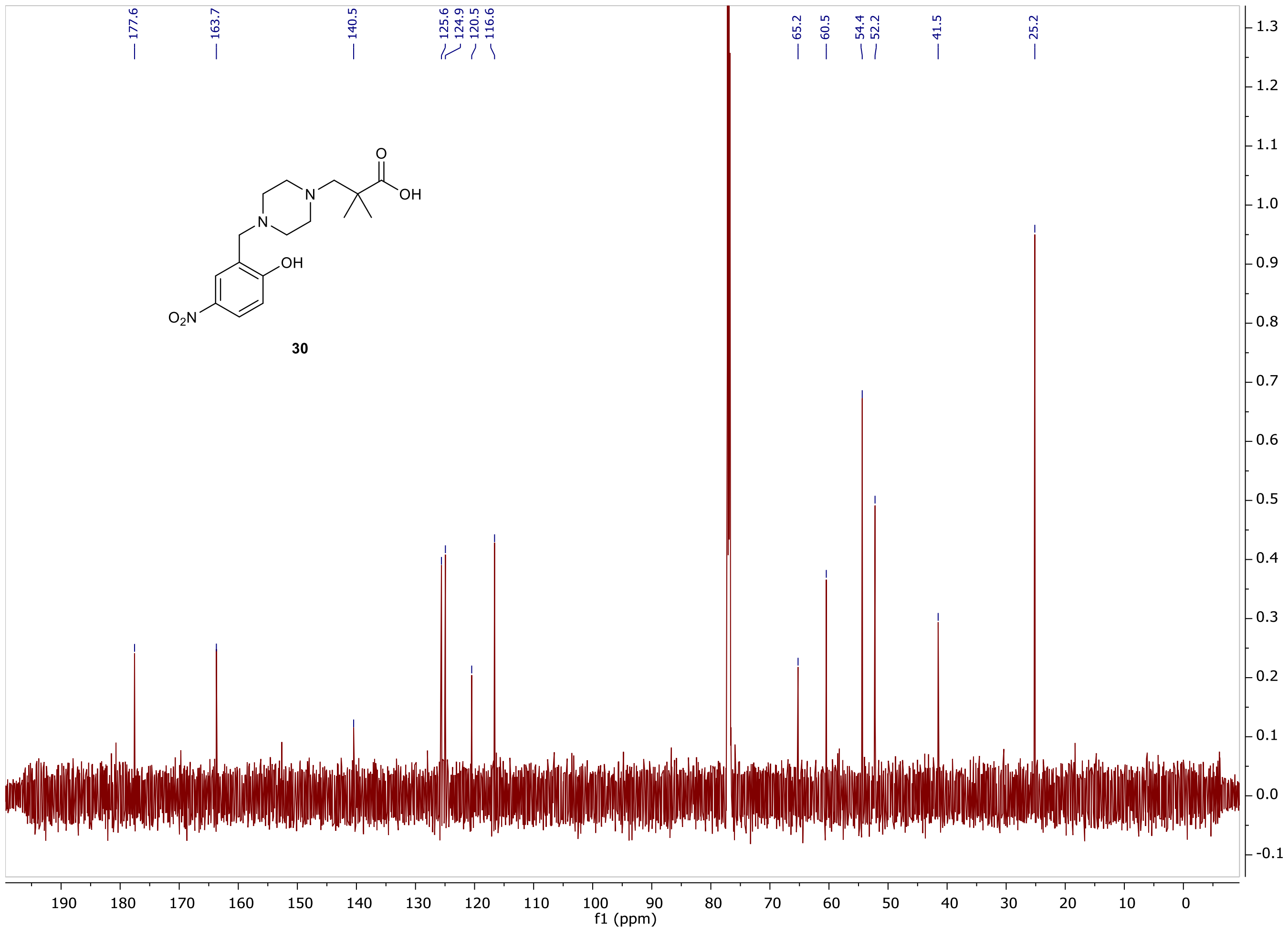
177.6
163.7
140.5
125.6
124.9
120.5
116.6
65.2
60.5
54.4
52.2
41.5
25.2
O
OH
N
N
OH
O2N
30
f1 (ppm)

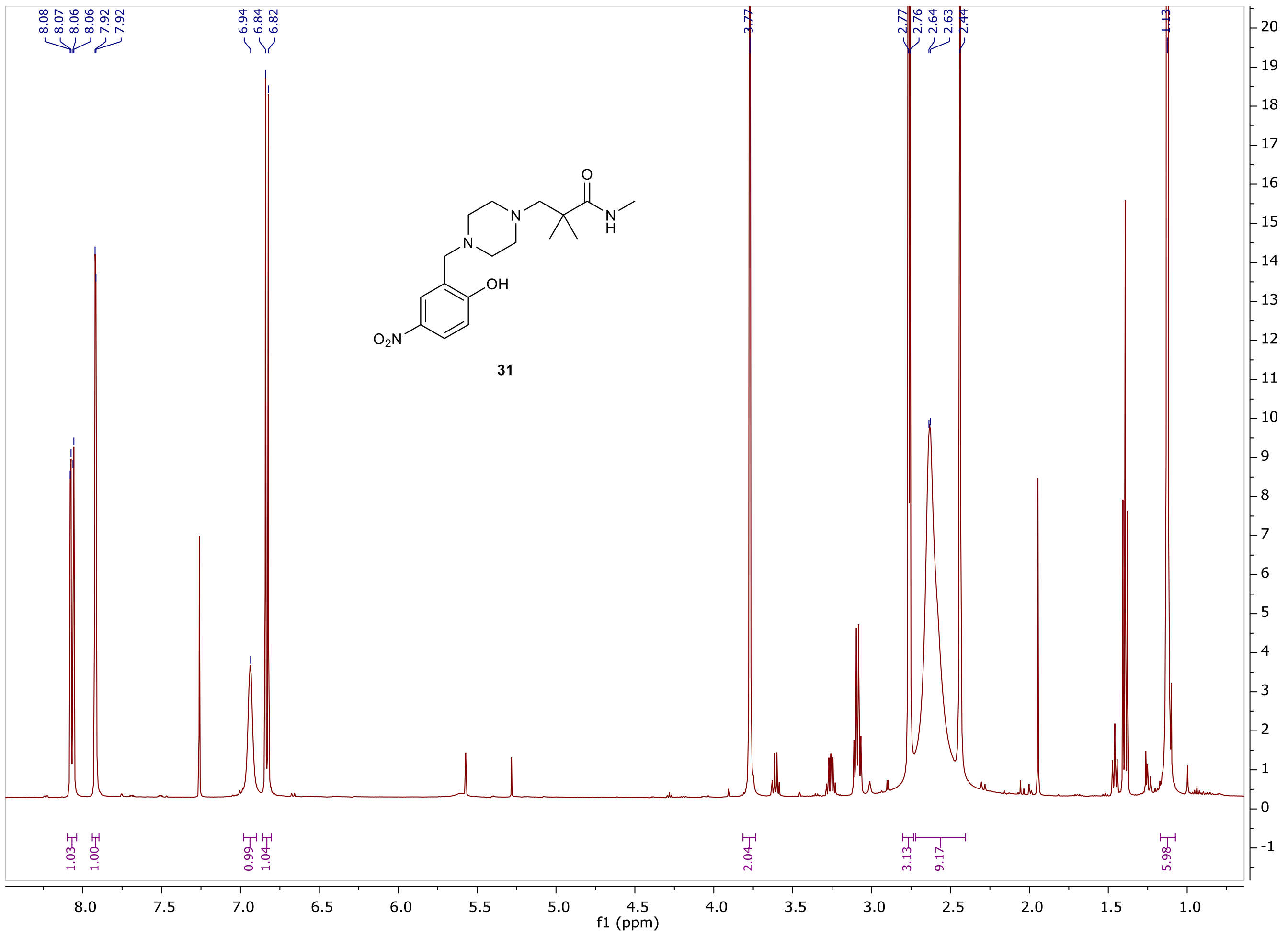
8.08
8.07
8.06
8.06
7.92
7.92
6.94
6.84
6.82
3.77
2.77
2.76
2.64
2.63
2.44
1.13
OH
O
N
H
O_2N
31
1.03
1.00
0.99
1.04
2.04
3.13
9.17
5.98
f1 (ppm)

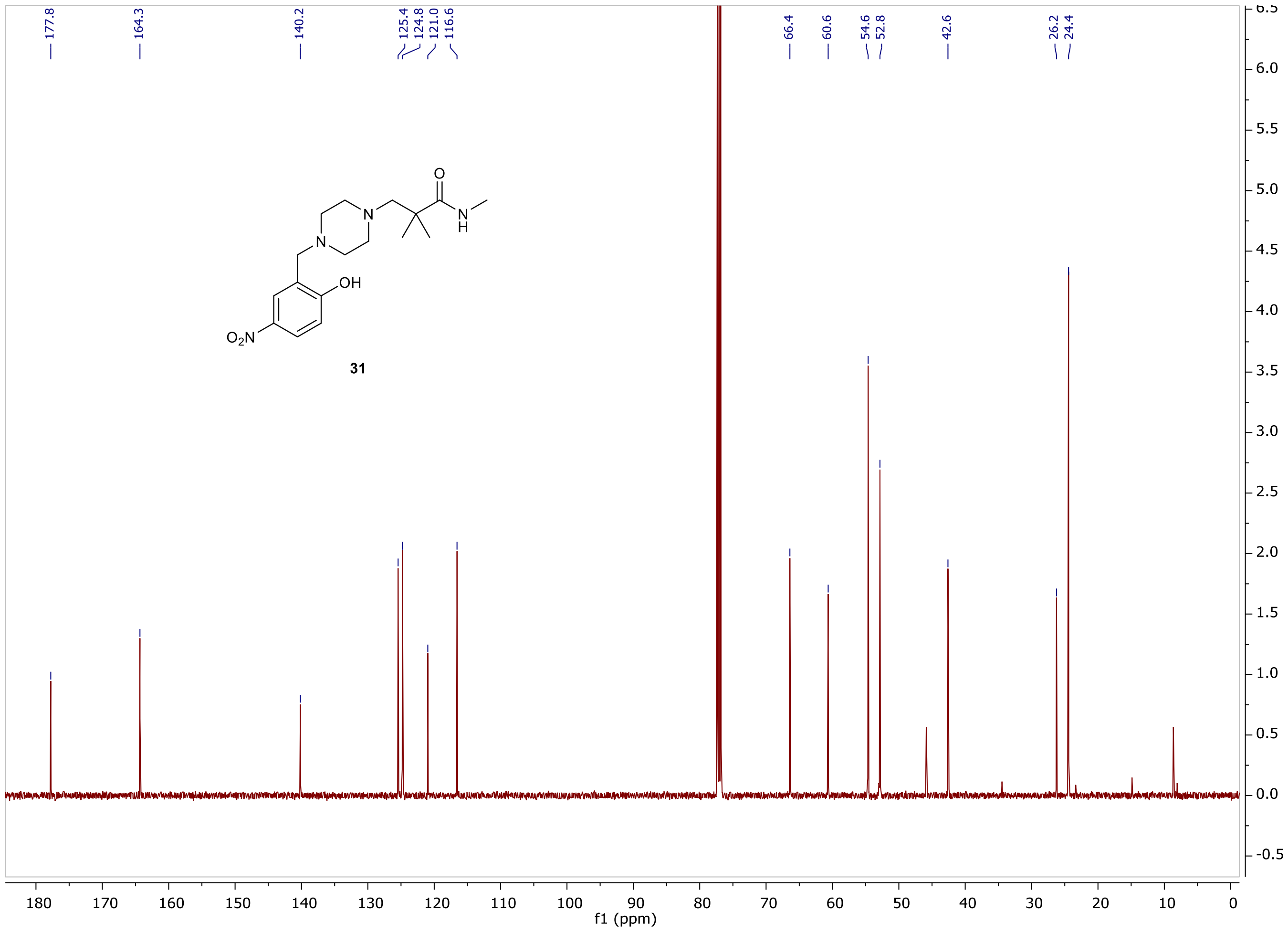
177.8
164.3
140.2
125.4
124.8
121.0
116.6
66.4
60.6
54.6
52.8
42.6
26.2
24.4
O
N
H
N
N
OH
O2N
31
f1 (ppm)

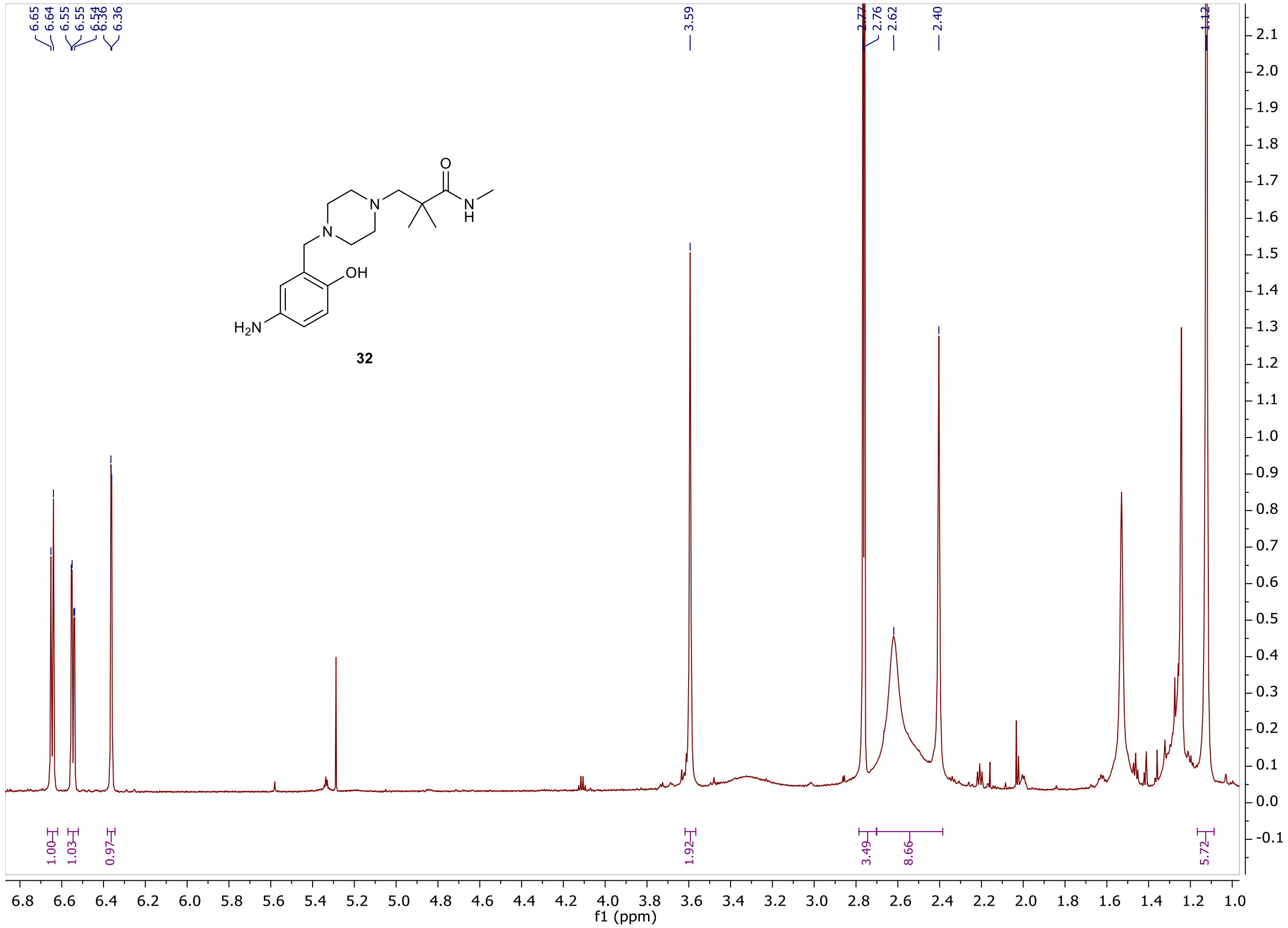
6.65
6.64
6.55
6.55
6.54
6.36
6.36
3.59
2.77
2.76
2.62
2.40
1.12
OH
H2N
N
N
O
N
H
32
1.00
1.03
0.97
1.92
3.49
8.66
5.72
f1 (ppm)

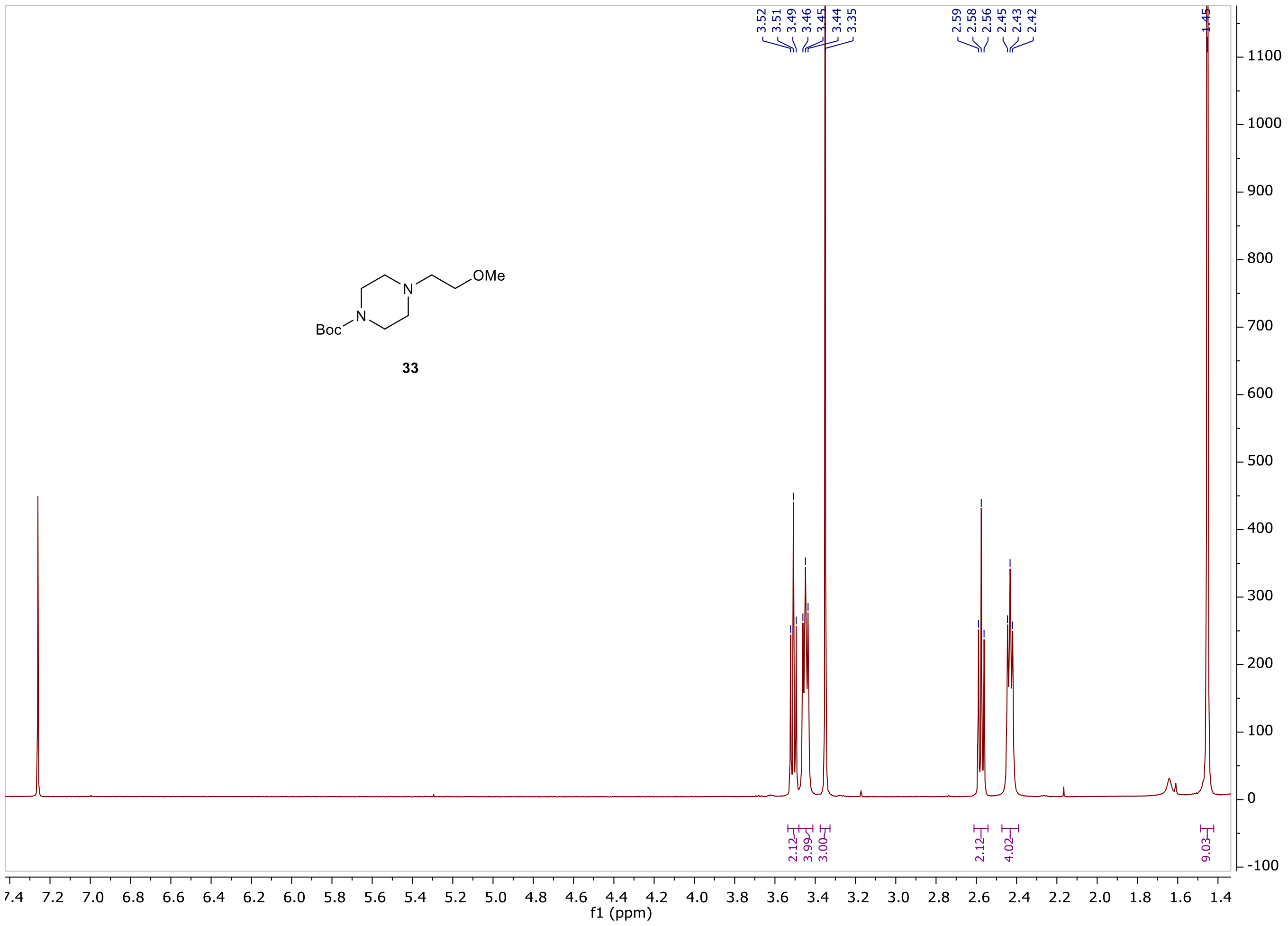
3.52
3.51
3.49
3.46
3.45
3.44
3.35
2.59
2.58
2.56
2.45
2.43
2.42
1.45
OMe
N
Boc
N
33
2.12
3.99
3.00
2.12
4.02
9.03
f1 (ppm)

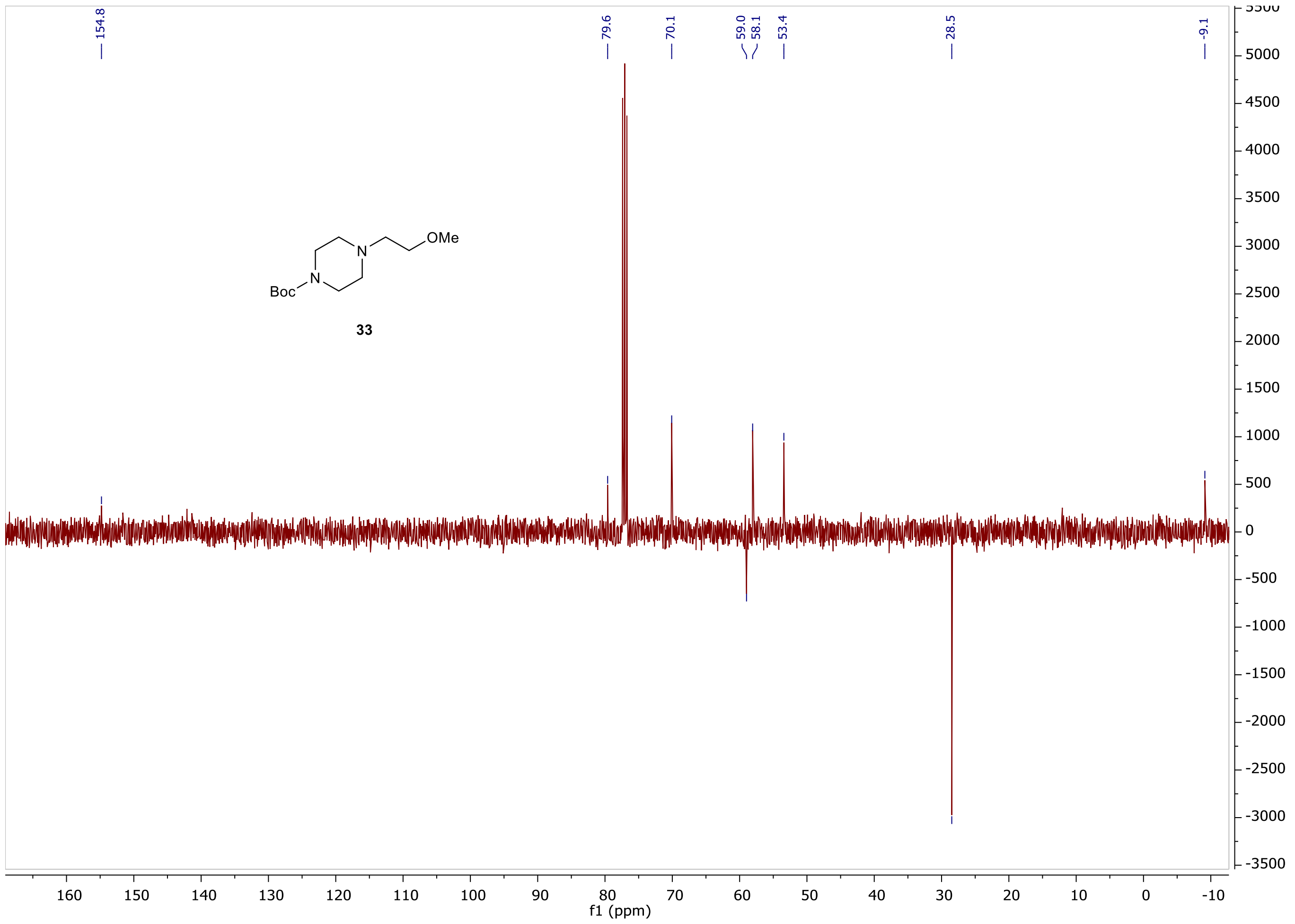
154.8
79.6
70.1
59.0
58.1
53.4
28.5
-9.1
Boc
N
N
OMe
33
f1 (ppm)

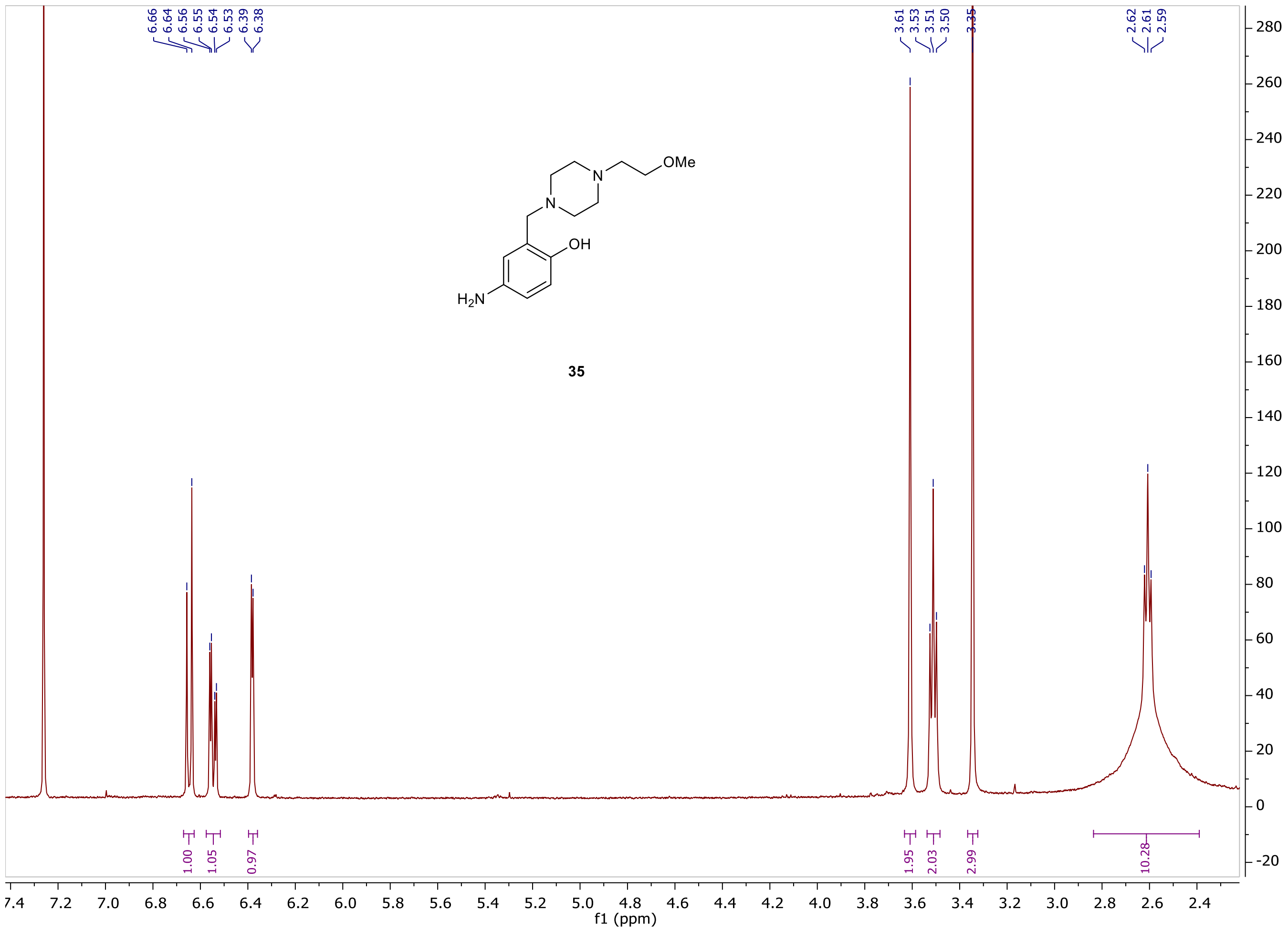
6.66
6.64
6.56
6.55
6.54
6.53
6.39
6.38
3.61
3.53
3.51
3.50
3.35
2.62
2.61
2.59
OMe
N
N
OH
H2N
35
1.00
1.05
0.97
1.95
2.03
2.99
10.28
f1 (ppm)
